# The Effect of Improved Access to Family Planning on Postpartum Women: Protocol for a Randomized Controlled Trial

**DOI:** 10.2196/16697

**Published:** 2020-08-14

**Authors:** Mahesh Karra, David Canning

**Affiliations:** 1 Frederick S Pardee School of Global Studies Boston University Boston, MA United States; 2 Department of Global Health and Population Harvard T H Chan School of Public Health Harvard University Boston, MA United States

**Keywords:** postpartum family planning, contraceptive use, birth spacing, women’s well-being, randomized controlled trial, Malawi, Sub-Saharan Africa

## Abstract

**Background:**

The World Health Organization recommends that a woman waits at least 24 months after a live birth before getting pregnant again; however, an estimated 25% of birth intervals in low-income countries do not meet this recommendation for adequate birth spacing, and the unmet need for postpartum family planning (PPFP) services is high. Few randomized controlled trials have assessed the causal impact of access to PPFP services, and even fewer evaluations have investigated how such interventions may affect postpartum contraceptive use, birth spacing, and measures of health and well-being.

**Objective:**

This protocol paper aims to describe a randomized controlled trial that is being conducted to identify the causal impact of an intervention to improve access to PPFP services on contraceptive use, pregnancy, and birth spacing in urban Malawi. The causal effect of the intervention will be determined by comparing outcomes for respondents who are randomly assigned to an intervention arm against outcomes for respondents who are randomly assigned to a control arm.

**Methods:**

Married women aged 18-35 years who were either pregnant or had recently given birth were randomly assigned to either the intervention arm or control arm. Women assigned to the intervention arm received a package of services over a 2-year intervention period. Services included a brochure and up to 6 home visits from trained family planning counselors; free transportation to a high-quality family planning clinic; and financial reimbursement for family planning services, consultations, and referrals for services. Two follow-up surveys were conducted 1 and 2 years after the baseline survey.

**Results:**

A total of 2143 women were randomly assigned to either the intervention arm (n=1026) or the control arm (n=1117). Data collection for the first follow-up survey began in August 2017 and was completed in February 2018. A total of 1773 women, or 82.73% of women who were eligible for follow-up, were successfully contacted and reinterviewed at the first follow-up. Data collection for the second follow-up survey began in August 2018 and was completed in February 2019. A total of 1669 women, or 77.88% of women who were eligible for follow-up, were successfully contacted and reinterviewed at the second follow-up. The analysis of the primary outcomes is ongoing and is expected to be completed in 2021.

**Conclusions:**

The results of this trial seek to fill the current knowledge gaps in the effectiveness of family planning interventions on improving fertility and health outcomes. The findings also show that the benefits of improving access to family planning are likely to extend beyond the fertility and health domain by improving other measures of women’s well-being.

**Trial Registration:**

American Economics Association Registry Trial Number AEARCTR-0000697; https://www.socialscienceregistry.org/trials/697 Registry for International Development Impact Evaluations (RIDIE) Trial Number RIDIE-STUDY-ID-556784ed86956; https://ridie.3ieimpact.org/index.php?r=search/detailView&id=320

**International Registered Report Identifier (IRRID):**

DERR1-10.2196/16697

## Introduction

### The Role of Family Planning

The World Health Organization (WHO) guidelines recommend that a woman wait at least 24 months after a live birth before getting pregnant again [1,2]. Poorly spaced births may contribute to higher rates of mortality for both mothers and infants [3,4]; however, an estimated 25% of birth intervals in low- and middle-income countries do not meet the WHO’s 24-month recommended guideline for adequate birth spacing [5]. This gap between recommended spacing and realized spacing highlights the importance of postpartum family planning (PPFP), particularly in sub-Saharan Africa where the unmet need for PPFP is high. Given that the ideal family size is higher among women in sub-Saharan Africa than in other parts of the world, the demand for and use of family planning is driven more from a desire to space future births rather than to limit births. Nevertheless, an estimated 8 million women in sub-Saharan Africa have an unmet need for limiting future births [6]. The continuing high unmet need for and lack of access to PPFP highlights the need to mobilize efforts toward meeting the family planning and fertility goals of postpartum women. To this end, interventions that aim to influence the demand and supply of PPFP have become increasingly common in developing countries. These interventions have targeted key populations in a variety of ways, from education and awareness programs in schools to multicomponent, community-based campaigns [7,8].

Recently, the number of family planning interventions that have undergone more rigorous impact evaluation has increased to assess the effects of family planning on fertility, health behavior, and health outcomes. However, findings from community-level social programs such as the Maternal and Child Health–Family Planning Extensions project in Matlab, Bangladesh, and the Navrongo experiment in Ghana have also shown that contraceptive use declines considerably following the discontinuation of family planning services [9-12]. Although not all the studies focused on postpartum women, these results suggest that increased access to high-quality family planning services, particularly for new mothers, needs to be expanded beyond the neonatal clinic. To this end, few randomized controlled trials have been conducted to assess the causal impact of family planning in low-income countries, and even fewer impact evaluations have been conducted to determine the extent to which such family planning interventions may affect downstream health and development outcomes. To date, not many impact evaluations have sought to identify the effectiveness of family planning and reproductive health programs at the individual or household level; apart from the frequently cited Matlab quasi-experimental study and a recent field experiment by Ashraf et al [13], no randomized controlled trial, to the best of our knowledge, has attempted to causally identify the impact of family planning and birth spacing on both immediate and longer-term outcomes of health and well-being in sub-Saharan Africa.

### Study Objectives

To address these gaps in the evidence, we conducted a randomized controlled trial to identify the causal impact of improved access to PPFP. The study population included married postpartum women aged 18 to 35 years in Lilongwe, Malawi. As part of the trial, each woman in the study was randomly assigned to either the treatment or control arm. A woman who was assigned to the intervention arm received a 2-year–long family planning intervention that was designed to reduce key barriers to access for postpartum women in urban Malawi [14,15]. The primary objective of this study is to evaluate the impact of the family planning intervention on contraceptive use, fertility, birth spacing, and other measures of maternal and child health and well-being in postpartum women who received the family planning intervention (the intervention arm) compared with women who did not receive the intervention (the control group). Primary outcomes include short-term outcomes related to modern contraceptive use and contraceptive method mix (the profile of the relative level of use of different contraceptive methods within our study sample) as well as intermediate outcomes related to fertility and birth spacing. Shorter-term secondary outcomes include changes in desired fertility, unmet need for family planning, and outcomes associated with maternal and child health, including safe pregnancy, birth height and weight, and nutritional status. Longer-term secondary outcomes include educational attainment (matriculation rates and years of schooling completed), labor market outcomes (employment status, female labor supply, and women’s time use), and income. This study seeks to fill the current knowledge gaps on the effectiveness of family planning interventions by directly identifying the impact of an increase in access to family planning on fertility and health outcomes. A downstream objective of this study is to demonstrate that the benefits of improving access to family planning are likely to extend beyond the health domain by also improving well-being and contributing to the alleviation of poverty.

## Methods

### Study Approval

Human subject approvals for this study were obtained from the Harvard University Institutional Review Board (IRB; Protocol Number: IRB16-0421), the Malawi National Health Sciences Research Committee (NHSRC Approval Number: 16/7/1628), the Lilongwe District Council, the Malawi Police Service, and the Malawi Ministry of Health (MOH) to conduct the study. Memoranda of Understanding were established with our partner family planning clinic in Lilongwe, the Good Health Kauma Clinic.

### Study Setting

Our study was conducted in urban Lilongwe, the capital of Malawi. Despite declining birth rates and improvements to maternal health care, the total fertility rate or the average number of births per woman has remained relatively high in Malawi. In 2017, the average total fertility rate in Malawi was 4.2 births per woman (with a slightly lower fertility rate in Lilongwe), which is below the average total fertility rate of 4.9 births per woman in sub-Saharan Africa, but almost twice the average total fertility rate of 2.7 births per woman in South Asia and more than twice the average total fertility rate of 2.2 births per woman in Latin America and the Caribbean [16,17]. In addition, estimates from the 2015-16 Malawi Demographics and Health Survey (MDHS) show that the contraceptive prevalence rate in Malawi was 45.2% among all women of reproductive age (15-49 years) and 59.2% among married women of reproductive age. These estimated contraceptive prevalence rates are a significant increase from the 32.6% and 46.1% prevalence rates for all women and married women, respectively, from the 2010 MDHS. Nevertheless, the unmet need for family planning has remained high, with an estimated 18.7% of women in Malawi reporting to have an unmet need for spacing or limiting births [17]. Injectable contraceptives were reported to be the most popular method in Malawi in 2010 and were used by 22.5% of women, followed by intrauterine devices (IUDs) and female sterilization at 9% and 8.3%, respectively [17]. The method mix of women has not changed significantly over time among married women in Malawi, as injectable contraceptives, IUDs, and female sterilization remain to be the most popular methods among married women and are used by 30%, 11.5%, and 10.9%, respectively [17].

When compared with antenatal care, the utilization of postpartum maternal health care services remains to be low in Malawi. Although 97.6% of pregnant women received antenatal care from a skilled professional between 2012 and 2017, 57.6% of new mothers did not receive any postnatal care within the immediate postpartum period (within 48 hours following birth) [17]. Although a range of maternal health programs have attempted to combine PPFP with existing maternal health services, these programs continue to face difficulties in reaching significant portions of the population. Prior studies have shown that women in Malawi and in sub-Saharan Africa more generally face a range of barriers to accessing high-quality postpartum care, including (1) informational barriers (lack of awareness or knowledge of postpartum care options), (2) physical barriers (distance to care, long travel times to health facilities, high cost of transport, and poor access to effective transport options), and (3) barriers that impede effective service provision (long waiting times at clinics, user fees for services, lack of availability of services and supplies, and poorly trained service providers, among others) [18]. Additionally, women and children often receive postnatal care from different locations and through different providers, which often compels a woman to make the choice to seek care for her child at the expense of her own care [19]. These barriers to access are common to interventions that aim to increase access to and use of postpartum health care services, including PPFP, and are key barriers that we aimed to address when designing our intervention.

### Study Sample and Inclusion Criteria

**Figure 1:**
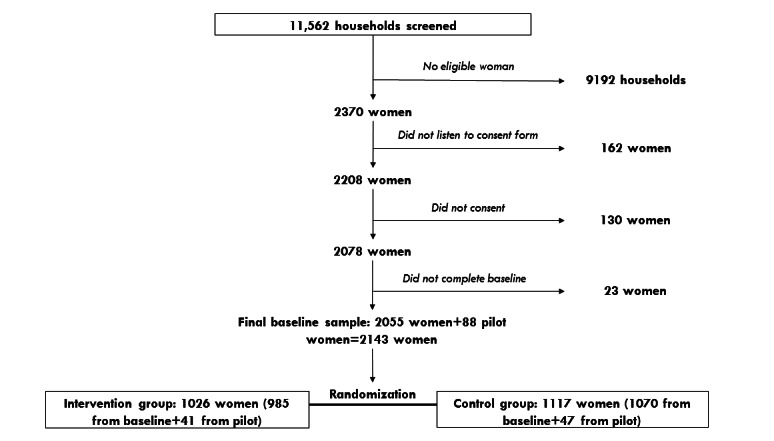
Experimental framework and flowchart.

This study is a two-armed randomized controlled trial that was conducted with a sample of women of reproductive age from urban Lilongwe, Malawi. The study consists of a baseline survey, followed by the randomization of women into intervention and control arms and the implementation of the 2-year family planning intervention. Two follow-up surveys were conducted 1 and 2 years after the baseline survey. Figure 1 outlines the general framework of the complete field experiment.

For the study, we recruited women who, at the time of the baseline survey, (1) were married, (2) were either currently pregnant or had given birth within 6 months from the time of the baseline screening, (3) were between the ages of 18 and 35 years, and (4) lived in the city of Lilongwe. Women who successfully met these criteria and consented to participate in the study were recruited. In addition, no 2 eligible women were enrolled from the same household. If multiple women from the same household were potentially eligible to be recruited based on the 4 inclusion criteria above, the youngest eligible woman from the household was chosen to participate—given that randomization was to be administered at the individual woman level, it was necessary for us to select only 1 eligible woman from a household to minimize any possible contamination across women in the intervention and control groups. We also ensured that eligible women who were selected for the study were sufficiently distant (at least five households apart) from each other, which also served to reduce any spillover effects between treated and control women who lived in the same neighborhood. Following the recruitment of women, 1 member from the recruited woman’s household was identified and selected to respond to sections in the baseline and follow-up surveys that inquired about household expenditures, assets, and consumption. The household member selected for this part of the study was required to meet the following inclusion criteria: (1) he or she was >18 years old, (2) he or she was a resident of the same household from which the woman respondent described above was selected, and (3) he or she claimed to be knowledgeable about the household’s financial status, consumption, and expenditure. The household member who successfully met these inclusion criteria and who consented to participate in this part of the study was recruited to participate. Finally, children in the household were recruited to complete an anthropometric module, including data collection of height, weight, and anemia status at baseline and again at the 2 follow-ups. The children who were selected from the household for this part of the study (1) were aged <6 years, (2) were identified as the biological or adopted children of the woman who was recruited for the main part of the study, and (3) resided in the same household as the eligible woman. Children who successfully met these inclusion criteria and whose mothers consented to them participating in this part of the study were recruited to participate.

### Recruitment and Study Timeline

Using the most recent Demographic and Health Survey (DHS) and census maps of Lilongwe’s enumeration areas and listings of households and neighborhoods, which were provided to us by the Malawi MOH; National Statistics Office (NSO); and our implementation partner, Innovations for Poverty Action (IPA) Malawi, we employed a two-stage sample selection procedure that was based on the sampling strategy used by the DHS. In the first stage, we randomly selected areas in Lilongwe to be surveyed until we selected enough areas to contain at least 11,000 households in total. In the second stage, our surveyors proceeded door-to-door to screen households in each selected area for potentially eligible women. Surveyors continued to screen households until they identified at least 2000 women for the study in accordance with the inclusion criteria listed above, and these eligible women were recruited in accordance with the recruitment protocols outlined below. Surveyors used a recruitment script to verify eligibility and presented the eligible woman with a consent form to participate in the study. Written informed consent was obtained from all participating women before proceeding to administer the baseline survey. Multimedia Appendix 1 shows recruitment script and the consent form that were administered to the women respondents. Women who met the eligibility criteria and who consented to participate in the study were recruited into the study. On the basis of our knowledge of participation refusal rates and the estimated number of eligible women in Lilongwe, we estimated the need to screen an estimated 3000 households to obtain a desired sample size of at least 2000 women. We required a study sample of at least 2000 women to achieve sufficient power to measure our outcomes of interest; see our power calculations below. As women who were selected into the study would also be at least five households apart from each other, we would need to choose enough enumeration areas to have at least 11,000 households in total (2000×5=10,000 households among the women who make up our sample and who are at least five households apart, plus an additional 1000 households that were screened but where women either did not meet the eligibility criteria or refused to participate). Recruitment from the selected enumeration areas ceased once at least 2000 women were found who met the eligibility criteria, consented to participate in the study, and were administered the baseline survey.

The planned experiment spanned 42 months. Table 1 shows the study timeline. Before recruiting and conducting the study with the main study sample, a small sample of women was recruited as a means to pilot new survey instruments and intervention activities over the study period. All research activities, including recruitment, consent, study instrument administration, and intervention administration, were administered to the pilot sample using the same study protocols as those used for the main sample. For this reason, the final analytic data sets for the study consist of data from both the pilot and main samples.

**Table 1:** Study timeline.

Study activities	Months													
	1-3	4-6	7-9	10-12	13-15	16-18	19-21	22-24	25-27	28-30	31-33	34-36	37-39	40-42
Prepare survey instrument; hire study staff and interventionists; obtain IRB^a^, partner, and local approvals; identify and finalize sampling strategy	✓	✓	✓	—^b^	—	—	—	—	—	—	—	—	—	—
Training of local staff, lay interventionists, and enumerators	—	—	—	✓	—	—	—	—	—	—	—	—	—	—
Household screening and sample recruitment	—	—	—	✓	✓	—	—	—	—	—	—	—	—	—
Baseline survey administration, randomization, and intervention administration	—	—	—	—	✓	—	—	—	—	—	—	—	—	—
Intervention period	—	—	—	—	✓	✓	✓	✓	✓	✓	✓	✓	—	—
First-year follow-up and analysis	—	—	—	—	—	—	—	—	✓	—	—	—	—	—
Intervention study close-out, second-year follow-up, and analysis	—	—	—	—	—	—	—	—	—	—	—	—	✓	—
Final reports, publications, and dissemination	—	—	—	—	—	—	—	—	—	—	—	—	✓	✓

^a^IRB: Institutional Review Board.

^b^—: indicates the month in which that specific study activity is not taking place (eg, the preparation of the survey instruments only take place in the first 9 months and is completed by then).

### Informed Consent and Participant Privacy

The process to obtain consent was consistent for all potential participants: women, financially knowledgeable members of the household, and children <6 years. Written consent to participate was obtained from all participants before administering each survey (baseline and annual follow-ups). Once participants agreed to join the study, a copy of the consent form script was read to them and they were given opportunities to ask questions and express concerns. Surveyors checked for comprehension throughout the consent process. This process was estimated to take between 5 and 10 min, although it took longer if a participant had many questions—refer to the consent documents in Multimedia Appendix 1 for details concerning the consent process. After completing the consent script, potential participants were encouraged to ask questions and asked if they would like to participate. If the participant wished to take further time to reflect, the surveyor and the participant determined the time and method of reconnecting. If the potential participant agreed to be a part of the study, consent was obtained and documented by obtaining the signatures of both the participant and the study staff member who conducted the consent discussion. Upon consenting, the surveyor then conducted the survey.

For follow-up surveys, verbal consent to participate was obtained over the phone from only those participants who were originally recruited at baseline but since either moved outside of Lilongwe or were unreachable in person after 3 contact attempts. The only respondents who were recontacted for the phone follow-up survey were those respondents who were simply lost to follow-up. Respondents who had previously indicated that they were no longer interested in participating in the study were not contacted.

Throughout the consent process, the surveyor clearly explained to the participant that even if she decided to participate and sign the consent form, she could decide at any time to end her participation. If the study participant was not literate, then a witness who did not work for the study signed the consent form. In the absence of witnesses, the participant could confirm their consent by placing a thumbprint on their consent form and a photo of the thumbprint was taken as a record of consent. Moreover, participants were encouraged to contact the researchers with any further questions during the informed consent discussion or any time during the study. Consent was obtained before any surveys were conducted. All participants were informed that their participation was completely voluntary and that they could choose not to participate in the study or to end their participation in the study at any time.

The recruitment scripts, consent forms, and survey instruments were translated from English into Chichewa by a certified translator.

With regard to consent during the administration of the intervention, women were informed that they could take part in any component of the interventions that was offered to them; they could take up and stop any or all components of the intervention at any time. For example, counselors asked for a woman’s consent to participate in a counseling session each time they visited a woman’s home.

All surveys and administration of the intervention were conducted in a private room. To maintain a respondent’s privacy during an attempt to reach her by phone, the field enumerator conducting the call left no indication of the reason for the phone attempt (eg, voicemail, text message) on the respondent’s phone should there be no response to a call. Any disruption or interruption during the interview, either in person or by phone, resulted in the postponement or termination of the interview.

### Randomization

Following the baseline survey, women who consented to participate in the study were individually randomized into 1 of 2 experimental arms: an intervention arm or a control arm. The women were randomized to the intervention and control groups such that intervention assignment was balanced according to the following baseline characteristics: neighborhood or household cluster, distance to the nearest family planning clinic, number of living children, months since last live birth, current use of family planning, age of marriage, educational attainment, and household wealth. As part of the balancing process, strata by each combination of characteristic values were created and observations were assigned to their respective strata. Observations within each stratum were then individually randomized by the Principal Investigators in a 1:1 allocation to either the treatment or the control group using computer-generated randomization through STATA, version 13 (StataCorp). Block randomization was not used in this study. Following individual randomization, the IPA Malawi intervention team implemented the intervention to the participants who were randomized into the intervention arm.

### The Intervention

Following randomization of women into the intervention and control arms, women assigned to the intervention arm were offered the following 3 intervention components over a 2-year period:

1. Transportation component: women were offered a free transportation service from their homes to our partner family planning clinic, the Good Health Kauma Clinic. The transportation service was provided by a driver who was hired and trained by our local implementation partner, IPA Malawi. Women received the driver’s phone number and were instructed to contact the driver to transport them to the Good Health Kauma Clinic during the clinic’s normal working hours, which are between 8 AM and 5 PM from Monday to Saturday. The driver maintained a daily schedule of the women who requested his services, and women were instructed to notify the driver at least one day before they wished to go to the clinic to make sure that the driver was able to transport them. The driver also provided 1 day’s notice to the Good Health Kauma Clinic to inform them of how many women from the study could be expected to attend the clinic on the following day. The Good Health Kauma Clinic assured the project team that women in the intervention arm who come for services would not have to wait more than 1 hour before being examined by a medical professional. In addition, one of our female field managers from IPA Malawi accompanied the driver at all times. Although all women in the intervention arm were presented with pictures of the field team (and could therefore recognize our team members), the presence of another woman in the vehicle served to minimize potential stigma associated with a woman traveling alone in the company of another man.
2. Counseling component: women who were assigned to the intervention arm were also offered free, private family planning counseling sessions over the 2-year intervention period. The counseling sessions were provided by trained counselors and included a risk assessment for clinical methods and detailed information on methods switching; side effects associated with each method; and the benefits of contraception, birth spacing, and dual protection. Consultations were designed to promote informed choice by discussing common misperceptions surrounding family planning and the use of modern contraceptives. Women received a detailed information brochure on birth spacing and side effects and also received counseling on both modern and natural family planning methods, in Multimedia Appendix 2. Strategies on how to communicate family planning messages with partners and on how to increase partner awareness were conveyed during sessions. Counseling sessions were scheduled to last no more than 1 hour per session and were administered in a private room by a counselor who was trained to provide family planning and reproductive health services. Counselors were hired and trained by IPA Malawi. We enlisted the support of the Malawi Reproductive Health Directorate (RHD) and several international nongovernmental organizations who work on family planning, including Population Services International (PSI), Banja La Mtsogolo (BLM), the Family Planning Association of Malawi (FPAM), and FHI360, to help us develop training materials, brochures and flyers, and other counseling resources. We also collaborated with the Malawi RHD, BLM, and PSI to assist with the counselor training. Women in the intervention arm received a total of 6 counseling sessions, 1 comprehensive 90-min session just after administration of the baseline (within 1 month) and 5 shorter 45-min follow-up sessions that were spaced out over the 2-year intervention period. The first session introduced women to the range of available family planning methods and counseled women on side effects. In the first session, counselors also informed women in the intervention arm about the transport service (described above) and side effects management service (described below) that were available to them and provided women with the necessary information on how to access these services. Counselors also provided their phone numbers to women and were on call over the course of the study period to respond to any questions and concerns.
3. Financial reimbursement component: women who were assigned to the intervention arm were financially reimbursed for any out-of-pocket expenditures that they incurred for receiving family planning care at the Good Health Kauma Clinic. Costs that were reimbursed at the Good Health Kauma Clinic included costs related to the procurement of family planning medications and contraceptive methods, family planning consultation fees, lab test fees, and exam fees. The reimbursement allowance for each woman was 17,500 MKW (US $25.00) and could be redeemed by the woman over multiple visits at the Good Health Kauma Clinic over the 2-year intervention period. For every family planning service that the woman received, the cost of the service was deducted from her reimbursement allowance of 17,500 MKW.

In addition, women who were assigned to the intervention arm and who experienced any side effects because of contraceptive use over the course of the 2-year intervention period received a series of services for the treatment of side effects. In the event that a woman in the intervention arm experienced a side effect or contraindication, she could contact a trained Obstetrician-Gynecologist at the Kamuzu College of Medicine in Lilongwe, via telephone, and would receive advice on how she can best seek care. The doctor would conduct a preliminary telephone consultation and would refer the woman over the phone to seek care at their nearest public clinic, public hospital, or the Good Health Kauma Clinic. All women in the intervention arm also received an *emergency package* during the first counseling visit from the counselor (see above). This *emergency package* consisted of (1) a transport voucher, equivalent to an estimated 6500 MWK (US $9.28) and (2) a mobile phone credit scratch card for the mobile provider of their choice, equivalent to 500 MWK (US $0.72). A template of the transport voucher is provided in Multimedia Appendix 3. This *emergency package* was given to all women in the intervention arm, regardless of whether they took up any intervention component or experienced a side effect. The counselor informed the woman that, in addition to the other side effects management services mentioned above, the woman could use the *emergency package* that she was given to cover (1) any phone airtime costs that she used to have a consultation with one of the doctors who are on call and (2) any emergency transport costs (taxi) she incurred to travel to a health facility where she can receive treatment for her contraceptive-related side effects. The transport voucher could be presented to any taxi driver in the city of Lilongwe and the taxi driver would, in turn, redeem the voucher at the IPA Malawi office in exchange for cash equivalent to the cost of the trip. The woman was asked to keep receipts of any costs she incurred at the health facility so that she could be reimbursed later. Costs for which the woman could be reimbursed included costs of medications and lab tests, costs of additional consultations at the health facility, and costs of switching or discontinuing methods. The maximum reimbursement amount that a woman was eligible to receive for the treatment of family planning-related side effects or contraindications is 35,000 MWK (US $50.00) over the 2-year intervention period. The reimbursement could be applied to cover the cost of treatment for side effects for all family planning methods used by the woman, regardless of where the method or treatment was procured. All reimbursements for incurred costs were distributed as closely as possible to the time that the reimbursable cost was incurred.

Following the randomization of women into the intervention and control arms, women who were assigned to the intervention arm were visited by the family planning counselor. During this initial visit, the counselor described all 3 intervention components, including their terms and conditions, to the woman. The counselor answered any questions that the woman may have had about the intervention and then asked for and confirmed the woman’s consent to participate in the intervention using a Terms of Service document (available upon request). Each woman received a paper copy of the Terms of Service document. If the woman consented to participate, the counselor then administered the first counseling session. Women assigned to the intervention arm could withdraw their participation from any intervention activity at any time and could also rejoin at any time without any penalty over the 2-year intervention period.

### Control Arm

Women who are assigned to the control arm received a package of publicly available literature and information on the benefits of family planning as well as information about their nearest family planning clinic. This information package was delivered to all women at the time of the baseline interview. Women in the control arm were only recontacted by the research team at follow-up.

### Participant Compensation

All women who participated in the study received a small token of appreciation (3 bars of soap, a monetary equivalent of 500 MWK [US $0.66]) after completing each survey. Parents or guardians of children who participated in the anthropometric measurement portion of the study received a small bag of puffed rice cereal (a monetary equivalent of 100 MWK [US $0.13]) after completing each survey. To avoid coercion into participation, participants were not informed of these tokens until after the survey had been administered.

All women who participated in the phone follow-up survey received mobile phone credit in the amount of 500 MWK (US $0.66) by means of a mobile airtime transfer. To avoid coercion into participation, women were not informed of this airtime transfer until after the survey had been administered.

### Primary Outcomes

At the designated 1-year and 2-year follow-up periods, the entire study sample of women was resurveyed to create a panel of individual women in which each woman and household would be observed over 3 time periods. In each follow-up round, we collected survey data on a range of primary outcomes of interest, which include (1) contraceptive use, including changes in contraceptive prevalence, changes in method mix, and adherence to methods (compliance and discontinuation) and (2) fertility and birth spacing outcomes, including pregnancy status, parity, and time since the last birth.

### Secondary Outcomes

A range of secondary outcomes were collected in each survey wave, including (1) child anthropometric outcomes, including child height, weight, and anemia status for all children born after the start of the intervention; (2) sexual and marital well-being; (3) women’s anthropometric outcomes, including height, weight, and anemia status; (4) women’s and children’s educational attainment, including time spent in school, type of school (public or private) attended, and the highest educational qualification achieved; (5) work, income, and women’s employment, including women’s time use (time spent on child care vs household and income-generating activities) and sources of household income; (6) household assets and wealth, including changes in asset ownership over time; and (7) expenditures, in particular changes in food expenditures and durable expenditures over time.

Outcomes were collected according to the schedule outlined in Table 2. All survey instruments (baseline and follow-up) and intervention-monitoring tools to track participants and intervention uptake over the course of the study are available upon request.

**Table 2:** Outcome measures and instruments.

Outcomes and instruments	Baseline	Follow-up, year 1	Follow-up, year 2
Attitude and knowledge of family planning	✓^a^	✓	✓
Contraceptive use	✓	✓	✓
Pregnancy and fertility outcomes	✓	✓	✓
Child anthropometry (height, weight, and anemia)	—^b^	✓	✓
Women’s anthropometry (height, weight, and anemia)	✓	✓	✓
Educational attainment	✓	✓	✓
Formal and informal employment	✓	✓	✓
Women’s time use (primary and secondary activities)	✓	✓	✓
Household income and expenditure	✓	✓	✓
Household assets	✓	✓	✓
Baseline survey	✓	—	—
Year 1 follow-up survey	—	✓	—
Year 2 follow-up survey	—	—	✓

^a^✓: indicates that data on that outcome were collected in the indicated survey wave.

^b^–: indicates that data on that outcome were not collected in the indicated survey wave.

### Survey Instruments and Monitoring Tools

A paper version of the baseline survey instrument, which served as a template for all follow-up surveys, is provided in Multimedia Appendix 1. Before administering the survey, we explained the purpose of the study to respondents and asked for their consent using the study consent forms. Baseline surveys, anthropometric measurements, and related study activities (eg, discussion of anthropometric and anemia test results) were administered in a private room in the woman’s place of residence and lasted approximately two hours overall (75 min for administering the child anthropometry and woman questionnaire to the woman and her children aged <6 years and 45 min for administering the household questionnaire to the financially knowledgeable respondent). Short breaks of 5 to 10 min were given to the respondents at the end of each section, and additional breaks were taken at the request of the participant and at other scheduled times (eg, mealtime, picking children up from school, etc) as needed. Surveys were conducted in Chichewa, the local language, and followed the format of the survey questionnaire, which were electronically programmed into Android tablets. To successfully track our participants over time, we collected identifiable data, including names and contact information for each of the survey respondents (the woman, her children aged <6 years, and the financially knowledgeable member of the household), the names and background characteristics of other household members, the household address, contact phone numbers and emails, and GPS coordinates of the household. For the purpose of minimizing loss to follow-up, we took photographs of the household and of the survey respondents, and we asked respondents to provide the names and contact information of 2 contacts who did not live in the household and who could be contacted if the respondents could not be directly located in the follow-up period. To protect participant privacy and ensure confidentiality, all identifiable data are appropriately stored and secured in accordance with the data security measures described in the sections below.

The 1-year and 2-year follow-up surveys were administered in person to all women who continued to live in urban Lilongwe and who could be contacted within 3 contact attempts by the local field team. For women who either moved out of urban Lilongwe or who were unreachable after 3 contact attempts, the field team attempted to contact them by phone up to a maximum of 3 attempts; women who were reachable by phone within these 3 attempts were asked to participate in a short phone follow-up survey. To maintain a respondent’s privacy during an attempt to reach her by phone, the field enumerator conducting the call left no indication of the reason for the phone attempt (eg, voicemail, text message) on the respondent’s phone if there was no response to a call. To ensure that the respondent’s participation remained private, the field enumerator only continued with the phone call once she received assurance from the woman that she was able to speak on the phone without being overheard or interrupted. Any disruption or interruption during the phone call resulted in postponement or termination of the call. The phone follow-up survey instrument took no more than 20 min overall, and was an abbreviated version of the main in-person follow-up survey instrument. The aim of the phone survey was to maximize survey follow-up rates by attempting to include women who were physically unreachable. The only respondents who were recontacted for the phone follow-up survey were those respondents who were simply lost to follow-up. Respondents who had previously indicated that they were no longer interested in participating in the study were contacted.

The phone follow-up survey instrument consisted of abbreviated modules from the main in-person follow-up survey instrument. Before administering the phone survey, we explained clearly the purpose of the follow-up to respondents and asked for their consent to participate in the survey verbally over the phone. Following receipt of consent, we ensured confidentiality and privacy of responses by asking the respondent to find a private room or space where their responses could not be overheard by others. We proceeded with the phone survey only after having received confirmation from the respondent that her responses could not be overheard.

### Analysis Plan

Analysis of quantitative study data will be conducted using STATA and R (the R Project), where appropriate. A descriptive analysis will be performed for all variables, and unadjusted comparisons between experimental arms will be conducted. Descriptive statistics will be performed, including frequencies, means, and standard deviations. In addition, chi-square tests and one-tailed *t* tests will be used to examine associations in the data. A *P* value <.05 will be considered statistically significant for all statistical tests that are conducted. Given our hypotheses of the impact of our intervention on our key outcomes, one-sided hypotheses tests will be conducted for all our main analyses. Continuous variables will be tested for normality and nonnormal values will be categorized or transformed appropriately.

Our main econometric specifications will estimate the intent-to-treat (ITT) effect of our family planning intervention on fertility and other outcomes by directly regressing our outcomes of interest on a binary variable indicating receipt of the intervention. The main econometric specification for estimating the ITT effect of our family planning intervention is defined as follows:

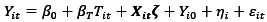



where *Y_it_* is the outcome variable of interest for woman *i* in the period *t*=0,1,2 for baseline, 1-year follow-up, and 2-year follow-up, respectively; *T_it_* is an indicator of assignment to the treatment arm; *X_it_* is a vector of individual-level covariates that are controlled for in the analysis; *_i_* is the individual-specific fixed effect; *Y_i0_* is the baseline level of the outcome; and *ε_it_* is the error term. Here, the outcome variables of interest include immediate, intermediate, and long-term outcomes mentioned in the previous sections.

We will conduct several subgroup analyses to examine how the family planning intervention effects vary across particular subpopulations. Subgroups of interest include pregnant women, new mothers, women who have previously used family planning, women who expressed a desire to space or limit births at baseline, poorer women, and women with low educational attainment. In addition, we will estimate heterogeneous treatment effects for girls, older children, and high-parity households.

### Sample Size and Power Calculations

The sample size of this study was powered to primarily identify the effect of using family planning on fertility; however, we will also examine the effects of the intervention on other maternal and child health outcomes of interest, in addition to key social and economic measures of well-being. On the basis of the preliminary power calculations for our primary outcomes of interest, a total of 2000 women were needed to be enrolled in the study. Of the women who were enrolled, the family planning intervention, baseline survey, and 2 follow-up surveys were administered to 1000 women (the treatment group), whereas a basic family planning information package, the baseline survey, and 2 follow-up surveys were administered to the other 1000 women (the control group).

Our target baseline sample consists of 2000 women who met the eligibility criteria and who consented to participate in the study. Previous research studies in Accra, Ghana, have found that 32% of initially screened women either did not meet the eligibility criteria or refused to participate [20-22]. Therefore, to meet our target sample size of 2000, we would need to screen 3000 households if we conservatively assumed a combined ineligibility or refusal rate of 32% from the screening. Of the 2000 women who were to be recruited into the study at baseline following the initial screening process, 1000 women were to be randomly assigned to the intervention arm and the remaining 1000 women were to be randomly assigned to the control arm. On the basis of prior study findings from Zambia [13], we expected an attrition rate of 27% in the sample over a 2-year study period, which would leave us with an attrition-adjusted sample size of 730 women in each treatment arm, or 1460 women overall, at the end of the 2-year study period.

Given this attrition-adjusted sample size, we have powered our study to detect effects in 2 key outcomes, namely changes in contraceptive prevalence and fertility, the latter measured by the number of births per woman.

### Contraceptive Prevalence

Using modern contraceptive prevalence estimates for Lilongwe from the 2010 Malawi DHS, we expect a modern contraceptive prevalence rate of 19.5% among women aged 18 to 35 years who are either currently pregnant or who are up to 6 months postpartum at baseline. To infer a potential effect size for our intervention, we look to evidence from (1) the Navrongo study in Ghana, which found that a family planning intervention with a comprehensive outreach and contraceptive delivery component increased contraceptive use by 6 to 8 percentage points over a 4-year study period and (2) the Matlab study in Bangladesh, which found far larger effects of contraceptive uptake among women in the intervention areas over a longer study period [9,23]. Our power calculations show that we will have 90% power to detect a 6.5 percentage point increase in the modern contraceptive prevalence rate in the intervention arm, from 19.5% to 26%, assuming that we will have an attrition-adjusted sample of 1460 women (730 women in each arm) at the end line.

Table 3 presents the levels of power 1-ß that will be achieved for various minimum effect sizes for modern contraceptive prevalence use (ie, the difference in the modern contraceptive prevalence rate between the intervention and control arms at end line), assuming a baseline contraceptive prevalence rate of 19.5 percentage points in both intervention and control arms and a fixed end-line sample size of 1460 women (730 in each arm).

**Table 3:** Power calculations—contraceptive prevalence rate.

Control CPR^a^, n (%)	Intervention CPR, n (%)	Significance level, α	Control sample size	Intervention sample size	Power
142 (19.5)	190 (26)	.05	730	730	0.90
142 (19.5)	183 (25)	.05	730	730	0.79
142 (19.5)	175 (24)	.05	730	730	0.65
142 (19.5)	168 (23)	.05	730	730	0.47

^a^CPR: contraceptive prevalence rate.

### Fertility (Number of Children Per Woman)

Similarly, we use fertility estimates for Lilongwe from the 2010 Malawi DHS and assume a baseline fertility rate of 4.5 births per woman in the sample at baseline. To infer a potential effect size, we again look to evidence from the Navrongo and Matlab studies, both of which found a total reduction of 1 birth per woman (equivalent to a 15% decrease in their respective baseline fertility) among women in their respective intervention arms over their respective study periods [9,23]. In recognizing that both the Matlab and Navrongo studies were larger-scale, cluster-randomized designs with longer follow-up periods, we estimate that we will only be able to observe differences of 0.5 births. Our power calculations show that we will have 99% power to detect a 0.5 birth per woman decrease, equivalent to a 12% decrease in fertility, in the intervention arm from 4.5 children per woman to 4.0 children per woman, assuming an attrition-adjusted sample of 1460 women (730 women in each arm) by end line.

Table 4 presents the levels of power 1-ß that will be achieved for various minimum effect sizes for fertility (ie, the difference in the average number of children per woman between the intervention and control arms at end line), assuming a baseline fertility of 4.5 children per woman in both intervention and control arms, a standard deviation of 2 children per woman for both arms, and a fixed end-line sample size of 1460 women (730 in each arm).

Finally, robustness checks (5% and 10% sample truncations and coarsening of independent variables) and falsification tests, which include placebo regression, simulation, and resampling methods, will be conducted to ascertain the strength and significance of our estimates. We will also conduct attrition-adjusted analyses to assess the extent of differential loss-to-follow-up outcomes.

**Table 4:** Power calculations—fertility.

Control fertility, number of children per woman	Intervention fertility, number of children per woman	Significance level, α	Control sample size	Intervention sample size	Power
4.5	3.5	.05	730	730	1
4.5	4.0	.05	730	730	0.99
4.5	4.2	.05	730	730	0.89
4.5	4.3	.05	730	730	0.61

### Dissemination Plan

For this study, we have partnered with Dimagi, a privately held social enterprise based in Cambridge, Massachusetts, and will use Dimagi’s CommCare open-source software suite to develop our electronic survey instruments, supervise survey enumerators and field staff activities, collect and manage respondent data, and monitor data quality. In addition to supporting our study from their Cambridge headquarters, a Dimagi field engineer will provide on-site technical support and train local field staff and survey enumerators to use the CommCare software for administering the baseline survey. We have also formed a partnership with IPA Malawi, a US-based nonprofit research organization with operations in 42 different countries. IPA Malawi has provided extensive technical and research assistance in health and development to many governmental and nongovernmental organizations in Malawi, including the MOH and the World Bank. We worked with the IPA Malawi management team and a hired team of surveyors, field managers, intervention staff (family planning counselors and driver), and other support staff to conduct the fieldwork. IPA Malawi’s primary role in the study was to conduct the local field research activities, including (1) hiring, training, and management of the local field staff; (2) data collection, monitoring, and evaluation; (3) implementation of the intervention; and (4) assisting the investigators with the dissemination of results in Malawi. Finally, we worked closely with the MOH and RHD on dissemination activities for this study and regularly met with representatives from the MOH and RHD to share results and to facilitate collaboration on the study.

Aggregate summary statistics and final peer-reviewed publications will be shared with participants, key partners (IPA Malawi, Dimagi, Good Health Kauma Clinic), the Malawi MOH, the Malawi RHD, and the Malawi NSO. Individual survey responses of other participants will not be shared among the participants verbally, by recording, or in writing. Each interviewee’s responses will remain confidential as per the terms of their consent to study participation.

We will produce the output in peer-reviewed journals, working papers, and policy briefs that are accessible to academics, policy makers, and practitioners and contribute to the policy change in this area. Aggregate results and final publications will be disseminated to the community and local institutions where the research is conducted. The research team will also present intervention findings at local and national venues, including annual meetings of professional organizations, community gatherings, and meetings with local service providers. Our work will also be effectively disseminated to practitioners through local partnerships as well as through the RHD and MOH. We have worked with these organizations during the study design phase to ensure that the interventions are appropriate for the country setting. Upon completion of the intervention, we will know the midterm and long-term effects of the interventions, and our local partners can use this information to expand or tailor their services to help women achieve their family planning goals within the specific country context. We will also share our descriptive and analytical findings with members of the community who are engaged in advocacy efforts.

Our dissemination efforts are engrained in our interventions from the outset. We tailored our information packs and counseling materials for women in Malawi based on knowledge gained from preliminary studies of the family planning environment. This dissemination of information as part of the intervention will help us learn how to disseminate the results of our research to the women in the communities we are working in.

### Data Confidentiality

All identifiable data collected from surveys (both baseline and follow-up) and from the intervention were administered in an electronic computer-assisted personal interview format using the Dimagi CommCare survey management system. Electronic survey data were collected by interviewers on Android-based tablets, and data were securely transferred onto a CommCare-supported secure cloud server. The CommCare cloud server was Health Insurance Portability and Accountability Act–compliant and met all the necessary security requirements for storing identifiable data. A technical overview of the CommCare system and an electronic version of the CommCare Terms of Use or End User License Agreement can be found on the web [24,25].

All data uploaded to the CommCare server were encrypted and password protected in accordance with the approved data storage regulations. For each collected data case, all personal identifiable data were separated from the other nonidentifiable data. Identified data were only accessed for the purpose of revisiting the households at the 1- and 2-year follow-up periods. Only deidentified data sets will remain available for analysis purposes after the end of the study. All confidential identifiable data were secured by trained study personnel upon collection. Identifiable hardcopy data, including signed consent forms, were stored in locked cabinets in access-limited rooms at the IPA Malawi office.

With specific regard to the dissemination of identifiable data, policies were put in place to limit data dissemination beyond the immediate research team (comprising the principal investigator and coinvestigators and the project managers). Team members received training on the proper handling and storage of such data, and all staff members of the study were required to sign a data confidentiality agreement. Data sharing of deidentified data between the principal investigators and authorized research staff was conducted in person. Deidentified data are transmitted from Malawi to the Harvard T. H. Chan School of Public Health (HSPH) via secure file transfer (through the Harvard system). All hardcopy data and electronic data are retained for 7 years after study closure, after which they will be destroyed (shredding hardcopies and permanently deleting all electronic files). Following the completion of the analysis, data will be stored on the HSPH network and will be deleted from all the hard drives of all the authors.

### Data Safety and Monitoring

The study principal investigator, DC, has assumed overall responsibility for the safety, monitoring, and review of the data. DC and MK have been present during the rollout of the baseline survey, intervention, and follow-up surveys. The local project manager at IPA Malawi; the local coinvestigator, Bagrey Ngwira; and the principal investigator, DC, have reviewed any adverse events. This information has been provided to the IRB at Harvard University and the Malawi NHSRC ethics committee in Lilongwe. Unanticipated adverse events were immediately reported by the local field team to both the Harvard IRB and the NHSRC in writing within 5 business days. We did not anticipate that there would be any research-related injuries. Nevertheless, IPA Malawi field staff (surveyors, field managers, and interventionists) were trained in first aid and project managers at IPA Malawi were on call via mobile phone during the entire duration of the study.

### Regulatory Compliance

MK, Bagrey Ngwira, and the IPA Malawi project manager are the immediate supervisors of study staff in the field (research assistants). In addition, the local team and the Boston-based team have been in regular contact via email in the interim to discuss the progress of the study protocol procedures. DC and MK traveled regularly to Malawi over the course of the study period, particularly during the data collection phases to monitor field activities. All regulatory documentation will be maintained for 7 years after IRB study closure.

### Authorship Eligibility Guidelines

A publication committee consisting of Dr DC, the overall principal investigator, and Dr MK has been established to address and decide on all matters related to access to project data and publications using such data. All guidelines for data access and publications are outlined in the publication committee Terms of Reference document (available upon request).

### Availability of Data and Materials

Following our own use and analysis of the data and publication of the main findings that have been identified as part of the study preanalysis plan (a minimum period of 1 year from the end of the study), we hope to open access to deidentified baseline and follow-up survey data at no cost to authorized users. Only deidentified data will be available for download through a secure website, through which authorized users can download deidentified survey data files for legitimate academic research. To access the data, prospective users must first register on the secure website and must then create a new research project request. The request must include a project title and a description of the analysis that the user proposes to perform with the data. The requested data should only be used for research or study purposes. To request the same data for another purpose, a new research project request needs to be submitted. Requests for data access will then be reviewed by the principal investigator, who can then grant or deny access to the user. All publications that users produce from the data set must appropriately acknowledge the data source and project from which the data were collected. Once downloaded, the data sets must not be passed on to other researchers without the written consent of the principal investigator. All reports and publications based on the requested data must be sent via email to the principal investigator in a PDF file or as a printed hardcopy. See the Publications Committee’s Terms of Reference document (available upon request).

## Results

### Recruitment, Study Sample, and Randomization

Field activities for the baseline survey began with field staff hires, training, and piloting of the survey instrument in July and continued through August 2016. During the 5-month baseline survey period, 11,562 households were approached and women in these households were screened based on the eligibility criteria. On the basis of the eligibility screening, 2370 women (20.5%) in these households were eligible to participate in the study. Of these 2370 women, 2208 women (93.1%) agreed to go through the consent form with the enumerator, and 2078 women (94.1%) of the 2208 women who agreed to go through the consent form consented to participate and were subsequently enrolled in the study. This consenting sample of 2078 women constituted 87.7% of the eligible sample. Of these 2078 women, 2055 women (98.8%) completed the baseline survey and were eligible to be randomized into the intervention or control groups. From this baseline sample, 985 women were randomly assigned to the intervention group whereas the remaining 1070 women were randomly assigned to the control group. In addition to the 2055 women who were selected for the main study, 88 women were interviewed as part of a preliminary pilot study to test the feasibility of the survey instruments and implementation of the intervention. As part of the intervention rollout, these 88 respondents were also randomized into the treatment (n=41) and control (n=47) groups. The final analytic sample for the baseline survey comprised 2143 eligible women, of whom 1026 women were randomized into the treatment group and 1117 women were randomized into the control group. The experimental framework is presented in Figure 1.

### Intervention Activities

Rollout of the multicomponent family planning intervention to women assigned to the intervention group began shortly after the launch of the baseline survey in September 2016. Six family planning counselors (registered nurses and midwives with previous counseling experience in family planning) were identified in mid-September 2016 and were trained through October 2016 to administer 6 counseling sessions in women’s homes over a 2-year intervention period. Counseling of clients in the intervention group began in November 2016 and concluded in March 2018, at which time counselors may have completed up to 6 visits with each client.

In addition to hiring 6 counselors, the study management team hired and trained a licensed taxi driver in October 2016 to assist with the implementation of the transportation component of the intervention. In October 2016, the management team also identified an obstetrician at the Kamuzu College of Medicine to be the *medical doctor on call*. The obstetrician was asked to be responsible for (1) answering any calls from clients, (2) providing any support or consultation services over the phone, to the best of his ability, and (3) referring any clients who may be experiencing health concerns, particularly those related to their use of family planning, to the management team for follow-up.

Counseling activities with women in the intervention group concluded in March 2018; however, other intervention activities (providing transportation to women to visit the Kauma Clinic for services, providing financial reimbursements to women for any family planning services that they obtain) continued until February 2019.

### Follow-Up Surveys

Two follow-up surveys were conducted 1 and 2 years after the baseline survey. Field activities for the Year 1 follow-up survey (wave 2) began with field staff hires, training, and piloting of the follow-up survey instrument in July 2017 and continued through August 2017. Official data collection for the baseline survey began in August 2017, and the last respondents were interviewed at the end of February 2018. During the 6-month Year 1 follow-up survey period, a total of 2092 women (which includes the full sample of 2055 women from the main study and an additional 88 women who were interviewed at baseline as part of the pilot phase of the study, but did not include the 51 women who withdrew from the study before start of the Year 1 follow-up survey) were selected for follow-up at their homes. A total of 1773 women, or 82.73% of women who were eligible for follow-up, were successfully contacted and reinterviewed at follow-up. Analyses of the 319 women who were lost to follow-up for this survey were conducted.

Field activities for the Year 2 follow-up survey (wave 3) began with field staff hires, training, and piloting of the follow-up survey instrument in July 2018 and continued through August 2018. Official data collection for the survey began in August 2018, and the last respondents were interviewed at the end of February 2019. During the 6-month Year 2 follow-up survey period, a total of 2090 women, which includes the full sample of 2055 women from the main study and an additional 88 women from the pilot sample but excludes 51 women who were withdrawn from the study before the start of the Year 2 follow-up survey and 2 women who had died from causes unrelated to the study, were selected for follow-up at their homes. A total of 1669 women, or 77.88% of women who were eligible for follow-up, were successfully contacted and reinterviewed at follow-up. Analyses of the 421 women who were lost to follow-up are being conducted.

### Analysis

All study-related field activities for the Year 2 follow-up survey concluded in March 2019. Cleaning and analysis of the baseline, Year 1, and Year 2 data is ongoing and will continue through 2021. A complete cleaned survey wave includes the following: a recoded and indexed data set, a data codebook, a recode map and variable list, a final survey questionnaire, a final report and user manual for the survey wave, and data analysis files and templates. Analyses of the primary outcomes are ongoing and are expected to be completed by 2021.

## Discussion

### Study Progress

We conducted a randomized controlled trial to identify the causal impact of improved access to PPFP among 2143 married postpartum women from urban Malawi. Women who were assigned to the treatment arm (n=1026) received a 2-year–long multicomponent family planning intervention that consisted of (1) a brochure and up to 6 home visits from trained family planning counselors, (2) free transportation to a high-quality family planning clinic, and (3) financial reimbursement for family planning services, consultations, and referrals for services. Two follow-up surveys were conducted 1 and 2 years after the baseline survey with 1773 and 1669 women, respectively. Through this randomized controlled trial, we aimed to investigate the extent to which improvements in access to family planning have effects on first-stage outcomes (contraceptive use, method mix, and adoption of long-acting methods, among others) as well as key intermediate and longer-term outcomes (short birth intervals, likelihood of pregnancy at 2 years, and downstream measures of well-being) of interest.

Although there is growing evidence to support the potential effectiveness of improved access to PPFP services on contraceptive use and method mix within the first year after birth, a recent review of PPFP interventions in low- and middle-income countries found that there is little to no evidence of the impact of such interventions on longer-term measures of contraceptive continuation, birth spacing, or pregnancy risk beyond the first postpartum year [1]. As a result, little is known about the role of PPFP on the continuation of contraceptive use and on birth spacing. In conducting this trial, we aim to provide the first causal estimate of the impact of PPFP on birth spacing and risk of subsequent pregnancy in sub-Saharan Africa, and perhaps globally.

Our PPFP intervention was designed to address the key barriers that women in urban Malawi face when seeking and accessing reproductive health services. When considering family planning within the larger context of maternal and reproductive health, we identified both barriers to access that are particular to family planning care-seeking behavior and utilization in addition to more common barriers to access (eg, geographic barriers, financial accessibility constraints, etc) [26]. Fear of contraceptive-related side effects has been identified as one of the most commonly cited barriers to family planning utilization and continuation, and is consequently a key contributing factor to unmet need for family planning, particularly in Malawi, where hormonal methods of contraception such as injectable contraceptives (Depo-Provera) are the most widely used [14,15,27]. In recognizing the role of contraceptive-related side effects on uptake and continuation, we included family planning counseling sessions that specifically focus on informing women in the treatment group about side effects and aimed at addressing myths and misperceptions around contraception. In addition, we provided women in the intervention arm with access to free over-the-phone consultations with a doctor, a service that women may utilize in the event that they may experience contraceptive-related side effects.

Our key outcomes are measured using a range of validated metrics and instruments. The baseline survey instrument consists of modules from the household and women’s questionnaires from the Malawi DHS, which is a nationally representative survey that includes information on marriage, fertility, family planning, reproductive health, and child health [2] and modules on employment, household expenditures, and time use from the World Bank’s Living Standards Measurement Study and the Institute of Statistical, Social and Economic Research Ghana Time Use and Health Study. By adapting these instruments for our study, we will also be able to compare our findings across a range of nationally representative data sources. When assessing the impact of our intervention, it will be important for us to reconcile our findings with previous evidence, particularly from the Matlab and Navrongo studies, where much of the evidence of family planning programs is generated. By the same token, we have also gathered costing data to speak to the cost-effectiveness of our intervention relative to other similar PPFP and reproductive health programs that have been implemented and evaluated.

### Conclusions

There is an increasing emphasis by policy makers and practitioners on evidence-based policy for family planning and reproductive health. Practitioners require a strong evidence base of what works in family planning to decide which interventions are most likely to achieve the desired outcomes. Given the many competing needs for funds in developing countries, high-quality evidence that demonstrates the benefits and effectiveness of family planning is required if policy makers are to justify the provision of family planning services. Through this study, we seek to fill the current knowledge gaps on the effectiveness of family planning interventions by directly identifying the impact of an increase in access to family planning on fertility and health outcomes in a sub-Saharan African context, where rigorous experimental evidence is scarce. Service providers and policy makers will be able to use the findings from our study to improve the availability of and access to reproductive health services in Malawi as well as in other similar settings.

### Related Articles

At present, no other publication containing the results of this study has been published or submitted to any journal.

## Appendix

Multimedia Appendix 1 Survey instrument, recruitment script, and consent form.

Multimedia Appendix 2 Family planning brochure.

Multimedia Appendix 3 Transport voucher.

## Abbreviations

BLM: Banja La Mtsogolo
DHS: Demographic and Health Survey
HSPH: Harvard T. H. Chan School of Public Health
IPA: Innovations for Poverty Action
IRB: Institutional Review Board
ITT: intent-to-treat
IUD: intrauterine device
MDHS: Malawi Demographic and Health Survey
MOH: Ministry of Health
NHSRC: National Health Sciences Research Committee
NSO: National Statistics Office
PPFP: postpartum family planning
PSI: Population Services International
RHD: Reproductive Health Directorate
WHO: World Health Organization
