# Supplementary material for: The Effect of Improved Access to Family Planning on Postpartum Women: Protocol for a Randomized Controlled Trial
Source: JMIR Res Protoc. 2020 Aug 14;9(8):e16697. doi: 10.2196/16697 (PMC7455875; doi:10.2196/16697)
Supplement: Multimedia Appendix 1 [file resprot_v9i8e16697_app1.pdf]

MALAWI FAMILY PLANNING STUDY

WAVE 1 BASELINE SURVEY

Survey ID Generation

1. Field Manager ID Number ☐
   1. Field Manager 1
   2. Field Manager 2
2. Enumerator Identification Number (Enter 01-10): ☐☐
3. Survey Wave (Enter 1-3): ☐
4. District (Enter 1 for Lilongwe): ☐
5. Area Code (2 digits): ☐☐
6. Sector Code (2 digits): ☐☐
7. Cluster Number (2 digits): ☐☐
8. Household ID Number - 2 digits describing the number of the household that the enumerator is completing for that particular day. For example, if the household is the fifth being interviewed by that particular enumerator that day, the household ID number is ‘05’. ☐☐

The **survey ID number** is a 13-digit number that is created by joining the 8 numerical responses recorded above in order, from response 1 to response 8. An example of a complete respondent ID number is given below.

Example: For the 13th household that is interviewed by Enumerator 04 in Field Manager 1’s team for the baseline survey (Wave 1) in Lilongwe (District 1) Area 47 (code 47), Sector 6 (code 06), Cluster 12, the **survey ID number** will be created as:

1	0	4	1	1	4	7	0	6	1	2	1	3

SURVEY ID NUMBER: ☐☐☐☐☐☐☐☐☐☐☐☐☐

Enter assigned number above and in the Interviewer Log Book.

PROCEED TO WOMAN RECRUITMENT SCRIPT – PAGE 2

WOMAN Recruitment Script – FIRST VISIT (Verbal, In Person)

INTRODUCTION

Hello. My name is _________________ and I am from Innovations for Poverty Action (IPA) Malawi based in Lilongwe. I would like to invite you to participate in a study in which we are trying to understand use of family planning and reproductive health services in Lilongwe, Malawi. We are interested in trying to understand some of the issues that married women face concerning family planning, maternal and child health, and childbearing. We are also collecting information about households. You may participate if:

- you are a married woman;
- you are between the ages of 18 to 35;
- you live in Lilongwe;
- you are either pregnant now or have given birth within the past 6 months

If you do not meet all of the requirements that I mentioned, please tell me now. (GO TO QUESTION 1)

DETERMINING ELIGIBILITY

No.	Question	Response	Skip Rules
1	Do you meet all of these requirements?	YES NO	IF NO, GO TO 2A<br>IF YES, GO TO 2B
2A	Thank you. Are there any other women who live in this household?	YES NO	IF NO, GO TO **END**<br>IF YES, GO TO 3
2B	Thank you. Are there any other women who live in this household?	YES NO	IF NO, GO TO **PURPOSE**.<br>IF YES, GO TO 3.
3	With your permission, I would meet them to see if they are eligible for this study.<br>MEET OTHER WOMEN IN THE HOUSEHOLD.<br>REPEAT INTRODUCTION TO EACH WOMAN.<br>IDENTIFY THE **YOUNGEST** ELIGIBLE WOMAN IN THE HOUSEHOLD.	YES NO	IF **YOUNGEST** ELIGIBLE WOMAN IS IDENTIFIED, GO TO **PURPOSE**.<br>IF **NO** ELIGIBLE WOMAN IS IDENTIFIED, GO TO **END**
**END**	Thank you for your time. I hope that you have a good day.		END VISIT.

PURPOSE

(TALK TO THE YOUNGEST ELIGIBLE WOMAN IN THE HOUSEHOLD)

NAME OF YOUNGEST ELIGIBLE WOMAN: ____

We want to talk to women like you who live in Lilongwe so that we can identify the best ways to help our community access family planning, particularly for women who are pregnant or who have just recently given

birth. We feel that perspectives from people like you can help to inform the Ministry of Health's approach to identifying potential barriers to use of family planning services so that we can then improve those services.

If you decide to participate, you will be asked to participate in three individual surveys over a two year period. Each of these surveys will take approximately 75 minutes in total. In each survey, you will be asked several questions. Some of them will be about marriage, family planning, pregnancy, and your children. Others will be about education and employment. If you agree, you will participate in the first survey today, and we will return after one year and after two years to conduct the second and third surveys with you. By returning after some time, we can learn more about how life changes for Malawian families.

I do not anticipate any physical risks to participating. Your responses to survey questions will be kept confidential, and at no time will your actual identity be revealed. Your participation may help to inform the community of the local family planning environment and may also help to improve access to family planning services in Lilongwe. The data you give me will be used for academic publications and may be used as the basis for articles or presentations in the future. That said, we won't use your name or information that would identify you in any publications or presentations. (GO TO QUESTION 5)

No.	Question	Response	Skip Rules
5	If you would like to participate in this study, please let me know now.	YES NO	IF NO, GO TO **END**<br>IF YES, GO TO 6
6	Thank you for your participation. Is now a good time to begin the first survey?	YES NO	IF NO, GO TO **END**<br>IF YES, GO TO **CONTACT**
**END**	Thank you for your time. I hope that you have a good day.		END VISIT.

CONTACT

Thank you. I will begin the survey shortly. Do you have any questions now? If you have questions later, you can contact one of the researchers, Dr. Bagrey Ngwira or Ms. Carly Farver, at:

Dr. Bagrey Ngwira
Innovations for Poverty Action (IPA) Malawi
E-mail: bagreyngwira@gmail.com
Telephone: +265 999554003
Availability: Monday to Friday, 9 AM to 5 PM

Ms. Carly Farver
Innovations for Poverty Action (IPA) Malawi
E-mail: CFarver@poverty-action.org
Telephone: +265 994447291
Availability: Monday to Friday, 9 AM to 5 PM

Thank you for your time. DATE: ________________________________

PROCEED TO WOMAN CONSENT FORM – PAGE 4

Protocol Title: **The Malawi Family Planning Study 2016, Wave I**	
Principal Investigator: **David Canning, Ph.D.**	
Description of Study Population: **Married women aged 18-35, living in Lilongwe, and are either currently pregnant or have given birth within the past 6 months**	
Version Date: **4 May 2016**	

Malawi Family Planning Study (MFPS) 2016
Field Research Informed Consent Form

About this consent form

Please read this form carefully. This form provides important information about participating in research. You have the right to take your time in making decisions about participating in this research. You may discuss your decision with your family, your friends and/or your doctor. If you have any questions about the research or any portion of this form, please ask us. If you decide to participate in this research you will be asked to sign this form. A copy of the signed form will be provided to you for your record.

Participation is voluntary

You are invited to take part in this research because:

- you are a married woman;
- you are between the ages of 18 to 35;
- you live in Lilongwe;
- you are either pregnant now or have given birth within the past 6 months

It is your choice whether or not to participate. If you choose to participate, you may change your mind and leave the study at any time. Refusal to participate or stopping your participation will involve no penalty or loss of benefits to which you are otherwise entitled.

What you should know about a research study

Someone will explain this research study to you.
A research study is something you volunteer for.
Whether or not you take part is up to you.
You can choose not to take part in the research study.
You can agree to take part now and later change your mind.
Whatever you decide it will not be held against you.
Feel free to ask all the questions you want before you decide.

What is the purpose of this research?

The purpose of this research is:

1. To understand use of family planning and reproductive health services among married women in Lilongwe, Malawi.
2. To learn about issues concerning family planning, maternal and child health, and childbearing.
3. To collect household-level health, demographic, and socioeconomic information over time.

How many people will take part in this research?

About 2,000 people will take part in this research.

How long will I take part in this research?

It will take about 2 years to complete the entire study. During this time, you will be asked to participate in 3 individual surveys.

What can I expect if I take part in this research?

As a participant, you will be asked to participate in 3 individual surveys over a two year period. Each of these surveys will take approximately 75 minutes. In each survey, you will be asked several questions. Some of them will be about marriage, family planning, pregnancy, and your children. Others will be about education and employment. As part of the survey, we will also measure your height and weight. We will then collect 2 to 3 blood drops from your finger to test for hemoglobin, which is a marker for anemia. We will provide you with your results and an explanation of what the results mean. Although these results are for research purposes only and are not diagnostic, we will give you a referral letter to the closest health facility if we find any results that are outside the normal range for you to check again. Finally, we will also collect information that will help us to contact you for future interviews. This information includes: your household address and location, your mobile phone number, and the contact information of two people whom you know and who do not live in your household, but would know how to reach you. With your permission, we would also like to take your photo, which will help us to identify and contact you in the future.

If you agree, you will participate in the first survey today, and we will return after one year and after two years to conduct the second and third surveys with you. By returning after some time, we can learn more about how life changes for Malawian families. The information that we gather from you and from other community members will help us better understand key issues in women and children's health in Malawi. You may choose, without any penalty, to skip any questions, to discontinue the survey at any time, or to exclude use of your responses.

What are the risks and possible discomforts?

I do not anticipate any major physical risks to participating. However, some of the questions may cause discomfort or embarrassment. I also do not anticipate any major physical risks to having you participate in the height and weight assessment. Some risks of blood drawing, which is a part of the anemia test, are pain from the finger prick, light-headedness, and very rarely, infection.

Are there any benefits from being in this research study?

We cannot promise any benefits to you or others from your taking part in this research. However, this is a chance for you to share your thoughts and experiences regarding marriage, children, fertility, and family planning. Your participation will help to inform the community of the local family planning environment and may also help to improve access to family planning services in Lilongwe. You may also be eligible to receive additional health information and/or services that are available in Lilongwe.

What are my alternatives to participating in this research?

The alternative to participating in this research study is not to participate. Your participation in this study is completely voluntary, and you may refuse to participate or withdraw from the study without penalty.

Will I be compensated for participating in this research?

You will not be compensated for participating in this research.

What will I have to pay for if I participate in this research?
It will not cost you anything to participate in this research.

What happens if I am injured as a result of participating in this research study?
If physical injury resulting from participation in this research should occur, although policy at Harvard University and IPA Malawi is not to provide compensation, medical treatment will be available including first aid, emergency treatment and follow-up care as needed, and your insurance carrier may be billed for the cost of such treatment. In making such medical treatment available, or providing it, the persons conducting this research project are not admitting that your injury was their fault.

Can my taking part in the research end early?
You may decide not to continue in the research at any time without it being held against you. You may withdraw by informing me that you no longer wish to participate (no questions will be asked). You may also skip any question during the survey, but continue to participate in the rest of the study. The person in charge of the research can also remove you from the research at any time without your approval for any reason. If you decide to leave the research, please contact the investigator.

If I take part in this research, how will my privacy be protected? What happens to the information you collect?
Your responses to survey questions will be kept confidential. The results of your height and weight assessment and anemia test are private and will also be kept confidential. At no time will your actual identity be revealed. You will be assigned a random numerical code. Anyone who helps me with this research will only know you by this code. The key code linking your name with your number will be kept in a locked file cabinet in a locked office in the United States, and no one else will have access to it. It will be destroyed as soon as I have finished analyzing your responses to my questions. The data you give me will be used for academic articles that are currently being written and may be used as the basis for articles or presentations in the future. We won't use your name or information that would identify you in any publications or presentations. Your name and other identifying information will always be kept anonymous.

Data collected, including your identifiable information, may be seen by the Harvard Institutional Review Board (IRB) that oversees the research.

If I have any questions, concerns or complaints about this research study, who can I talk to?

If you have questions or concerns about this research, please contact **Mr. Mahesh Karra, Ms. Carly Farver,** or **Dr. Bagrey Ngwira**, who are locally in charge of this study.

Dr. Bagrey Ngwira
Innovations for Poverty Action (IPA) Malawi
E-mail: bagreyngwira@gmail.com
Telephone: +265 999554003
Availability: Monday to Friday, 9 AM to 5 PM

Ms. Carly Farver
Innovations for Poverty Action (IPA) Malawi
E-mail: CFarver@poverty-action.org
Telephone: +265 994447291
Availability: Monday to Friday, 9 AM to 5 PM

Mahesh Karra, M.Sc.
Harvard School of Public Health
Department of Global Health and Population
E-mail: mkarra@mail.harvard.edu
U.S. Telephone: +1-703-969-1183
Local Telephone: +265 881841735
Availability: Monday to Friday, 9 AM to 5 PM

Please contact Mr. Karra, Ms. Farver, or Dr. Ngwira:

- If you have questions, concerns, or complaints,
- If you would like to talk to the research team,
- If you think the research has hurt you, or
- If you wish to withdraw from the study.

The Principal Investigator of this study is **Dr. David Canning**. He can be reached at:

Harvard School of Public Health
Department of Global Health and Population
E-mail: dcanning@hsph.harvard.edu
Telephone: +1-617-432-6336
Availability: Monday to Friday, 9 AM to 5 PM

This research has been reviewed by the Malawi National Health Sciences Research Committee (NHSRC) and by a Harvard Longwood Medical Area Institutional Review Board (HMS/HSDM or HSPH). If you wish to speak with someone from the IRB, please contact the Office of Human Research Administration (OHRA) at 617-432-2157 (or toll-free at 1-866-606-0573) or 90 Smith Street, Boston, Massachusetts 02120 for any of the following:

- If your questions, concerns, or complaints are not being answered by the research team,
- If you cannot reach the research team,
- If you want to talk to someone besides the research team,
- If you have questions about your rights as a research participant, or
- If you want to get information or provide input about this research.

You may also contact the Malawi NHSRC at Ministry of Health, P.O. Box 30377, Lilongwe 3, Malawi, by phone at +265 1 726 422/418, or by e-mail at mohdoccentre@gmail.com.

Statement of Consent

I have read the information in this consent form including risks and possible benefits. All my questions about the research have been answered to my satisfaction. I understand that I am free to withdraw at any time without penalty or loss of benefits to which I am otherwise entitled.

I consent to participate in the study.

SIGNATURE

Your signature below indicates your permission to take part in this research

____
Name of participant

______________________________ ______________
Signature of participant Date

______________________________ ______________
Signature of person obtaining consent Date

____
Printed name of person obtaining consent

PROCEED TO FINANCIAL RESPONDENT RECRUITMENT SCRIPT – PAGE 9

FINANCIAL RESPONDENT
Recruitment Script (Verbal, In Person)

(TO THE YOUNGEST ELIGIBLE WOMAN):

IDENTIFICATION OF FINANCIAL RESPONDENT

Thank you for consenting to participate in this survey. To begin, I would like to ask some questions about this household in order to learn about the lives of Malawian families. For this part, I would like to speak to the most financially knowledgeable person in this household and who is over 18 years of age.

Are you the most financially knowledgeable person in this household at this time? YES NO

IF YES: Thank you. I will begin this part of the interview shortly.

In this part of the study, you will be asked to participate in three household surveys over a two year period. Each of these surveys will take approximately 45 minutes. In each survey, you will be asked several questions about your household, including questions about household assets, purchases, and expenditures. If you agree, you will participate in the first survey today, and we will return after one year and after two years to conduct the second and third surveys with you. By returning after some time, we can learn about how life changes for Malawian families.

PROCEED TO FINANCIAL RESPONDENT CONSENT FORM – PAGE 12

IF NO: Could you tell me who the most financially knowledgeable person is in this household who is available to speak to me now?

NAME OF FINANCIAL RESPONDENT: ____

Thank you. I will interview this person (NAME OF FINANCIAL RESPONDENT) first and will return to interview you in about 45 minutes. I look forward to our interview.

APPROACH IDENTIFIED FINANCIAL RESPONDENT

FINANCIAL RESPONDENT
Recruitment Script (Verbal, In Person)

(TO THE IDENTIFIED FINANCIAL RESPODENT)

INTRODUCTION

Hello. My name is _________________ and I am from Innovations for Poverty Action (IPA) Malawi based in Lilongwe. I would like to invite you to participate in a study in which we are trying to understand use of family planning and health services in Lilongwe, Malawi. As part of this study, we are also collecting information on households to learn about the lives of Malawian families.

You have been identified by (NAME OF YOUNGEST ELIGIBLE WOMAN) as the most financially knowledgeable person who is in this household. You may participate if:

- you are at least 18 years of age
- you are a resident of this household
- you are the most financially knowledgeable person in this household at this time.

If you do not meet all of the requirements that I mentioned, please let me know now.

Do you meet these criteria? YES NO

If NO: Thank you for your time. Could you tell me who the most financially knowledgeable person is in this household who is available to speak to me now?

NAME OF FINANCIAL RESPONDENT: ____

REPEAT INTRODUCTION UNTIL AN ELIGIBLE FINANCIAL RESPONDENT IS IDENTIFIED.

IF YES: Thank you.

PURPOSE

We want to talk to people like you who live in Lilongwe so that we can identify the best ways to help our community. We feel that local perspectives from people like you can help to inform the Ministry of Health's approach to identifying potential barriers to accessing services so that we can then improve those services.

If you decide to participate, you will be asked to participate in three household surveys over a two year period. Each of these surveys will take approximately 45 minutes. In each survey, you will be asked several questions about your household, including questions about household assets, purchases, and expenditures. If you agree, you will participate in the first survey today, and we will return after one year and after two years to conduct the second and third surveys with you. By returning after some time, we can learn about how life changes for Malawian families.

I do not anticipate any physical risks to participating. Your responses to survey questions will be kept confidential, and at no time will your actual identity be revealed. The data you give me will be used for academic publications and may be used as the basis for articles or presentations in the future. That said, we won't use your name or information that would identify you in any publications or presentations.

If you would like to participate in this study, please let me know now. YES NO

If NO, OR IF RESPONDENT CANNOT BE INTERVIEWED RIGHT AWAY: Thank you for your time. Could you tell me who the most financially knowledgeable person is in this household who is available to speak to me now?

NAME: ____

REPEAT INTRODUCTION AND PURPOSE UNTIL AN ELIGIBLE FINANCIAL RESPONDENT IS IDENTIFIED.

If YES: Thank you for your participation. I will begin the interview shortly. Do you have any questions now? If you have questions later, you can contact one of the researchers, Dr. Bagrey Ngwira or Ms. Carly Farver, at:

Dr. Bagrey Ngwira
Innovations for Poverty Action (IPA) Malawi
E-mail: bagreyngwira@gmail.com
Telephone: +265 999554003
Availability: Monday to Friday, 9 AM to 5 PM

Ms. Carly Farver
Innovations for Poverty Action (IPA) Malawi
E-mail: CFarver@poverty-action.org
Telephone: +265 994447291
Availability: Monday to Friday, 9 AM to 5 PM

Thank you for your time.

PROCEED TO FINANCIAL RESPONDENT CONSENT FORM – PAGE 12

Protocol Title: **The Malawi Family Planning Study 2014, Wave I**	
Principal Investigator: **David Canning, Ph.D.**	
Description of Study Population: **Financially knowledgeable respondents aged 18 years or older, living in Lilongwe in the same households as the selected women respondents**	
Version Date: **4 May 2016**	

Malawi Family Planning Study (MFPS) 2014
Field Research Informed Consent Form

About this consent form

Please read this form carefully. This form provides important information about participating in research. You have the right to take your time in making decisions about participating in this research. You may discuss your decision with your family, your friends and/or your doctor. If you have any questions about the research or any portion of this form, please ask us. If you decide to participate in this research you will be asked to sign this form. A copy of the signed form will be provided to you for your record.

Participation is voluntary

You are invited to take part in this research because:

- you are over 18 years old;
- you are the most financially knowledgeable person in this household who is present at this time

It is your choice whether or not to participate. If you choose to participate, you may change your mind and leave the study at any time. Refusal to participate or stopping your participation will involve no penalty or loss of benefits to which you are otherwise entitled.

What you should know about a research study

Someone will explain this research study to you.
A research study is something you volunteer for.
Whether or not you take part is up to you.
You can choose not to take part in the research study.
You can agree to take part now and later change your mind.
Whatever you decide it will not be held against you.
Feel free to ask all the questions you want before you decide.

What is the purpose of this research?

The purpose of this research is:

4. To understand use of family planning and reproductive health services among married women in Lilongwe, Malawi.
5. To learn about issues concerning family planning, maternal and child health, and childbearing.
6. To collect household-level health, demographic, and socioeconomic information over time.

How many people will take part in this research?

About 2,000 people will take part in this research.

How long will I take part in this research?

It will take about 2 years to complete the entire study. During this time, you will be asked to participate in 3 household surveys.

What can I expect if I take part in this research?

As a participant, you will be asked to participate in 3 household surveys over a two year period. Each of these surveys will take approximately 45 minutes. In each survey, you will be asked several questions about your household, including questions about household assets, purchases, and expenditures. Finally, we will also collect information that will help us to contact you for future interviews. This information includes: your household address and location, your mobile phone number, and the contact information of two people whom you know and who do not live in your household, but would know how to reach you. With your permission, we would also like to take a photo of you and a photo of the front of your house, both of which will help us to identify and contact you in the future.

If you agree, you will participate in the first survey today, and we will return after one year and after two years to conduct the second and third surveys with you. By returning after some time, we can learn more about how life changes for Malawian families. The information that we gather from you and from other community members will help us better understand key issues about health and family life in Malawi. You may choose, without any penalty, to skip any questions, to discontinue the survey at any time, or to exclude use of your responses.

What are the risks and possible discomforts?

I do not anticipate any major physical risks to participating. However, some of the questions may cause discomfort or embarrassment.

Are there any benefits from being in this research study?

We cannot promise any benefits to you or others from your taking part in this research. However, this is a chance for you to share your thoughts and experiences. Your participation will help to inform the community of the local environment and may also help to improve access to services in Lilongwe.

What are my alternatives to participating in this research?

The alternative to participating in this research study is not to participate. Your participation in this study is completely voluntary, and you may refuse to participate or withdraw from the study without penalty.

Will I be compensated for participating in this research?

You will not be compensated for participating in this research.

What will I have to pay for if I participate in this research?

It will not cost you anything to participate in this research.

What happens if I am injured as a result of participating in this research study?

If physical injury resulting from participation in this research should occur, although policy at Harvard University and IPA Malawi is not to provide compensation, medical treatment will be available including first aid, emergency treatment and follow-up care as needed, and your insurance carrier may be billed for the cost of such treatment. In making such medical treatment available, or providing it, the persons conducting this research project are not admitting that your injury was their fault.

Can my taking part in the research end early?

You may decide not to continue in the research at any time without it being held against you. You may withdraw by informing me that you no longer wish to participate (no questions will be asked). You may also skip any question during the survey, but continue to participate in the rest of the study. The person in charge of the research can also remove you from the research at any time without your approval for any reason. If you decide to leave the research, please contact the investigator.

If I take part in this research, how will my privacy be protected? What happens to the information you collect?

Your responses to survey questions will be kept confidential. At no time will your actual identity be revealed. You will be assigned a random numerical code. Anyone who helps me with this research will only know you by this code. The key code linking your name with your number will be kept in a locked file cabinet in a locked office in the United States, and no one else will have access to it. It will be destroyed as soon as I have finished analyzing your responses to my questions. The data you give me will be used for academic articles that are currently being written and may be used as the basis for articles or presentations in the future. We won't use your name or information that would identify you in any publications or presentations. Your name and other identifying information will always be kept anonymous.

Data collected, including your identifiable information, may be seen by the Harvard Institutional Review Board (IRB) that oversees the research.

If I have any questions, concerns or complaints about this research study, who can I talk to?

If you have questions or concerns about this research, please contact **Mr. Mahesh Karra, Ms. Carly Farver,** or **Dr. Bagrey Ngwira**, who are locally in charge of this study.

Dr. Bagrey Ngwira
Innovations for Poverty Action (IPA) Malawi
E-mail: bagreyngwira@gmail.com
Telephone: +265 999554003
Availability: Monday to Friday, 9 AM to 5 PM

Ms. Carly Farver
Innovations for Poverty Action (IPA) Malawi
E-mail: CFarver@poverty-action.org
Telephone: +265 994447291
Availability: Monday to Friday, 9 AM to 5 PM

Mahesh Karra, M.Sc.
Harvard School of Public Health
Department of Global Health and Population
E-mail: mkarra@mail.harvard.edu
U.S. Telephone: +1-703-969-1183
Local Telephone: +265 881841735
Availability: Monday to Friday, 9 AM to 5 PM

Please contact Mr. Karra, Ms. Farver, or Dr. Ngwira:

- If you have questions, concerns, or complaints,
- If you would like to talk to the research team,
- If you think the research has hurt you, or
- If you wish to withdraw from the study.

The Principal Investigator of this study is **Dr. David Canning**. He can be reached at:

Harvard School of Public Health
Department of Global Health and Population
E-mail: dcanning@hsph.harvard.edu
Telephone: +1-617-432-6336
Availability: Monday to Friday, 9 AM to 5 PM

This research has been reviewed by the Malawi National Health Sciences Research Committee (NHSRC) and by a Harvard Longwood Medical Area Institutional Review Board (HMS/HSDM or HSPH). If you wish to speak with someone from the IRB, please contact the Office of Human Research Administration (OHRA) at 617-432-2157 (or toll-free at 1-866-606-0573) or 90 Smith Street, Boston, Massachusetts 02120 for any of the following:

- If your questions, concerns, or complaints are not being answered by the research team,
- If you cannot reach the research team,
- If you want to talk to someone besides the research team,
- If you have questions about your rights as a research participant, or
- If you want to get information or provide input about this research.

You may also contact the Malawi NHSRC at Ministry of Health, P.O. Box 30377, Lilongwe 3, Malawi, by phone at +265 1 726 422/418, or by e-mail at mohdoccentre@gmail.com.

Statement of Consent

I have read the information in this consent form including risks and possible benefits. All my questions about the research have been answered to my satisfaction. I understand that I am free to withdraw at any time without penalty or loss of benefits to which I am otherwise entitled.

I consent to participate in the study.

SIGNATURE

Your signature below indicates your permission to take part in this research

____
Name of participant

____ ____________________
Signature of participant Date

____ ____________________
Signature of person obtaining consent Date

____
Printed name of person obtaining consent

PROCEED TO SURVEY – PAGE HH-0

DATE ____________________

QUANTITATIVE SURVEY
HOUSEHOLD QUESTIONNAIRE
MALAWI FAMILY PLANNING SURVEY - WAVE I, 2016
HARVARD UNIVERSITY, IPA MALAWI

IDENTIFICATION (1)

NAME AND NO. OF THE DISTRICT ____________________

AREA . . . .

SECTOR . . . .

CLUSTER NUMBER . . . .

HOUSEHOLD ID NUMBER . . . .

HOUSEHOLD ADDRESS ____________________

DESCRIPTION OF HOUSE/ LANDMARKS ____________________

NAME OF HOUSEHOLD HEAD ____________________

PRIMARY PHONE NO. OF HH HEAD ____________________

NAME AND LINE NUMBER OF RESPONDENT ____________________

PRIMARY PHONE NO. OF RESPONDENT ____________________

ALTERNATE PHONE NO. OF RESPONDENT ____________________

E-MAIL OF RESPONDENT ____________________

GPS COORDINATES

LATITUDE N/S [ ] D D [ ][ ] X X [ ][ ] . X X X [ ][ ][ ]

LONGITUDE E/W [ ] D D [ ][ ] X X [ ][ ] . X X X [ ][ ][ ]

INTERVIEWER VISITS

	1	2	3	FINAL VISIT
DATE	D D M M Y Y Y Y			DAY / MONTH / YEAR
INTERVIEWER'S NAME				INT. NUMBER
RESULT*				RESULT
NEXT VISIT: DATE				TOTAL NUMBER OF VISITS
TIME				

*RESULT CODES:
1 COMPLETED
2 NO HOUSEHOLD MEMBER AT HOME OR NO COMPETENT RESPONDENT AT HOME AT TIME OF VISIT
3 ENTIRE HOUSEHOLD ABSENT FOR EXTENDED PERIOD OF TIME
4 POSTPONED
5 REFUSED
6 DWELLING VACANT OR ADDRESS NOT A DWELLING
7 DWELLING DESTROYED
8 DWELLING NOT FOUND
9 MOVED TO ANOTHER VILLAGE/TOWN/DISTRICT
A MOVED TO NEIGHBORING COUNTRY
B SHIFTED TO UNKNOWN LOCATION
C TRANSFERRED DUE TO WORK/EDUCATION
D OTHER ______________________ (SPECIFY)

TOTAL PERSONS IN HOUSEHOLD

LINE NO. OF RESPONDENT TO HOUSEHOLD QUESTIONNAIRE

FIELD MANAGER	INTERVIEWER	OFFICE EDITOR	KEYED BY
NAME ____________	NAME ____________		

THIS PAGE IS INTENTIONALLY BLANK

In this section, I would like to ask you some questions about your household. The questions in this section usually take about 15 to 30 minutes. All of the answers you give will be confidential and will not be shared with anyone other than members of our survey team. You don't have to be in the survey, but we hope you will agree to answer the questions since your views are important. If I ask you any question you don't want to answer, just let me know and I will go on to the next question or you can stop the interview at any time.

In case you need more information about the survey, you may contact the person listed on the card that has been given to your household.

PRESENT CARD WITH CONTACT INFORMATION TO REMIND RESPONDENT

Do you have any questions?
May I begin the interview now?

HOUSEHOLD SCHEDULE

							IF AGE 15 OR OLDER			
LINE NO.	USUAL RESIDENTS AND VISITORS	RELATIONSHIP TO HEAD OF HOUSEHOLD	SEX	RESIDENCE		AGE	MARITAL STATUS	ELIGIBILITY		
**1**	**2**	**3**	**4**	**5**	**6**	**7**	**8**	**9**	**10**	**11**
	Please give me the names of the persons who usually live in your household and guests of the household who stayed here last night, starting with the head of the household.<br><br>AFTER LISTING THE NAMES AND RECORDING THE RELATIONSHIP AND SEX FOR EACH PERSON, ASK QUESTIONS 2A-2C TO BE SURE THAT THE LISTING IS COMPLETE.<br><br>THEN ASK APPROPRIATE QUESTIONS IN COLUMNS 5-20 FOR EACH PERSON.	What is the relationship of (NAME) to the head of the household?<br><br>SEE CODES BELOW.	Is (NAME) male or female?	Does (NAME) usually live here?	Did (NAME) stay here last night?	How old was (NAME) at his/her last birthday?<br><br>IF 95 OR MORE, RECORD '95'.	What is (NAME)'s current marital status?<br><br>1 = MARRIED OR LIVING TOGETHER<br>2 = DIVORCED/ SEPARATED<br>3 = WIDOWED<br>4 = NEVER-MARRIED AND NEVER LIVED TOGETHER	CIRCLE LINE NUMBER OF THE ELIGIBLE WOMAN	CIRCLE LINE NUMBER OF THE RESP-ONDENT	CIRCLE LINE NUMBER OF ALL THE ELIGIBLE WOMAN'S CHILDREN BETWEEN AGE 0-5
			M F	Y N	Y N	IN YEARS				
01		☐☐	1 2	1 2	1 2	☐☐	☐	01	01	01
02		☐☐	1 2	1 2	1 2	☐☐	☐	02	02	02
03		☐☐	1 2	1 2	1 2	☐☐	☐	03	03	03
04		☐☐	1 2	1 2	1 2	☐☐	☐	04	04	04
05		☐☐	1 2	1 2	1 2	☐☐	☐	05	05	05
06		☐☐	1 2	1 2	1 2	☐☐	☐	06	06	06
07		☐☐	1 2	1 2	1 2	☐☐	☐	07	07	07
08		☐☐	1 2	1 2	1 2	☐☐	☐	08	08	08
09		☐☐	1 2	1 2	1 2	☐☐	☐	09	09	09
10		☐☐	1 2	1 2	1 2	☐☐	☐	10	10	10

CODES FOR Q. 3: RELATIONSHIP TO HEAD OF HOUSEHOLD

01 = HEAD
02 = WIFE OR HUSBAND
03 = SON OR DAUGHTER
04 = SON-IN-LAW OR DAUGHTER-IN-LAW
05 = GRANDCHILD
06 = PARENT
07 = PARENT-IN-LAW
08 = BROTHER OR SISTER
09 = OTHER RELATIVE
10 = ADOPTED/FOSTER/ STEPCHILD
11 = NOT RELATED
98 = DON'T KNOW

	IF AGE 5 YEARS OR OLDER		IF AGE 5-24 YEARS		IF AGE 0-4 YEARS
LINE NO.	EVER ATTENDED SCHOOL		CURRENT/RECENT SCHOOL ATTENDANCE		BIRTH REGIS-TRATION
	16	17	18	19	20
	Has (NAME) ever attended school?	What is the highest level of school (NAME) has attended?<br><br>SEE CODES BELOW.<br><br>What is the highest grade (NAME) completed at that level?<br><br>SEE CODES BELOW.	Did (NAME) attend school at any time during the (2016-2017) **(2)** school year?	During this/that school year, what level and grade [is/was] (NAME) attending?<br><br>SEE CODES BELOW.	Does (NAME) have a birth certificate?<br><br>IF NO, PROBE: Has (NAME)'s birth ever been registered with the civil authority?<br><br>1 = HAS CERTIFICATE<br>2 = REGISTERED<br>3 = NEITHER<br>8 = DON'T KNOW
	Y N	LEVEL GRADE	Y N	LEVEL GRADE	
01	1 2 → NEXT LINE		1 2 → NEXT LINE		
02	1 2 → NEXT LINE		1 2 → NEXT LINE		
03	1 2 → NEXT LINE		1 2 → NEXT LINE		
04	1 2 → NEXT LINE		1 2 → NEXT LINE		
05	1 2 → NEXT LINE		1 2 → NEXT LINE		
06	1 2 → NEXT LINE		1 2 → NEXT LINE		
07	1 2 → NEXT LINE		1 2 → NEXT LINE		
08	1 2 → NEXT LINE		1 2 → NEXT LINE		
09	1 2 → NEXT LINE		1 2 → NEXT LINE		
10	1 2 → NEXT LINE		1 2 → NEXT LINE		

CODES FOR Qs. 17 AND 19: EDUCATION

LEVEL	GRADE
1 = PRIMARY	00 = LESS THAN 1 YEAR COMPLETED (USE '00' FOR Q. 17 ONLY. THIS CODE IS NOT ALLOWED FOR Q. 19)
2 = SECONDARY	
3 = HIGHER	
6 = PRE-PRIMARY	
8 = DON'T KNOW	98 = DON'T KNOW

							IF AGE 15 OR OLDER			
LINE NO.	USUAL RESIDENTS AND VISITORS	RELATIONSHIP TO HEAD OF HOUSEHOLD	SEX	RESIDENCE		AGE	MARITAL STATUS	ELIGIBILITY		
**1**	**2**	**3**	**4**	**5**	**6**	**7**	**8**	**9**	**10**	**11**
	Please give me the names of the persons who usually live in your household and guests of the household who stayed here last night, starting with the head of the household.<br><br>AFTER LISTING THE NAMES AND RECORDING THE RELATIONSHIP AND SEX FOR EACH PERSON, ASK QUESTIONS 2A-2C TO BE SURE THAT THE LISTING IS COMPLETE.<br><br>THEN ASK APPROPRIATE QUESTIONS IN COLUMNS 5-20 FOR EACH PERSON.	What is the relationship of (NAME) to the head of the household?<br><br>SEE CODES BELOW.	Is (NAME) male or female?	Does (NAME) usually live here?	Did (NAME) stay here last night?	How old was (NAME) at his/her last birthday?<br><br>IF 95 OR MORE, RECORD '95'.	What is (NAME)'s current marital status?<br><br>1 = MARRIED OR LIVING TOGETHER<br>2 = DIVORCED/ SEPARATED<br>3 = WIDOWED<br>4 = NEVER-MARRIED AND NEVER LIVED TOGETHER	CIRCLE LINE NUMBER OF THE ELIGIBLE WOMAN	CIRCLE LINE NUMBER OF THE RESP-ONDENT	CIRCLE LINE NUMBER OF ALL THE ELIGIBLE WOMAN'S CHILDREN BETWEEN AGE 0-5
			M F	Y N	Y N	IN YEARS				
11		☐☐	1 2	1 2	1 2	☐☐	☐	11	11	11
12		☐☐	1 2	1 2	1 2	☐☐	☐	12	12	12
13		☐☐	1 2	1 2	1 2	☐☐	☐	13	13	13
14		☐☐	1 2	1 2	1 2	☐☐	☐	14	14	14
15		☐☐	1 2	1 2	1 2	☐☐	☐	15	15	15
16		☐☐	1 2	1 2	1 2	☐☐	☐	16	16	16
17		☐☐	1 2	1 2	1 2	☐☐	☐	17	17	17
18		☐☐	1 2	1 2	1 2	☐☐	☐	18	18	18
19		☐☐	1 2	1 2	1 2	☐☐	☐	19	19	19
20		☐☐	1 2	1 2	1 2	☐☐	☐	20	20	20

TICK HERE IF CONTINUATION SHEET USED ☐

2A) Just to make sure that I have a complete listing: are there any other persons such as small children or infants that we have not listed? YES ☐ → ADD TO TABLE NO ☐

2B) Are there any other people who may not be members of your family, such as domestic servants, lodgers, or friends who usually live here? YES ☐ → ADD TO TABLE NO ☐

2C) Are there any guests or temporary visitors staying here, or anyone else who stayed here last night, who have not been listed? YES ☐ → ADD TO TABLE NO ☐

CODES FOR Q. 3: RELATIONSHIP TO HEAD OF HOUSEHOLD

01 = HEAD
02 = WIFE OR HUSBAND
03 = SON OR DAUGHTER
04 = SON-IN-LAW OR DAUGHTER-IN-LAW
05 = GRANDCHILD
06 = PARENT
07 = PARENT-IN-LAW
08 = BROTHER OR SISTER
09 = OTHER RELATIVE
10 = ADOPTED/FOSTER/ STEPCHILD
11 = NOT RELATED
98 = DON'T KNOW

	IF AGE 5 YEARS OR OLDER		IF AGE 5-24 YEARS		IF AGE 0-4 YEARS
LINE NO.	EVER ATTENDED SCHOOL		CURRENT/RECENT SCHOOL ATTENDANCE		BIRTH REGIS-TRATION
	**16**	**17**	**18**	**19**	**20**
	Has (NAME) ever attended school?	What is the highest level of school (NAME) has attended?<br><br>SEE CODES BELOW.<br><br>What is the highest grade (NAME) completed at that level?<br><br>SEE CODES BELOW.	Did (NAME) attend school at any time during the (2016-2017) **(2)** school year?	During this/that school year, what level and grade [is/was] (NAME) attending?<br><br>SEE CODES BELOW.	Does (NAME) have a birth certificate?<br><br>IF NO, PROBE: Has (NAME)'s birth ever been registered with the civil authority?<br><br>1 = HAS CERTIFICATE<br>2 = REGISTERED<br>3 = NEITHER<br>8 = DON'T KNOW
	Y N	LEVEL GRADE	Y N	LEVEL GRADE	
11	1 2 → NEXT LINE		1 2 → NEXT LINE		
12	1 2 → NEXT LINE		1 2 → NEXT LINE		
13	1 2 → NEXT LINE		1 2 → NEXT LINE		
14	1 2 → NEXT LINE		1 2 → NEXT LINE		
15	1 2 → NEXT LINE		1 2 → NEXT LINE		
16	1 2 → NEXT LINE		1 2 → NEXT LINE		
17	1 2 → NEXT LINE		1 2 → NEXT LINE		
18	1 2 → NEXT LINE		1 2 → NEXT LINE		
19	1 2 → NEXT LINE		1 2 → NEXT LINE		
20	1 2 → NEXT LINE		1 2 → NEXT LINE		

CODES FOR Qs. 17 AND 19: EDUCATION

**LEVEL**	**GRADE**
1 = PRIMARY	00 = LESS THAN 1 YEAR COMPLETED
2 = SECONDARY	(USE '00' FOR Q. 17 ONLY.
3 = HIGHER	THIS CODE IS NOT ALLOWED
6 = PRE-PRIMARY	FOR Q. 19)
8 = DON'T KNOW	98 = DON'T KNOW

HOUSEHOLD CHARACTERISTICS

NO.	QUESTIONS AND FILTERS	CODING CATEGORIES	SKIP
102	What is the main source of drinking water for members of your household?	PIPED WATER<br>PIPED INTO DWELLING . . . . 11<br>PIPED TO YARD/PLOT . . . . 12<br>PUBLIC TAP/STANDPIPE . . . . 13<br>TUBE WELL OR BOREHOLE . . . . 21<br>DUG WELL<br>PROTECTED WELL . . . . 31<br>UNPROTECTED WELL . . . . 32<br>WATER FROM SPRING<br>PROTECTED SPRING . . . . 41<br>UNPROTECTED SPRING . . . . 42<br>RAINWATER . . . . 51<br>TANKER TRUCK . . . . 61<br>CART WITH SMALL TANK . . . . 71<br>SURFACE WATER (RIVER/DAM/<br>LAKE/POND/STREAM/CANAL/<br>IRRIGATION CHANNEL) . . . . 81<br>BOTTLED WATER . . . . 91<br><br>OTHER ________________ 96<br>(SPECIFY)	11, 12 → 105<br><br>51 → 105
107	What kind of toilet facility do members of your household usually use? **(3)**	FLUSH OR POUR FLUSH TOILET<br>FLUSH TO PIPED SEWER<br>SYSTEM . . . . 11<br>FLUSH TO SEPTIC TANK . . . . 12<br>FLUSH TO PIT LATRINE . . . . 13<br>FLUSH TO SOMEWHERE ELSE . . . . 14<br>FLUSH, DON'T KNOW WHERE . . . . 15<br>PIT LATRINE<br>VENTILATED IMPROVED<br>PIT LATRINE . . . . 21<br>PIT LATRINE WITH SLAB . . . . 22<br>PIT LATRINE WITHOUT SLAB/<br>OPEN PIT . . . . 23<br>COMPOSTING TOILET . . . . 31<br>BUCKET TOILET . . . . 41<br>HANGING TOILET/HANGING<br>LATRINE . . . . 51<br>NO FACILITY/BUSH/FIELD . . . . 61<br><br>OTHER ________________ 96<br>(SPECIFY)	61 → 110
108	Do you share this toilet facility with other households?	YES . . . . 1<br>NO 2	2 → 110
109	How many households use this toilet facility?	NO. OF HOUSEHOLDS<br>IF LESS THAN 10 . . . . [0 |]<br><br>10 OR MORE HOUSEHOLDS . . . . 95<br>DON'T KNOW 98	

NO.	QUESTIONS AND FILTERS	CODING CATEGORIES	SKIP
110	Does your household have: **(4)**	YES NO	
	Electricity?	ELECTRICITY 1 2	
	Koloboyi?	KOLOBOYI 1 2	
	A paraffin lamp other than a koloboyi?	PARAFFIN LAMP 1 2	
	A radio?	RADIO 1 2	
	A television?	TELEVISION 1 2	
	A cellular phone?	CELL PHONE 1 2	
	A telephone (landline)?	TELEPHONE (LANDLINE) . . . 1 2	
	A bed with mattress?	BED WITH MATTRESS 1 2	
	A sofa set?	SOFA SET 1 2	
	A refrigerator?	REFRIGERATOR 1 2	
	A dinner table?	DINNER TABLE 1 2	
	Chairs?	CHAIRS 1 2	
	A cabinet/cupboard?	CABINET/CUPBOARD 1 2	
	A stove?	STOVE 1 2	
	A washing machine?	WASHING MACHINE 1 2	
	A fan/air conditioner?	FAN/AIR CONDITIONER 1 2	
	A generator?	GENERATOR 1 2	
	A computer?	COMPUTER 1 2	
	A VCR?	VCR 1 2	
	A CD/cassette player?	CD/CASSETTE PLAYER 1 2	
	A camera?	CAMERA 1 2	
	[ADD ADDITIONAL ITEMS. SEE FOOTNOTE 4.]		
111	What type of fuel does your household mainly use for cooking?	ELECTRICITY . . . . 01<br>LPG . . . . 02<br>NATURAL GAS . . . . 03<br>BIOGAS . . . . 04<br>KEROSENE . . . . 05<br>COAL, LIGNITE . . . . 06<br>CHARCOAL . . . . 07<br>WOOD . . . . 08<br>STRAW/SHRUBS/GRASS . . . . 09<br>AGRICULTURAL CROP . . . . 10<br>ANIMAL DUNG . . . . 11<br>NO FOOD COOKED<br>IN HOUSEHOLD . . . . 95<br>OTHER ______________________ 96<br>(SPECIFY)	 <br><br><br><br><br><br><br><br><br><br><br>95 → 114
112	Is the cooking usually done in the house, in a separate building, or outdoors?	IN THE HOUSE . . . . 1<br>IN A SEPARATE BUILDING . . . . 2<br>OUTDOORS . . . . 3<br>OTHER ______________________ 6<br>(SPECIFY)	2, 3, 6 → 114
113	Do you have a separate room which is used as a kitchen?	YES . . . . 1<br>NO 2	
114	MAIN MATERIAL OF THE FLOOR. **(3)**<br>RECORD OBSERVATION.	NATURAL FLOOR<br>EARTH/SAND . . . . 11<br>DUNG . . . . 12<br>RUDIMENTARY FLOOR<br>WOOD PLANKS . . . . 21<br>PALM/BAMBOO . . . . 22<br>FINISHED FLOOR<br>PARQUET OR POLISHED<br>WOOD . . . . 31<br>VINYL OR ASPHALT STRIPS . . . . 32<br>CERAMIC TILES . . . . 33<br>CEMENT . . . . 34<br>CARPET . . . . 35<br>OTHER ______________________ 96<br>(SPECIFY)	

NO.	QUESTIONS AND FILTERS	CODING CATEGORIES	SKIP
115	MAIN MATERIAL OF THE ROOF. **(3)**<br><br>RECORD OBSERVATION.	NATURAL ROOFING<br>NO ROOF . . . . 11<br>THATCH/PALM LEAF . . . . 12<br>SOD . . . . 13<br>RUDIMENTARY ROOFING<br>RUSTIC MAT . . . . 21<br>PALM/BAMBOO . . . . 22<br>WOOD PLANKS . . . . 23<br>CARDBOARD . . . . 24<br>FINISHED ROOFING<br>METAL . . . . 31<br>LOCAL TILES . . . . 32<br>WOOD . . . . 33<br>CALAMINE/CEMENT FIBER . . . . 34<br>CERAMIC TILES . . . . 35<br>CEMENT . . . . 36<br>ROOFING SHINGLES . . . . 37<br><br>OTHER ______________ 96<br>(SPECIFY)	
116	MAIN MATERIAL OF THE EXTERIOR WALLS. **(3)**<br><br>RECORD OBSERVATION.	NATURAL WALLS<br>NO WALLS . . . . 11<br>CANE/PALM/TRUNKS . . . . 12<br>DIRT . . . . 13<br>RUDIMENTARY WALLS<br>BAMBOO WITH MUD . . . . 21<br>STONE WITH MUD . . . . 22<br>UNCOVERED ADOBE . . . . 23<br>PLYWOOD . . . . 24<br>CARDBOARD . . . . 25<br>REUSED WOOD . . . . 26<br>FINISHED WALLS<br>CEMENT . . . . 31<br>STONE WITH LIME/CEMENT . . . . 32<br>BRICKS . . . . 33<br>CEMENT BLOCKS . . . . 34<br>COVERED ADOBE . . . . 35<br>WOOD PLANKS/SHINGLES . . . . 36<br><br>OTHER ______________ 96<br>(SPECIFY)	
117	How many rooms in this household are used for sleeping?	ROOMS . . . . [ ][ ]	
118	Does any member of this household own:<br><br>A watch?<br>A bicycle?<br>A motorcycle or motor scooter?<br>An animal-drawn cart (oxcart)?<br>A car or truck?<br>A boat with a motor?	YES NO<br>WATCH . . . . 1 2<br>BICYCLE . . . . 1 2<br>MOTORCYCLE/SCOOTER . . . 1 2<br>ANIMAL-DRAWN CART . . . . . 1 2<br>CAR/TRUCK . . . . 1 2<br>BOAT WITH MOTOR 1 2	
119	Does any member of this household own any agricultural land?	YES . . . . 1<br>NO 2	 → 126
120	How much agricultural land do members of this household own?<br><br>RECORD IN UNITS RESPONDENT USES.	ACRES . . . . 1 [ ][ ].[ ]<br><br>HECTARES . . . . 2 [ ][ ].[ ]<br><br>FOOTBALL PITCHES . . . 3 [ ][ ].[ ]<br><br>95 OR MORE ACRES/HECTARES/FOOTBALL PITCHES . . . . 9995<br>DON'T KNOW 9998	

NO.	QUESTIONS AND FILTERS	CODING CATEGORIES	SKIP
126	Does any member of this household have a bank account?	YES . . . . 1<br>NO 2	
126A	At any time in the past 12 months, has anyone come into your house to spray the interior walls of your dwelling against mosquitoes?	YES . . . . 1<br>NO . . . . 2<br>DON'T KNOW 8	2, 8 → 127
126B	How many months ago was the house sprayed?<br><br>IF LESS THAN 1 MONTH AGO, RECORD '00'	MONTHS . . . . [ ][ ]	
126C	Who sprayed the house?	ARMY/POLICE . . . . 1<br>OTHER GOVERNMENT WORKER/<br>PROGRAMME . . . . 2<br>PRIVATE COMPANY . . . . 3<br><br>OTHER ____________ . . . . 6<br>(SPECIFY)<br>DON'T KNOW 8	
127	Does your household have any mosquito nets that can be used while sleeping?	YES . . . . 1<br>NO 2	2 → M1
128	How many mosquito nets does your household have?<br><br>IF 7 OR MORE NETS, RECORD '7'.	NUMBER OF NETS . . . . [ ]	

FOOTNOTES

(1) This section should be adapted for country-specific survey design.
(2) In Q. 18, the year should refer to the school year that is in session at the time the survey begins. If the survey begins between two school years, then the year should refer to the school year that just ended.
(3) Coding categories to be developed locally and revised based on the pretest; however, the broad categories must be maintained.
(4) Each country should add to the list at least five items of furniture (such as a table, a chair, a sofa, a bed, an armoire, or a cupboard or cabinet). In addition, each country should add at least four additional household appliances so that the list includes at least three items that even a poor household may have, at least three items that a middle income household may have, and at least three items that a high income household may have. Some possible additions are clock, water pump, grain grinder, fan, blender, water heater, generator, washing machine, microwave oven, computer, VCR or DVD player, cassette or CD player, camera air conditioner or cooler, color TV, sewing machine.
(5) Add other country-specific animals, such as oxen, water buffalo, camels, llamas, alpacas, pigs, ducks, geese or elephants.
(6) The question should be deleted in countries that do not have an organized spraying program to prevent the transmission of malaria.
(7) The question should be deleted in countries that are not affected by malaria.
(8) There are many different kinds of iodine testing kits available. The proper test kit should be selected in each country depending on the type of iodine additive used in the country (potassium iodate or potassium iodide). If both of these additives are used in a country, then both types of test kits should be used.
(9) Year of fieldwork is assumed to be 2010. For fieldwork beginning in 2011 or 2012, the year should be 2006 or 2007, respectively.
(10) In countries where the weighing scale shows the weight to only one decimal place, retain only one box after the decimal point and delete the first '9' from the other three codes.
(11) In countries where some enumeration areas are higher than 1,000 meters, altitude information should be collected on a separate form for each enumeration area higher than 1,000 meters so that the anemia estimat can be adjusted appropriately.
(12) Questions should be omitted in countries in which HIV testing is not a component of the survey.

HOUSEHOLD EXPENSES

This section will be answered by a person who is knowledgeable about the household's expenses.

Questions	AMOUNT IN MKW
**Clothing Expenses**	
**M1.** How much has the household spent in the last 30 days on :	
a. Cloth	|__|__|__|__|__|__|__|
b. Dresses	|__|__|__|__|__|__|__|
c. Shirts	|__|__|__|__|__|__|__|
d. Pants	|__|__|__|__|__|__|__|
e. Coats	|__|__|__|__|__|__|__|
f. Jackets	|__|__|__|__|__|__|__|
g. Sweaters/Woolwear	|__|__|__|__|__|__|__|
h. Cottonwear	|__|__|__|__|__|__|__|
i. Shoes	|__|__|__|__|__|__|__|
j. Making and/or repairing clothes and shoes	|__|__|__|__|__|__|__|
**Shelter Expenses**	
**M2.** How much has the household spent in the last 30 days on :	
a. Rent / Mortgage	|__|__|__|__|__|__|__|
b. House repairs	|__|__|__|__|__|__|__|
c. Purchase of equipment for the house (furniture, blankets, sheets, tablecloths, curtains, toiletries, etc.)	|__|__|__|__|__|__|__|
d. Water and electricity	
i. Water	|__|__|__|__|__|__|__|
ii. Electricity	|__|__|__|__|__|__|__|
e. Construction and maintenance of fences	|__|__|__|__|__|__|__|
**Expenses on Leisure Activities and Ceremonies**	
**M3.** How much has the household spent in the last 30 days on :	
a. Cinema/Theater	|__|__|__|__|__|__|__|
b. Sports	|__|__|__|__|__|__|__|
c. Lectures	|__|__|__|__|__|__|__|
d. Dowry, bride price (*lobola),* and marriage	|__|__|__|__|__|__|__|
e. Birth and baptism	|__|__|__|__|__|__|__|
f. Other various ceremonies (i.e., death, mourning, birthdays, etc.)	|__|__|__|__|__|__|__|
**Transportation and Communication Expenses**	
**M4.** How much has the household spent in the last 30 days on :	
a. Travel, including daily transportation, vacations, etc.	|__|__|__|__|__|__|__|
b. Purchase of fuel, lubricants, and maintenance	|__|__|__|__|__|__|__|
c. Telephone, Fax, Telex, Internet	|__|__|__|__|__|__|__|
d. Radio, TV	|__|__|__|__|__|__|__|
e. Postal correspondence fees	|__|__|__|__|__|__|__|
**Health and Education Expenditures**	
**M5.** How much has the household spent in the last 30 days on :	
a. Health expenses, including consultations, medications, exams, transport, hospitalization, insurance, etc.	|__|__|__|__|__|__|__|
b. Education expenses, including tuition, books, uniforms, transport, etc.	|__|__|__|__|__|__|__|
**Transfers and Other Expenditures**	
**M6.** How much has the household spent in the last 30 days on :	
a. Payments for domestic help, maids, nannies, watchmen	|__|__|__|__|__|__|__|
b. Transfers paid to other households	|__|__|__|__|__|__|__|
c. Council rates (i.e. Market fees)	|__|__|__|__|__|__|__|
d. Fines or legal fees	|__|__|__|__|__|__|__|
e. Losses due to theft	|__|__|__|__|__|__|__|
f. Other expenses	|__|__|__|__|__|__|__|

HOUSEHOLD EXPENSES (END) – CURRENT CONSUMPTION OF GOODS OVER THE PAST 7 DAYS

To be answered by the woman or a person who is knowledgeable about the household's purchases. The woman and the financially knowledgeable person may answer this section together.

NO.	GOODS	**M11**. In the last 7 days, did your household consume (NAME OF GOOD)?	**M12.** How much did you buy?		**M13**. Did you buy it?	**M14**. How much did you pay for it?
		1. Yes<br>2. No ➔ NEXT GOOD	Qty.	Unit	1. Yes<br>2. No ➔ NEXT GOOD	Amount (in MKW)
**CEREALS, GRAINS AND CEREAL PRODUCTS**						
1	Maize *ufa mgaiwa* (normal flour)	|__|		kg	|__|	|__|__|__|__|__|
2	Maize *ufa* refined (fine flour)	|__|		kg	|__|	|__|__|__|__|__|
3	Maize *ufa madeya* (bran flour)	|__|		kg	|__|	|__|__|__|__|__|
4	Maize grain (not as *ufa*)	|__|		kg	|__|	|__|__|__|__|__|
5	Green maize	|__|		kg	|__|	|__|__|__|__|__|
6	Rice	|__|		kg	|__|	|__|__|__|__|__|
7	Finger millet (*mawere*)	|__|		kg	|__|	|__|__|__|__|__|
8	Sorghum (*mapira*)	|__|		kg	|__|	|__|__|__|__|__|
9	Pearl millet (*mchewere*)	|__|		kg	|__|	|__|__|__|__|__|
10	Wheat flour	|__|		kg	|__|	|__|__|__|__|__|
11	Bread	|__|		kg	|__|	|__|__|__|__|__|
12	Buns, scones	|__|		kg	|__|	|__|__|__|__|__|
13	Biscuits	|__|		kg	|__|	|__|__|__|__|__|
14	Spaghetti, macaroni, pasta	|__|		kg	|__|	|__|__|__|__|__|
15	Breakfast cereal	|__|		kg	|__|	|__|__|__|__|__|
16	Infant feeding cereals	|__|		kg	|__|	|__|__|__|__|__|
**SUGAR, FATS, AND OILS**						
18	Sugar	|__|		kg	|__|	|__|__|__|__|__|
19	Sugar Cane	|__|		kg	|__|	|__|__|__|__|__|
20	Cooking oil	|__|		L	|__|	|__|__|__|__|__|
**ROOTS, TUBERS, AND PLANTAINS**						
22	Cassava tubers	|__|		kg	|__|	|__|__|__|__|__|
23	Cassava flour	|__|		kg	|__|	|__|__|__|__|__|

NO.	GOODS	M11. In the last 7 days, did your household consume (NAME OF GOOD)?	M12. How much did you buy?		M13. Did you buy it?	M14. How much did you pay for it?
		1. Yes<br>2. No ➔ NEXT GOOD	Qty.	Unit	1. Yes<br>2. No ➔ NEXT GOOD	Amount (in MKW)
24	White sweet potato			kg		
25	Orange sweet potato			kg		
26	Irish potato			kg		
27	Potato crisps			kg		
28	Plantain, cooking banana			kg		
29	Cocoyam (*masimbi*)			kg		
**NUTS AND PULSES**						
31	Bean, white			kg		
32	Bean, brown			kg		
33	Pigeonpea (*nandolo*)			kg		
34	Groundnut			kg		
35	Groundnut flour			kg		
36	Soyabean flour			kg		
37	Soya Pieces			kg		
38	Ground bean (*nzama)*			kg		
39	Cowpea (*khobwe*)			kg		
40	Macadamia nuts			kg		
41	Elephant Beans (Kalongonda)			kg		
**VEGETABLES**						
43	Onion			kg		
44	Cabbage			kg		
45	*Tanaposi* / Rape			kg		
46	*Nkhwani*			kg		
47	Chinese cabbage			kg		
48	Other cultivated green leafy vegetables			kg		
49	Gathered wild green leaves			kg		

NO.	GOODS	M11. In the last 7 days, did your household consume (NAME OF GOOD)?	M12. How much did you buy?		M13. Did you buy it?	M14. How much did you pay for it?
		1. Yes<br>2. No ➔ NEXT GOOD	Qty.	Unit	1. Yes<br>2. No ➔ NEXT GOOD	Amount (in MKW)
50	Tomato			kg		
51	Cucumber			kg		
52	Pumpkin			kg		
53	Okra / *Therere*			kg		
54	Tinned vegetables			kg		
55	Mushroom			kg		
**FRUITS**						
57	Mango			kg		
58	Banana			kg		
59	Citrus – naartje, orange, etc.			kg		
60	Pineapple			kg		
61	Papaya			kg		
62	Guava			kg		
63	Avocado			kg		
64	Wild fruit (*masau, malambe, etc.*)			kg		
65	Apple			kg		
**MEATS, FISH, AND ANIMAL PRODUCTS**						
67	Eggs			kg		
68	Dried fish			kg		
69	Fresh fish			kg		
70	Beef			kg		
71	Goat			No.		
72	Pork			kg		
73	Mutton			kg		
74	Chicken			kg		
75	Other poultry - guinea fowl, doves, etc.			kg		

NO.	GOODS	M11. In the last 7 days, did your household consume (NAME OF GOOD)?	M12. How much did you buy?		M13. Did you buy it?	M14. How much did you pay for it?
		1. Yes<br>2. No ➔ NEXT GOOD	Qty.	Unit	1. Yes<br>2. No ➔ NEXT GOOD	Amount (in MKW)
76	Small animal – rabbit, mice, etc.			kg		
77	Termites, other insects (eg Ngumbi, caterpillar)			kg		
78	Tinned meat or fish			kg		
79	Smoked fish			kg		
80	Fish Soup/Sauce			kg		
**COOKED FROM VENDOR**						
82	Maize - boiled or roasted (vendor)			kg		
83	Chips (vendor)			kg		
84	Cassava - boiled (vendor)			kg		
85	Eggs - boiled (vendor)			kg		
86	Chicken (vendor)			kg		
87	Meat (vendor)			kg		
88	Fish (vendor)			kg		
89	*Mandazi, zitumbuwa, chigumu or scones, biscuits*, doughnut (vendor)			kg		
90	Samosa (vendor), wampunga or wambates			kg		
91	Meal eaten at restaurant			No.		
**MILK AND MILK PRODUCTS**						
93	Fresh milk			L		
94	Powdered milk			kg		
95	Margarine - Blue band			kg		
96	Butter			kg		
97	*Chambiko* - soured milk			L		
98	Yoghurt			kg		
99	Cheese			kg		
100	Infant feeding formula (for bottle)			kg		
**BEVERAGES**						

NO.	GOODS	M11. In the last 7 days, did your household consume (NAME OF GOOD)?	M12. How much did you buy?		M13. Did you buy it?	M14. How much did you pay for it?
		1. Yes<br>2. No ➔ NEXT GOOD	Qty.	Unit	1. Yes<br>2. No ➔ NEXT GOOD	Amount (in MKW)
102	Tea			L		
103	Coffee			L		
104	Cocoa, Milo			L		
105	Squash (Sobo drink concentrate)			L		
106	Fruit juice			L		
107	Freezes (flavored ice)			L		
108	Soft drinks (Coca-cola, Fanta, Sprite, etc.)			L		
109	Chibuku (commercial traditional-style beer)			L		
110	Bottled water			L		
111	Maheu			L		
112	Bottled / canned beer (Carlsberg, etc.)			L		
113	Thobwa			L		
114	Traditional beer (*masese*)			L		
115	Wine or commercial liquor (gudugu, pineapple wine, kachaso, tyson)			L		
116	Locally brewed liquor (*kachasu*)			L		
**SPICES AND MISCELLANEOUS**						
117	Salt			kg		
118	Spices			kg		
119	Yeast, baking powder, bicarbonate of soda			kg		
120	Tomato sauce (bottle)			L		
121	Hot sauce (Nali, etc.)			kg		
122	Jam, jelly			kg		
123	Sweets, candy, chocolates			kg		
124	Honey			L		
**NON-FOOD ITEMS**						
126	Charcoal			kg		

NO.	GOODS	M11. In the last 7 days, did your household consume (NAME OF GOOD)?	M12. How much did you buy?		M13. Did you buy it?	M14. How much did you pay for it?
		1. Yes<br>2. No ➔ NEXT GOOD	Qty.	Unit	1. Yes<br>2. No ➔ NEXT GOOD	Amount (in MKW)
127	Paraffin or kerosene			No.		
128	Firewood			No. pieces		
129	Cigarettes or other tobacco			No.		
130	Candles			No.		
131	Matches			No. boxes		
132	Batteries			No.		
133	Newspapers or magazines			No.		
134	Public transport - Bicycle Taxi			No. tickets		
135	Public transport - Bus/Minibus			No. tickets		
136	Public transport - Other (Truck, Oxcart, Etc.)			No. tickets		
137	Soap bar			No.		
143	Church fees / Tithes			No. fees		
145	Telephone airtime			No. credits		

FOLLOW-UP INFORMATION

Thank you for participating in this section of the survey. We may contact your household again in a few years to learn more about how life changes for Malawian families. Could you please give us information about two people who **DO NOT LIVE IN THE HOUSEHOLD** and who would know where you or other household members are, or how to reach you, in the future?

CONTACT 1

FULL NAME: ________________________________

RELATIONSHIP TO YOU: ________________________________

FULL ADDRESS: ________________________________

____

____

PHONE NUMBER: ________________________________

E-MAIL ADDRESS: ________________________________

CONTACT 2

FULL NAME: ________________________________

RELATIONSHIP TO YOU: ________________________________

FULL ADDRESS: ________________________________

____

____

PHONE NUMBER: ________________________________

E-MAIL ADDRESS: ________________________________

OTHER HOUSEHOLD CONTACT:

What is the name and phone number of someone else within your household?

NAME: ________________________________

PHONE: ________________________________

RELATIONSHIP TO YOU: ________________________________

PLANS TO MOVE:

Does your family/household have any plans to move in the next two years? YES NO

IF YES: Where do you plan to move to? ADDRESS: ________________________________

____

____

PHOTO OF HOUSEHOLD:

With your permission, I would now like to take a photo of the front of your house.
Taking this photo will help us to find your house again in the future.

May I take a photo of the front of your house?	YES	NO

IF YES, PLEASE PROCEED TO THE EXTERIOR OF THE HOUSE AND TAKE A PHOTO OF THE FRONT OF THE HOUSE.

PHOTO OF RESPONDENT:

With your permission, I would now like to take a photo of you. Taking your photo will help us to find you again in the future.

May I take your photo now?	YES	NO

IF YES, PLEASE TAKE A PHOTO OF THE RESPONDENT. BE SURE TO CLEARLY CAPTURE THE RESPONDENT'S FACE FROM THE NECK UP.

END OF HOUSEHOLD SECTION:
You have now reached the end of the household section of the survey. We will now proceed to the woman's section of the survey. Thank you for your time and ideas. This has been extremely helpful. I want to remind you that all of your responses will remain confidential. I also ask that you not share the details of what was said here.

How does that sound to you? Do you have any further questions for me at this time?

Thank you again for your help.

END OF HOUSEHOLD SECTION. PROCEED TO WOMAN'S SECTION.

DATE ____________________

QUANTITATIVE SURVEY
WOMAN'S QUESTIONNAIRE
MALAWI FAMILY PLANNING SURVEY - WAVE I, 2016
HARVARD UNIVERSITY, IPA MALAWI

IDENTIFICATION (1)

NAME AND NO. OF THE DISTRICT ____________________

AREA . . . .

SECTOR . . . .

CLUSTER NUMBER . . . .

HOUSEHOLD ID NUMBER . . . .

NAME OF HOUSEHOLD HEAD ____________________

PRIMARY PHONE NO. OF HH HEAD ____________________

NAME AND LINE NUMBER OF WOMAN ____________________

IS THE WOMAN WHO IS ANSWERING THE SAME PERSON WHO ANSWERED THE HOUSEHOLD SECTION? Y N

IF YES, SKIP FOLLOWING THREE QUESTIONS ON PHONE NUMBER AND E-MAIL INFORMATION.

PRIMARY PHONE NO. OF WOMAN ____________________

ALTERNATE PHONE NO. OF WOMAN ____________________

E-MAIL OF WOMAN ____________________

INTERVIEWER VISITS				
	1	2	3	FINAL VISIT
DATE				DAY
				MONTH
				YEAR
INTERVIEWER'S NAME				INT. NUMBER
RESULT*				RESULT
NEXT VISIT: DATE				TOTAL NUMBER OF VISITS
TIME				

*RESULT CODES:

1	COMPLETED	4	REFUSED		
2	NOT AT HOME	5	PARTLY COMPLETED	7	OTHER (SPECIFY)
3	POSTPONED	6	INCAPACITATED		

COUNTRY-SPECIFIC INFORMATION: LANGUAGE OF QUESTIONNAIRE, LANGUAGE OF INTERVIEW, NATIVE LANGUAGE OF RESPONDENT, AND WHETHER TRANSLATOR USED

FIELD MANAGER	INTERVIEWER	OFFICE EDITOR	KEYED BY
NAME	NAME		

(1) This section should be adapted for country-specific survey design.

Note: Questions with blue highlighting in the question number column are HIV related questions that may be deletec in some circumstances (see footnotes). Questions with pink highlighting in the question number column are malaria related questions that may be deleted in some circumstances (see footnotes). Questions with yellow highlighting in the question number column are other questions that may be deleted in some circumstances (see footnotes).

SECTION 1. RESPONDENT'S BACKGROUND

INTRODUCTION

In this section, I will ask you questions about your health and well-being. The questions in this section usually take about 60 minutes. All of the answers you give will be confidential and will not be shared with anyone other than members of our survey team. You don't have to be in the survey, but we hope you will agree to answer the questions since your views are important. If I ask you any question you don't want to answer, just let me know and I will go on to the next question or you can stop the interview at any time.

In case you need more information about the survey, you may contact the person listed on the card that has already been given to your household.
Do you have any questions? May I begin the interview now?

NO.	QUESTIONS AND FILTERS	CODING CATEGORIES	SKIP
101	RECORD THE TIME.	HOUR . . . . [ ][ ]<br>MINUTES . . . . [ ][ ]	
102	In what month and year were you born?	MONTH . . . . [ ][ ]<br>DON'T KNOW MONTH . . . . 98<br>YEAR . . . . [ ][ ][ ][ ]<br>DON'T KNOW YEAR 9998	
103	How old were you at your last birthday?<br><br>COMPARE AND CORRECT 102 AND/OR 103 IF INCONSISTENT.	AGE IN COMPLETED YEARS [ ][ ]	
104	Have you ever attended school?	YES . . . . 1<br>NO 2	 → 108
105	What is the highest level of school you attended: primary, secondary, or higher? (1)	PRIMARY . . . . 1<br>SECONDARY . . . . 2<br>HIGHER 3	
106	What is the highest (class/form/year) you completed at that level? (1)<br><br>IF COMPLETED LESS THAN ONE YEAR AT THAT LEVEL, RECORD '00'.	CLASS/FORM/YEAR . . . . [ ][ ]	
107	CHECK 105 (SCHOOL CODE):<br>PRIMARY [ ] ↓ SECONDARY OR HIGHER [ ]		→ 113

NO.	QUESTIONS AND FILTERS	CODING CATEGORIES	SKIP
108	Now I would like you to read this sentence to me.<br><br>SHOW CARD TO RESPONDENT. **(2)**<br>IF RESPONDENT SPEAKS MULTIPLE LANGUAGES, SELECT ALL THAT APPLIES.<br><br>IF RESPONDENT CANNOT READ WHOLE SENTENCE, PROBE: Can you read any part of the sentence to me?	**ENGLISH**<br>CANNOT READ AT ALL . . . . 1<br>ABLE TO READ ONLY PARTS OF SENTENCE . . . . 2<br>ABLE TO READ WHOLE SENTENCE 3<br><br>**CHICHEWA**<br>CANNOT READ AT ALL . . . . 4<br>ABLE TO READ ONLY PARTS OF SENTENCE . . . . 5<br>ABLE TO READ WHOLE SENTENCE 6<br><br>**TUMBUKA**<br>CANNOT READ AT ALL . . . . 7<br>ABLE TO READ ONLY PARTS OF SENTENCE . . . . 8<br>ABLE TO READ WHOLE SENTENCE 9<br><br>NO CARD WITH REQUIRED LANGUAGE____________________ 21<br>(SPECIFY LANGUAGE)<br>BLIND/VISUALLY IMPAIRED 31	
109	Have you ever participated in a literacy program or any other program that involves learning to read or write (not including primary school)?	YES . . . . 1<br>NO 2	
113	What is your religion?	CATHOLIC . . . . 1<br>CCAP . . . . 2<br>ANGLICAN . . . . 3<br>SEVENTH DAY ADVENTIST / BAPTIST 4<br>OTHER CHRISTIAN . . . . 5<br>MUSLIM . . . . 6<br>NO RELIGION . . . . 7<br>OTHER________________________ 96<br>(SPECIFY)	
114	What is your tribe or ethnic group?	CHEWA . . . . 1<br>TUMBUKA . . . . 2<br>LOMWE . . . . 3<br>TONGA . . . . 4<br>YAO . . . . 5<br>SENA . . . . 6<br>NKHONDE . . . . 7<br>NGONI . . . . 8<br>OTHER________________________ 96<br>(SPECIFY)	

(1) Revise according to the local education system.
(2) Each card should have four simple sentences appropriate to the country (e.g., "Parents love their children.", "Farming is hard work.", "The child is reading a book.", "Children work hard at school."). Cards should be prepared for every language in which respondents are likely to be literate.
(3) The question may be considered for deletion in countries with a very low HIV prevalence.

CODES FOR 106: HIGHEST EDUCATION LEVEL ATTAINED

Description	Code
Some schooling but not Completed Primary (Ecole Primaire) 1st Year (P.1)	10
Completed P.1	11
Completed P.2	12
Completed P.3	13
Completed P.4	14
Completed P.5	15
Completed P.6	16
Completed Middle/College 1st Year (M.1) (Ecole Secondaire, 1er Cycle)	21
Completed M.2	22
Completed M.3	23
Completed M.4	24
Completed Secondary/Lycee General (S.1) (Ecole Secondaire, 2eme Cycle)	31
Completed S.2	32
Completed S.3	33
Completed Vocational/Enseignants du primaire (V.1)(Superieur)	41
Completed V.2	42
Completed V.3	43
Completed V.4	44
Completed Premier cycle universitaire	51
Completed Enseignant secondaire	61
Completed Degree and above (2eme, 3eme cycle universitaire)	71
Don't Know	99

NO.	QUESTIONS AND FILTERS	CODING CATEGORIES	SKIP
201	Now I would like to ask about all the births you have had during your life. Have you ever given birth?	YES . . . . 1<br>NO 2	 → 206
202	Do you have any sons or daughters to whom you have given birth who are now living with you?	YES . . . . 1<br>NO 2	 → 204
203	How many sons live with you?<br><br>And how many daughters live with you?<br><br>IF NONE, RECORD '00'.	SONS AT HOME . . . . [ ][ ]<br><br>DAUGHTERS AT HOME . . . . . [ ][ ]	
204	Do you have any sons or daughters to whom you have given birth who are alive but do not live with you?	YES . . . . 1<br>NO 2	 → 206
205	How many sons are alive but do not live with you?<br><br>And how many daughters are alive but do not live with you?<br><br>IF NONE, RECORD '00'.	SONS ELSEWHERE . . . . [ ][ ]<br><br>DAUGHTERS ELSEWHERE [ ][ ]	
206	Have you ever given birth to a boy or girl who was born alive but later died?<br><br>IF NO, PROBE: Any baby who cried or showed signs of life but did not survive?	YES . . . . 1<br>NO 2	 → 208
207	How many boys have died?<br><br>And how many girls have died?<br><br>IF NONE, RECORD '00'.	BOYS DEAD . . . . [ ][ ]<br><br>GIRLS DEAD . . . . [ ][ ]	
208	SUM ANSWERS TO 203, 205, AND 207, AND ENTER TOTAL.<br>IF NONE, RECORD '00'.	TOTAL BIRTHS . . . . [ ][ ]	
209	CHECK 208:<br><br>Just to make sure that I have this right: you have had in TOTAL _____ births during your life. Is that correct?<br><br>YES [ ] ↓ NO [ ] → PROBE AND CORRECT 201-208 AS NECESSARY.		
210	CHECK 208:<br><br>ONE OR MORE BIRTHS [ ] ↓ NO BIRTHS [ ] →		226

211 Now I would like to record the names of all your births, whether still alive or not, starting with the first one you had.
RECORD NAMES OF ALL THE BIRTHS IN 212. RECORD TWINS AND TRIPLETS ON SEPARATE ROWS.
(IF THERE ARE MORE THAN 12 BIRTHS, USE AN ADDITIONAL QUESTIONNAIRE, STARTING WITH THE SECOND ROW).

212<br>What name was given to your (first/next) baby?<br>RECORD NAME.<br>BIRTH HISTORY NUMBER	213<br>Is (NAME) a boy or a girl?	214<br>Were any of these births twins?	215<br>In what month and year was (NAME) born?<br>PROBE: When is his/her birthday?	216<br>Is (NAME) still alive?	217<br>IF ALIVE:<br>How old was (NAME) at his/her last birthday?<br>RECORD AGE IN COM-PLETED YEARS.	218<br>IF ALIVE:<br>Is (NAME) living with you?	219<br>IF ALIVE:<br>RECORD HOUSE-HOLD LINE NUMBER OF CHILD (RECORD '00' IF CHILD NOT LISTED IN HOUSE-HOLD).	220<br>IF DEAD:<br>How old was (NAME) when he/she died?<br>IF '1 YR', PROBE: How many months old was (NAME)? RECORD DAYS IF LESS THAN 1 MONTH; MONTHS IF LESS THAN TWO YEARS; OR YEARS.	221<br>Were there any other live births between (NAME OF PREVIOUS BIRTH) and (NAME), including any children who died after birth?
01	BOY 1<br>GIRL 2	SING 1<br>MULT 2	MONTH<br>YEAR	YES . . 1<br>NO . . . 2<br>↓<br>220	AGE IN YEARS	YES . . . 1<br>NO 2	HOUSEHOLD LINE NUMBER<br>↓<br>(NEXT BIRTH)	DAYS . . . 1<br>MONTHS 2<br>YEARS . . 3	
02	BOY 1<br>GIRL 2	SING 1<br>MULT 2	MONTH<br>YEAR	YES . . 1<br>NO . . . 2<br>↓<br>220	AGE IN YEARS	YES . . . 1<br>NO 2	HOUSEHOLD LINE NUMBER<br>↓<br>(GO TO 221)	DAYS . . . 1<br>MONTHS 2<br>YEARS . . 3	YES . . . . 1<br>ADD BIRTH<br>NO . . . . . 2<br>NEXT BIRTH
03	BOY 1<br>GIRL 2	SING 1<br>MULT 2	MONTH<br>YEAR	YES . . 1<br>NO . . . 2<br>↓<br>220	AGE IN YEARS	YES . . . 1<br>NO 2	HOUSEHOLD LINE NUMBER<br>↓<br>(GO TO 221)	DAYS . . . 1<br>MONTHS 2<br>YEARS . . 3	YES . . . . 1<br>ADD BIRTH<br>NO . . . . . 2<br>NEXT BIRTH
04	BOY 1<br>GIRL 2	SING 1<br>MULT 2	MONTH<br>YEAR	YES . . 1<br>NO . . . 2<br>↓<br>220	AGE IN YEARS	YES . . . 1<br>NO 2	HOUSEHOLD LINE NUMBER<br>↓<br>(GO TO 221)	DAYS . . . 1<br>MONTHS 2<br>YEARS . . 3	YES . . . . 1<br>ADD BIRTH<br>NO . . . . . 2<br>NEXT BIRTH
05	BOY 1<br>GIRL 2	SING 1<br>MULT 2	MONTH<br>YEAR	YES . . 1<br>NO . . . 2<br>↓<br>220	AGE IN YEARS	YES . . . 1<br>NO 2	HOUSEHOLD LINE NUMBER<br>↓<br>(GO TO 221)	DAYS . . . 1<br>MONTHS 2<br>YEARS . . 3	YES . . . . 1<br>ADD BIRTH<br>NO . . . . . 2<br>NEXT BIRTH
06	BOY 1<br>GIRL 2	SING 1<br>MULT 2	MONTH<br>YEAR	YES . . 1<br>NO . . . 2<br>↓<br>220	AGE IN YEARS	YES . . . 1<br>NO 2	HOUSEHOLD LINE NUMBER<br>↓<br>(GO TO 221)	DAYS . . . 1<br>MONTHS 2<br>YEARS . . 3	YES . . . . 1<br>ADD BIRTH<br>NO . . . . . 2<br>NEXT BIRTH
07	BOY 1<br>GIRL 2	SING 1<br>MULT 2	MONTH<br>YEAR	YES . . 1<br>NO . . . 2<br>↓<br>220	AGE IN YEARS	YES . . . 1<br>NO 2	HOUSEHOLD LINE NUMBER<br>↓<br>(GO TO 221)	DAYS . . . 1<br>MONTHS 2<br>YEARS . . 3	YES . . . . 1<br>ADD BIRTH<br>NO . . . . . 2<br>NEXT BIRTH

212<br>What name was given to your next baby?<br>RECORD NAME.<br>BIRTH HISTORY NUMBER	213<br>Is (NAME) a boy or a girl?	214<br>Were any of these births twins?	215<br>In what month and year was (NAME) born?<br>PROBE: When is his/her birthday?	216<br>Is (NAME) still alive?	217<br>IF ALIVE:<br>How old was (NAME) at his/her last birthday?<br>RECORD AGE IN COMPLETED YEARS.	218<br>IF ALIVE:<br>Is (NAME) living with you?	219<br>IF ALIVE:<br>RECORD HOUSEHOLD LINE NUMBER OF CHILD (RECORD '00' IF CHILD NOT LISTED IN HOUSEHOLD).	220<br>IF DEAD:<br>How old was (NAME) when he/she died?<br>IF '1 YR', PROBE: How many months old was (NAME)? RECORD DAYS IF LESS THAN 1 MONTH; MONTHS IF LESS THAN TWO YEARS; OR YEARS.	221<br>Were there any other live births between (NAME OF PREVIOUS BIRTH) and (NAME), including any children who died after birth?
08	BOY 1<br>GIRL 2	SING 1<br>MULT 2	MONTH<br>YEAR	YES . . 1<br>NO . . . 2 → 220	AGE IN YEARS	YES . . . 1<br>NO 2	HOUSEHOLD LINE NUMBER<br>(GO TO 221)	DAYS . . . 1<br>MONTHS 2<br>YEARS . . 3	YES . . . . 1 → ADD BIRTH<br>NO . . . . . 2 → NEXT BIRTH
09	BOY 1<br>GIRL 2	SING 1<br>MULT 2	MONTH<br>YEAR	YES . . 1<br>NO . . . 2 → 220	AGE IN YEARS	YES . . . 1<br>NO 2	HOUSEHOLD LINE NUMBER<br>(GO TO 221)	DAYS . . . 1<br>MONTHS 2<br>YEARS . . 3	YES . . . . 1 → ADD BIRTH<br>NO . . . . . 2 → NEXT BIRTH
10	BOY 1<br>GIRL 2	SING 1<br>MULT 2	MONTH<br>YEAR	YES . . 1<br>NO . . . 2 → 220	AGE IN YEARS	YES . . . 1<br>NO 2	HOUSEHOLD LINE NUMBER<br>(GO TO 221)	DAYS . . . 1<br>MONTHS 2<br>YEARS . . 3	YES . . . . 1 → ADD BIRTH<br>NO . . . . . 2 → NEXT BIRTH
11	BOY 1<br>GIRL 2	SING 1<br>MULT 2	MONTH<br>YEAR	YES . . 1<br>NO . . . 2 → 220	AGE IN YEARS	YES . . . 1<br>NO 2	HOUSEHOLD LINE NUMBER<br>(GO TO 221)	DAYS . . . 1<br>MONTHS 2<br>YEARS . . 3	YES . . . . 1 → ADD BIRTH<br>NO . . . . . 2 → NEXT BIRTH
12	BOY 1<br>GIRL 2	SING 1<br>MULT 2	MONTH<br>YEAR	YES . . 1<br>NO . . . 2 → 220	AGE IN YEARS	YES . . . 1<br>NO 2	HOUSEHOLD LINE NUMBER<br>(GO TO 221)	DAYS . . . 1<br>MONTHS 2<br>YEARS . . 3	YES . . . . 1 → ADD BIRTH<br>NO . . . . . 2 → NEXT BIRTH

222	Have you had any live births since the birth of (NAME OF LAST BIRTH)? IF YES, RECORD BIRTH(S) IN TABLE.	YES . . . . 1<br>NO 2	
223	COMPARE 208 WITH NUMBER OF BIRTHS IN HISTORY ABOVE AND MARK:<br>NUMBERS ARE SAME ↓ NUMBERS ARE DIFFERENT → (PROBE AND RECONCILE)		
224	CHECK 215:<br>ENTER THE NUMBER OF BIRTHS IN JANUARY 2016 **(1)** OR LATER.	NUMBER OF BIRTHS . . . .<br>NONE 0	→ 226

NO.	QUESTIONS AND FILTERS	CODING CATEGORIES	SKIP
225	**C** FOR EACH BIRTH SINCE JANUARY 2016 **(1)**, ENTER 'B' IN THE MONTH OF BIRTH IN THE CALENDAR. WRITE THE NAME OF THE CHILD TO THE LEFT OF THE 'B' CODE. FOR EACH BIRTH, ASK THE NUMBER OF MONTHS THE PREGNANCY LASTED AND RECORD 'P' IN EACH OF THE PRECEDING MONTHS ACCORDING TO THE DURATION OF PREGNANCY. (NOTE: THE NUMBER OF 'P's MUST BE ONE LESS THAN THE NUMBER OF MONTHS THAT THE PREGNANCY LASTED.)		
226	Are you pregnant now?	YES . . . . 1<br>NO . . . . 2<br>UNSURE 8	 2, 8 → 230
227	How many months pregnant are you?<br><br>RECORD NUMBER OF COMPLETED MONTHS.<br><br>**C** ENTER 'P's IN THE CALENDAR, BEGINNING WITH THE MONTH OF INTERVIEW AND FOR THE TOTAL NUMBER OF COMPLETED MONTHS.	MONTHS . . . . [ ][ ]	
228	When you got pregnant, did you want to get pregnant at that time?	YES . . . . 1<br>NO 2	1 → 230
229	Did you want to have a baby later on or did you not want any (more) children?	LATER . . . . 1<br>NO MORE 2	
230	Have you ever had a pregnancy that miscarried, was aborted, or ended in a stillbirth?	YES . . . . 1<br>NO 2	 2 → 302
231	When did the last such pregnancy end?	MONTH . . . . [ ][ ]<br>YEAR . . . . . . [ ][ ][ ][ ]	

(1) Year of fieldwork is assumed to be 2010. For fieldwork beginning in 2011 or 2012, the year should be 2006 or 2007, respectively.

NO.	QUESTIONS AND FILTERS	CODING CATEGORIES	SKIP
232 (1)	CHECK 231: LAST PREGNANCY ENDED AFTER JANUARY 2016 ☐		→ 234
	LAST PREGNANCY ENDED IN 2015 OR EARLIER ☐		→ 302

LINE NO.	233 In what month and year did the preceding such pregnancy end?	234 How many months pregnant were you when that pregnancy ended?	235 (1) Since January 2016, have you had any other pregnancies that did not result in a live birth?	
01		NUMBER OF MONTHS	YES . . . . 1<br>NO 2	→ NEXT LINE<br>→ 236
02	MONTH YEAR	NUMBER OF MONTHS	YES . . . . 1<br>NO 2	→ NEXT LINE<br>→ 236
03	MONTH YEAR	NUMBER OF MONTHS	YES . . . . 1<br>NO 2	→ NEXT LINE<br>→ 236
04	MONTH YEAR	NUMBER OF MONTHS	YES . . . . 1<br>NO 2	→ 236

236 (1)	**C** FOR EACH PREGNANCY THAT DID NOT END IN A LIVE BIRTH IN JANUARY 2016 OR LATER, ENTER 'T' IN THE CALENDAR IN THE MONTH THAT THE PREGNANCY TERMINATED AND 'P' FOR THE REMAINING NUMBER OF COMPLETED MONTHS OF PREGNANCY.<br><br>IF THERE ARE MORE THAN FOUR PREGNANCIES THAT DID NOT END IN A LIVE BIRTH, USE AN ADDITIONAL QUESTIONNAIRE STARTING ON THE SECOND LINE.	

(1) Year of fieldwork is assumed to be 2010. For fieldwork beginning in 2011 or 2012, the year should be 2006 or 2007, respectively.

NO.	QUESTIONS AND FILTERS	CODING CATEGORIES	SKIP
302	CHECK 226:<br>NOT PREGNANT OR UNSURE ☐ (continue)<br>PREGNANT ☐ → 311		→ 311
303	Are you currently doing something or using any method to delay or avoid getting pregnant?	YES . . . . 1<br>NO 2	 → 311
304	Which method are you using? **(4)**<br><br>CIRCLE ALL MENTIONED.<br><br>IF MORE THAN ONE METHOD MENTIONED, FOLLOW SKIP INSTRUCTION FOR HIGHEST METHOD IN LIST.	MALE STERILIZATION . . . . B<br>IUD . . . . C<br>INJECTABLES . . . . D<br>IMPLANTS . . . . E<br>PILL . . . . F<br>CONDOM . . . . G<br>FEMALE CONDOM . . . . H<br>DIAPHRAGM . . . . I<br>FOAM/JELLY . . . . J<br>STANDARD DAYS METHOD . . . . K<br>LACTATIONAL AMEN. METHOD . . . . . L<br>RHYTHM METHOD . . . . M<br>WITHDRAWAL . . . . N<br>OTHER MODERN METHOD . . . . X<br>OTHER TRADITIONAL METHOD . . . Y	B → 308<br>C, D, E → 308A<br>G → 306<br>H–Y → 308A
305	What is the brand name of the pills you are using?<br><br>IF DON'T KNOW THE BRAND, ASK TO SEE THE PACKAGE.	LOFEMINOL 01<br>MICROGYNON 02<br>OVRETTE . . . . 03<br><br>OTHER ______ 96<br>(SPECIFY)<br>DON'T KNOW 98	→ 308A
306	What is the brand name of the condoms you are using?<br><br>IF DON'T KNOW THE BRAND, ASK TO SEE THE PACKAGE.	CHISHANGO . . . . 01<br>MANYUCHI . . . . 02<br>CARE (FEMALE CONDOM) . . . . 03<br><br>OTHER ______ 96<br>(SPECIFY)<br><br>DON'T KNOW 98	→ 308A
308<br><br>308A	In what month and year was the sterilization performed?<br><br>Since what month and year have you been using (CURRENT METHOD) without stopping?<br><br>PROBE: For how long have you been using (CURRENT METHOD) now without stopping?	MONTH . . . . ☐☐<br>YEAR . . . . ☐☐☐☐	

NO.	QUESTIONS AND FILTERS	CODING CATEGORIES	SKIP
309	CHECK 308/308A, 215 AND 231:<br>ANY BIRTH OR PREGNANCY TERMINATION AFTER MONTH AND YEAR OF START OF USE OF CONTRACEPTION IN 308/308A<br>YES ☐ NO ☐<br>YES → GO BACK TO 308/308A, PROBE AND RECORD MONTH AND YEAR AT START OF CONTINUOUS USE OF CURRENT METHOD (MUST BE AFTER LAST BIRTH OR PREGNANCY TERMINATION).		
310	CHECK 308/308A:<br>JANUARY 2016 **(6)** OR LATER ☐<br>C ENTER CODE FOR METHOD USED IN MONTH OF INTERVIEW IN THE CALENDAR AND IN EACH MONTH BACK TO THE DATE STARTED USING.<br>YEAR IS 2015 **(7)** OR EARLIER ☐<br>C ENTER CODE FOR METHOD USED IN MONTH OF INTERVIEW IN THE CALENDAR AND EACH MONTH BACK TO JANUARY 2016 **(6)**.<br>THEN SKIP TO →		322
311	I would like to ask you some questions about the times you or your partner may have used a method to avoid getting pregnant during the last year.<br>USE CALENDAR TO PROBE FOR EARLIER PERIODS OF USE AND NONUSE, STARTING WITH MOST RECENT USE, BACK TO JANUARY 2016. **(6)**<br>USE NAMES OF CHILDREN, DATES OF BIRTH, AND PERIODS OF PREGNANCY AS REFERENCE POINTS.<br>C **IN COLUMN 1**, ENTER METHOD USE CODE OR '0' FOR NONUSE IN EACH BLANK MONTH.<br>ILLUSTRATIVE QUESTIONS:<br>* When was the last time you used a method? Which method was that?<br>* When did you start using that method? How long after the birth of (NAME)?<br>* How long did you use the method then?<br>**IN COLUMN 2**, ENTER CODES FOR DISCONTINUATION NEXT TO THE LAST MONTH OF USE. NUMBER OF CODES IN COLUMN 2 MUST BE SAME AS NUMBER OF INTERRUPTIONS OF METHOD USE IN COLUMN 1.<br>ASK WHY SHE STOPPED USING THE METHOD. IF A PREGNANCY FOLLOWED, ASK WHETHER SHE BECAME PREGNANT UNINTENTIONALLY WHILE USING THE METHOD OR DELIBERATELY STOPPED TO GET PREGNANT.<br>ILLUSTRATIVE QUESTIONS:<br>* Why did you stop using the (METHOD)? Did you become pregnant while using (METHOD), or did you stop to get pregnant, or did you stop for some other reason?<br>* IF DELIBERATELY STOPPED TO BECOME PREGNANT, ASK: How many months did it take you to get pregnant after you stopped using (METHOD)? AND ENTER ‘0’ IN EACH SUCH MONTH IN COLUMN 1.		

NO.	QUESTIONS AND FILTERS	CODING CATEGORIES	SKIP
312	CHECK THE CALENDAR FOR USE OF ANY CONTRACEPTIVE METHOD IN ANY MONTH<br><br>NO METHOD USED ☐ ↓ ANY METHOD USED ☐ → 316		316
313	Have you ever used anything or tried in any way to delay or avoid getting pregnant?	YES . . . . . 1<br>NO 2	 → 324A
314	Which method(s) have you ever used? **(4)**<br><br>CIRCLE ALL MENTIONED.<br><br>IF MORE THAN ONE METHOD MENTIONED, FOLLOW SKIP INSTRUCTION FOR HIGHEST METHOD IN LIST.	MALE STERILIZATION . . . . . B<br>IUD . . . . . C<br>INJECTABLES . . . . . D<br>IMPLANTS . . . . . E<br>PILL . . . . . F<br>CONDOM . . . . . G<br>FEMALE CONDOM . . . . . H<br>DIAPHRAGM . . . . . I<br>FOAM/JELLY . . . . . J<br>STANDARD DAYS METHOD . . . . . K<br>LACTATIONAL AMEN. METHOD . . . . . L<br>RHYTHM METHOD . . . . . M<br>WITHDRAWAL . . . . . N<br>OTHER MODERN METHOD . . . . . X<br>OTHER TRADITIONAL METHOD . . . Y	→ 324A
316	CHECK 304:<br><br>CIRCLE METHOD CODE:<br><br>IF MORE THAN ONE METHOD CODE CIRCLED IN 304, CIRCLE CODE FOR HIGHEST METHOD IN LIST.	NO CODE CIRCLED . . . . . 00<br><br>MALE STERILIZATION . . . . . 02<br>IUD . . . . . 03<br>INJECTABLES . . . . . 04<br>IMPLANTS . . . . . 05<br>PILL . . . . . 06<br>CONDOM . . . . . 07<br>FEMALE CONDOM . . . . . 08<br>DIAPHRAGM . . . . . 09<br>FOAM/JELLY . . . . . 10<br>STANDARD DAYS METHOD . . . . . 11<br>LACTATIONAL AMEN. METHOD . . . . . 12<br>RHYTHM METHOD . . . . . 13<br>WITHDRAWAL . . . . . 14<br>OTHER MODERN METHOD . . . . . 95<br>OTHER TRADITIONAL METHOD 96	→ 324A<br><br>→ 324A<br><br><br><br><br>→ 323 (07)<br>→ 320 (08–10)<br><br><br>→ 324A (11–13)<br><br><br>→ 324A (14–96)
317	At that time, were you told about side effects or problems you might have with the method?	YES . . . . . 1<br>NO 2	→ 319
318	Were you ever told by a health or family planning worker about side effects or problems you might have with the method?	YES . . . . . 1<br>NO 2	 → 320
319	Were you told what to do if you experienced side effects or problems?	YES . . . . . 1<br>NO 2	
320	CHECK 317:<br><br>CODE '1' CIRCLED ☐ ↓<br>At that time, were you told about other methods of family planning that you could use?<br><br>CODE '1' NOT CIRCLED ☐ ↓<br>When you obtained (CURRENT METHOD FROM 314) from (SOURCE OF METHOD FROM 307 OR 315), were you told about other methods of family planning that you could use?	YES . . . . . 1<br>NO 2	→ 322

NO.	QUESTIONS AND FILTERS	CODING CATEGORIES	SKIP
321	Were you ever told by a health or family planning worker about other methods of family planning that you could use?	YES . . . . 1<br>NO 2	
322	CHECK 304:<br><br>CIRCLE METHOD CODE:<br><br>IF MORE THAN ONE METHOD CODE CIRCLED IN 304, CIRCLE CODE FOR HIGHEST METHOD IN LIST.	MALE STERILIZATION . . . . . 02<br>IUD . . . . 03<br>INJECTABLES . . . . 04<br>IMPLANTS . . . . 05<br>PILL . . . . 06<br>CONDOM . . . . 07<br>FEMALE CONDOM . . . . 08<br>DIAPHRAGM . . . . 09<br>FOAM/JELLY . . . . 10<br>STANDARD DAYS METHOD . . . . 11<br>LACTATIONAL AMEN. METHOD . . 12<br>RHYTHM METHOD . . . . 13<br>WITHDRAWAL . . . . 14<br>OTHER MODERN METHOD . . . . 95<br>OTHER TRADITIONAL METHOD . . 96	02 → 324A<br><br>11–96 → 324A
323	Where did you obtain (CURRENT METHOD) the last time? **(5)**<br><br>PROBE TO IDENTIFY THE TYPE OF SOURCE.	**PUBLIC SECTOR**<br>GOVT. HOSPITAL . . . . . . 11<br>GOVT. HEALTH CENTER . . . . 12<br>GOV'T HEALTH POST/<br>OUTREACH . . . . 13<br>MOBILE CLINIC . . . . . . 14<br>HSA . . . . 15<br>CBDA / DOOR-TO-DOOR . . . . 16<br>OTHER PUBLIC<br>SECTOR ________ 17<br>(SPECIFY)<br><br>**CHAM / MISSION**<br>HOSPITAL . . . . 21<br>HEALTH CENTER . . . . . . 22<br>MOBILE CLINIC . . . . . . 23<br>DOOR-TO-DOOR . . . . . . 24<br><br>**PRIVATE MEDICAL SECTOR**<br>PRIVATE HOSPITAL / CLINIC . . 31<br>PHARMACY . . . . 32<br>PRIVATE DOCTOR . . . . . . 33<br>MOBILE CLINIC . . . . . . 34<br>CBDA / DOOR-TO-DOOR . . . . 35<br>OTHER PRIVATE MEDICAL<br>SECTOR ________ 36<br>(SPECIFY)<br><br>**BANJA LA MTSOGOLO (BLM)** 41<br>**MACRO** 51<br>**TUNZA (PSI) CLINIC** 61<br>**YOUTH DROP IN CENTRE** 71<br><br>**OTHER SOURCE**<br>SHOP . . . . 81<br>CHURCH . . . . 82<br>FRIEND/RELATIVE . . . . . 83<br><br>OTHER ________ 96<br>(SPECIFY)	11–36 → 323A<br><br>41–96 → 323A

NO.	QUESTIONS AND FILTERS	CODING CATEGORIES	SKIP
323A	How many kilometers did you have to travel to reach this (SERVICE PROVIDER) to receive (CURRENT METHOD)?	KM . . . . [ ][ ]	
323B	How many minutes did it take for you to travel to this (SERVICE PROVIDER) to receive (CURRENT METHOD)?	MINUTES . . . . [ ][ ][ ][ ]	
323B_2	What mode(s) of transportation did you use to travel to this (SERVICE PROVIDER) to receive (CURRENT METHOD)?	NONE (RECEIVED AT HOME) 1<br>WALK 2<br>BICYCLE 3<br>MOTORCYCLE . . . . 4<br>BUS . . . . 5<br>CAR / TAXI 6<br>OTHER . . . . 96<br>DON'T KNOW 88<br>REFUSED 99	
323C	How much, in Malawian Kwacha, did you have to pay in transportation costs to go to this (SERVICE PROVIDER)?	MKW . [ ][ ][ ][ ][ ][ ]	
323D	How many minutes did you have to wait at the (SERVICE PROVIDER) before you received (CURRENT METHOD)?	MINUTES . . . . [ ][ ][ ][ ]	
324A	At any time during your current / last pregnancy, were you counselled about family planning / birth spacing?	YES . . . . 1<br>NO . . . . 2<br>DON'T KNOW . . . . 88<br>REFUSED 99	NO, DON'T KNOW, REFUSED → 325
324B	How many times did you receive information or counseling on family planning / birth spacing during your current / last pregnancy?	[ ][ ] TIMES COUNSELED<br>DON'T KNOW . . . . 88<br>REFUSED 99	
324C	Did you receive that counseling in a group with other women, individually with the provider, or both?	INDIVIDUAL COUNSELING ONLY 1<br>GROUP COUNSELING ONLY 2<br>INDIVIDUAL + GROUP COUNSELING 3<br>DON'T KNOW . . . . 88<br>REFUSED 99	

NO.	QUESTIONS AND FILTERS	CODING CATEGORIES	SKIP
324D	In which language(s) were you counseled?<br><br>SELECT ALL THAT APPLY.	CHICHEWA . . . . 1<br>TUMBUKA . . . . 2<br>ENGLISH . . . . 3<br>OTHER . . . . 96<br>DON'T REMEMBER/DON'T KNOW 88<br>REFUSED 99	
324E	Which method(s) were you counseled on?<br><br>CIRCLE ALL MENTIONED.	FEMALE STERILIZATIOI . . . . A<br>MALE STERILIZATION . . . . B<br>IUD . . . . C<br>INJECTABLES . . . . D<br>IMPLANTS . . . . E<br>PILL . . . . F<br>CONDOM . . . . G<br>FEMALE CONDOM . . . . H<br>DIAPHRAGM . . . . I<br>FOAM/JELLY . . . . J<br>STANDARD DAYS METHOD . . . . K<br>LACTATIONAL AMEN. METHOD . . . . . L<br>RHYTHM METHOD . . . . M<br>WITHDRAWAL . . . . N<br>OTHER MODERN METHOD . . . . X<br>OTHER TRADITIONAL METHOD . . . Y	
324F	Where did you receive counseling the last time?<br><br>PROBE TO IDENTIFY THE TYPE OF SOURCE.	**PUBLIC SECTOR**<br>GOVT. HOSPITAL . . . . 11<br>GOVT. HEALTH CENTER . . . . 12<br>GOV'T HEALTH POST/<br>OUTREACH . . . . 13<br>MOBILE CLINIC . . . . 14<br>HSA . . . . 15<br>CBDA / DOOR-TO-DOOR . . . . 16<br>OTHER PUBLIC<br>SECTOR ____________________ 17<br>(SPECIFY)<br><br>**CHAM / MISSION**<br>HOSPITAL . . . . 21<br>HEALTH CENTER . . . . 22<br>MOBILE CLINIC . . . . 23<br>DOOR-TO-DOOR . . . . 24<br><br>**PRIVATE MEDICAL SECTOR**<br>PRIVATE HOSPITAL / CLINIC . . . . . 31<br>PHARMACY . . . . 32<br>PRIVATE DOCTOR . . . . 33<br>MOBILE CLINIC . . . . 34<br>CBDA / DOOR-TO-DOOR . . . . 35<br>OTHER PRIVATE MEDICAL<br>SECTOR ____________________ 36<br>(SPECIFY)<br><br>**BANJA LA MTSOGOLO (BLM)** 41<br>**MACRO** 51<br>**TUNZA (PSI) CLINIC** 61<br>**YOUTH DROP IN CENTRE** 71<br><br>**OTHER SOURCE**<br>SHOP . . . . 81<br>CHURCH . . . . 82<br>FRIEND/RELATIVE . . . . 83<br><br>OTHER ____________________ 96<br>(SPECIFY)	11–36 → 325<br><br>41–96 → 325

NO.	QUESTIONS AND FILTERS	CODING CATEGORIES	SKIP
325	In choosing a contraceptive method, what feature(s) would be most important to you?<br><br>SELECT ALL THAT APPLY.	HOW EFFECTIVE IT IS AT PREVENTING PREGNANCY 1<br>CAN BE USED WITHOUT ANYONE ELSE KNOWING 2<br>THAT IT PROTECTS AGAINST STI/HIV . . . . 3<br>NO RISK OF HARMING HEALTH . . . . 4<br>NO EFFECT ON REGULAR MONTHLY BLEEDING 5<br>NO UNPLEASANT SIDE EFFECTS . . . . 6<br>EASY TO USE . . . . 7<br>EASY TO OBTAIN . . . . 8<br>CAN BE USED FOR A LONG TIME . . . . 9<br>WILL BE ABLE TO GET PREGNANT WHEN I WANT 10<br>OTHER______________ 96<br>(SPECIFY)<br>DON'T KNOW . . . . 88<br>REFUSED 99	
326	In the last 12 months, were you visited by a fieldworker who talked to you about family planning? **(8)**	YES . . . . 1<br>NO 2	
327	In the last 12 months, have you visited a health facility for care for yourself (or your children)?	YES . . . . 1<br>NO 2	 → 401
328	Did any staff member at the health facility speak to you about family planning methods?	YES . . . . 1<br>NO 2	

(1) If Standard Days Method is commonly used, it may be added to the table before Lactational Amenorrhea. "**Standard Days Method** (use local term, such as CycleBeads™ , as appropriate) PROBE: A woman uses a string of colored beads to know the days she can get pregnant. On the days she can get pregnant, she uses a condom or does not have sexual intercourse." If Standard Days Method is added to Q. 301, it should also be added before LAM to Qs. 304, 314, 316, 322, and Column 1 of the calendar.

(2) The LAM method should be deleted in countries that do not have a LAM program. In these countries, LAM should also be deleted as a coding category in Qs. 304, 314, 316, 322, and Column 1 of the calendar. A description of LAM should not be provided in Q. 301.

(3) Studies have indicated emergency contraception can be effective up to five days. Verify country program recommendations and modify wording if appropriate.

(4) Other commonly used methods may be added to the list, such as contraceptive patch, contraceptive vaginal ring, or sponge. Any codes added in Q. 304 must also be added to Qs. 314, 316, 322, and Column 1 of the calendar. These methods should not be added to Q. 301.

(5) Coding categories to be developed locally and revised based on the pretest; however, the broad categories must be maintained.

(6) Year of fieldwork is assumed to be 2010. For fieldwork beginning in 2011 or 2012, the year should be 2006 or 2007, respectively.

(7) Year of fieldwork is assumed to be 2010. For fieldwork beginning in 2011 or 2012, the year should be 2005 or 2006, respectively.

(8) In countries without national fieldworker programs that include family planning, Q. 326 should be deleted.

SECTION 4. PREGNANCY AND POSTNATAL CARE

401	CHECK 224:<br>ONE OR MORE BIRTHS IN 2016 **(1)** OR LATER ☐ ↓<br>NO BIRTHS IN 2016 **(1)** OR LATER ☐ →		601
402	CHECK 215: ENTER IN THE TABLE THE BIRTH HISTORY NUMBER, NAME, AND SURVIVAL STATUS OF THE LAST BIRTH IN 2015. ASK THE QUESTIONS ABOUT THIS BIRTH.<br><br>Now I would like to ask some questions about your last birth.		
403	BIRTH HISTORY NUMBER FROM 212 IN BIRTH HISTORY	LAST BIRTH<br>BIRTH HISTORY NUMBER ☐☐	
404	FROM 212 AND 216	NAME ________________<br>LIVING ☐ ↓ DEAD ☐ ↓	
405	When you got pregnant with (NAME), did you want to get pregnant at that time?	YES . . . . 1<br>(SKIP TO 430)<br>NO 2	
406	Did you want to have a baby later on, or did you not want any (more) children?	LATER . . . . 1<br>NO MORE . . . . 2<br>(SKIP TO 430)	
407	How much longer did you want to wait?	MONTHS . . 1 ☐☐<br>YEARS . . 2 ☐☐<br>DON'T KNOW . . . 998	
430	When (NAME) was born, was he/she very large, larger than average, average, smaller than average, or very small?	VERY LARGE . . . . . 1<br>LARGER THAN AVERAGE . . . . . 2<br>AVERAGE . . . . 3<br>SMALLER THAN AVERAGE . . . . . 4<br>VERY SMALL . . . . . 5<br>DON'T KNOW 8	
431	Was (NAME) weighed at birth?	YES . . . . 1<br>NO . . . . 2<br>(SKIP TO 447)<br>DON'T KNOW 8	
432	How much did (NAME) weigh?<br><br>RECORD WEIGHT IN KILOGRAMS FROM HEALTH CARD, IF AVAILABLE.	KG FROM CARD<br>1 ☐ . ☐☐☐<br><br>KG FROM RECALL<br>2 ☐ . ☐☐☐<br><br>DON'T KNOW 99998	

NO.	QUESTIONS AND FILTERS	LAST BIRTH<br>NAME ________________
447	Has your menstrual period returned since the birth of (NAME)?	YES . . . . 1<br>(SKIP TO 449)<br>NO . . . . 2<br>(SKIP TO 450)
448	Did your period return between the birth of (NAME) and your next pregnancy?	
449	For how many months after the birth of (NAME) did you not have a period?	MONTHS . . . [ ][ ]<br>DON'T KNOW 98
450	CHECK 226:<br>IS RESPONDENT PREGNANT?	NOT PREG-NANT [ ]<br>PREGNANT OR UNSURE [ ] (SKIP TO 452)
451	Have you had sexual intercourse since the birth of (NAME)?	YES . . . . 1<br>NO . . . . 2<br>(SKIP TO 453)
452	For how many months after the birth of (NAME) did you not have sexual intercourse?	MONTHS . . . [ ][ ]<br>DON'T KNOW 98
453	Did you ever breastfeed (NAME)?	YES . . . . 1<br>(SKIP TO 455)<br>NO 2
454	CHECK 404:<br>IS CHILD LIVING?	LIVING [ ] (SKIP TO 601)<br>DEAD [ ] (GO TO 601)

NO.	QUESTIONS AND FILTERS	LAST BIRTH<br>NAME ______________
455	How long after birth did you first put (NAME) to the breast?<br><br>IF LESS THAN 1 HOUR, RECORD '00' HOURS.<br>IF LESS THAN 24 HOURS, RECORD HOURS.<br>OTHERWISE, RECORD DAYS.	IMMEDIATELY . . . 000<br><br>HOURS 1 [ ][ ]<br>DAYS 2 [ ][ ]
456	In the first three days after delivery, was (NAME) given anything to drink other than breast milk?	YES . . . . 1<br>NO . . . . 2<br>(SKIP TO 458)
457	What was (NAME) given to drink?<br><br>Anything else?<br><br>RECORD ALL LIQUIDS MENTIONED.	MILK (OTHER THAN BREAST MILK) A<br>PLAIN WATER . . . B<br>SUGAR OR GLU-COSE WATER . . . C<br>GRIPE WATER . . . D<br>SUGAR-SALT-WATER SOLUTION . . . . . E<br>FRUIT JUICE . . . . . F<br>INFANT FORMULA G<br>TEA/INFUSIONS . . . H<br>COFFEE . . . . I<br>HONEY . . . . J<br><br>OTHER __________ X<br>(SPECIFY)
458	CHECK 404:<br><br>IS CHILD LIVING?	LIVING [ ] DEAD [ ]<br>(GO TO 601)
459	Are you still breastfeeding (NAME)?	YES . . . . 1<br>NO 2

(1) Year of fieldwork is assumed to be 2010. For fieldwork beginning in 2011 or 2012, the year should be 2006 or 2007, respectively.
(2) Coding categories to be developed locally and revised based on the pretest; however, the broad categories must be maintained.
(3) Vaccination practices may vary; this question should specify where the injection is given, e.g. arm or shoulder.
(4) Syrup should be deleted in countries where syrup is not used.
(5) In countries where it is important to know the number of iron tablets taken per day, an appropriate question may be added.
(6) The question should be deleted in surveys in countries where there is no program for intermittent preventive treatment against malaria during pregnancy.

	Now I would like to ask you some questions about your recent sexual activity. Let me assure you again that your answers are completely confidential and will not be told to anyone. If we should come to any question that you don't want to answer, just let me know and we will go to the next question.<br><br>CHECK FOR THE PRESENCE OF OTHERS. BEFORE CONTINUING, MAKE EVERY EFFORT TO ENSURE PRIVACY.

NO.	QUESTIONS AND FILTERS	CODING CATEGORIES	SKIP
601	Are you currently married or living together with a man as if married?	YES, CURRENTLY MARRIED . . . . 1<br>YES, LIVING WITH A MAN . . . . 2<br>NO, NOT IN UNION 3	1, 2 → 604
602	Have you ever been married or lived together with a man as if married?	YES, FORMERLY MARRIED . . . . 1<br>YES, LIVED WITH A MAN . . . . 2<br>NO 3	3 → 612
603	What is your marital status now: are you widowed, divorced, or separated?	WIDOWED . . . . 1<br>DIVORCED . . . . 2<br>SEPARATED 3	→ 609
604	Is your (husband/partner) living with you now or is he staying elsewhere?	LIVING WITH HER . . . . 1<br>STAYING ELSEWHERE 2	
605	RECORD THE HUSBAND'S/PARTNER'S NAME AND LINE NUMBER FROM THE HOUSEHOLD QUESTIONNAIRE.<br>IF HE IS NOT LISTED IN THE HOUSEHOLD, RECORD '00'.	NAME __________________________<br><br>LINE NO. . . . . [ ][ ]	
606<br>**(1)**	Does your (husband/partner) have other wives or does he live with other women as if married?	YES . . . . 1<br>NO . . . . 2<br>DON'T KNOW 8	2, 8 → 609
607<br>**(1)**	Including yourself, in total, how many wives or live-in partners does he have?	TOTAL NUMBER OF WIVES AND LIVE-IN PARTNERS . . . [ ][ ]<br><br>DON'T KNOW 98	
608<br>**(1)**	Are you the first, second, … wife?	RANK . . . . [ ][ ]	
609	Have you been married or lived with a man only once or more than once?	ONLY ONCE . . . . 1<br>MORE THAN ONCE 2	
610	CHECK 609:<br>MARRIED/ LIVED WITH A MAN ONLY ONCE ↓<br>In what month and year did you start living with your (husband/partner)?<br><br>MARRIED/ LIVED WITH A MAN MORE THAN ONCE ↓<br>Now I would like to ask about your first (husband/partner). In what month and year did you start living with him?	MONTH . . . . [ ][ ]<br><br>DON'T KNOW MONTH . . . . 98<br><br>YEAR . . . . [ ][ ][ ][ ]<br><br>DON'T KNOW YEAR 9998	YEAR → 612
611	How old were you when you first started living with him?	AGE . . . . [ ][ ]	

NO.	QUESTIONS AND FILTERS	CODING CATEGORIES	SKIP
612	CHECK FOR THE PRESENCE OF OTHERS. BEFORE CONTINUING, MAKE EVERY EFFORT TO ENSURE PRIVACY.		
613	Now I would like to ask some questions about sexual activity in order to gain a better understanding of some important life issues.<br><br>How old were you when you had sexual intercourse for the very first time?	NEVER HAD SEXUAL INTERCOURSE . . . . 00<br><br>AGE IN YEARS . . . . [ ][ ]<br><br>FIRST TIME WHEN STARTED LIVING WITH (FIRST) HUSBAND/PARTNER 95	→ 701
615	When was the <u>last</u> time you had sexual intercourse?<br><br>IF LESS THAN 12 MONTHS, ANSWER MUST BE RECORDED IN DAYS, WEEKS OR MONTHS.<br>IF 12 MONTHS (ONE YEAR) OR MORE, ANSWER MUST BE RECORDED IN YEARS.	DAYS AGO . . . . 1 [ ][ ]<br>WEEKS AGO . . . . 2 [ ][ ]<br>MONTHS AGO . . . . 3 [ ][ ]<br>YEARS AGO . . . . 4 [ ][ ]	

SECTION 7. FERTILITY PREFERENCES

NO.	QUESTIONS AND FILTERS	CODING CATEGORIES	SKIP
701	CHECK 304:<br>NEITHER STERILIZED ☐ ↓<br>HE STERILIZED ☐ →		712
702	CHECK 226:<br>PREGNANT ☐ ↓<br>NOT PREGNANT OR UNSURE ☐ →		704
703	Now I have some questions about the future. After the child you are expecting now, would you like to have another child, or would you prefer not to have any more children?	HAVE ANOTHER CHILD . . . . 1<br>NO MORE . . . . 2<br>UNDECIDED/DON'T KNOW 8	→ 705<br><br>→ 711 (2, 8)
704	Now I have some questions about the future. Would you like to have (a/another) child, or would you prefer not to have any (more) children?	HAVE (A/ANOTHER) CHILD . . . . 1<br>NO MORE/NONE . . . . 2<br>SAYS SHE CAN'T GET PREGNANT 3<br>UNDECIDED/DON'T KNOW 8	 → 707<br>→ 712<br>→ 710
705	CHECK 226:<br>NOT PREGNANT OR UNSURE ☐ ↓<br>How long would you like to wait from now before the birth of (a/another) child?<br>PREGNANT ☐ ↓<br>After the birth of the child you are expecting now, how long would you like to wait before the birth of another child?	MONTHS . . . . 1 ☐☐<br>YEARS . . . . 2 ☐☐<br>SOON/NOW . . . . 993<br>SAYS SHE CAN'T GET PREGNANT 994<br>AFTER MARRIAGE . . . . 995<br>OTHER ______________________ 996<br>(SPECIFY)<br>DON'T KNOW 998	 <br>→ 710<br>→ 712<br><br>→ 710 (995, 996, 998)
706	CHECK 226:<br>NOT PREGNANT OR UNSURE ☐ ↓<br>PREGNANT ☐ →		711
707	CHECK 303: USING A CONTRACEPTIVE METHOD?<br>NOT CURRENTLY USING ☐ ↓<br>CURRENTLY USING ☐ →		712
708	CHECK 705:<br>NOT ASKED ☐ ↓<br>24 OR MORE MONTHS OR 02 OR MORE YEARS ☐ ↓<br>00-23 MONTHS OR 00-01 YEAR ☐ →		711

NO.	QUESTIONS AND FILTERS	CODING CATEGORIES	SKIP
709	CHECK 704:<br><br>WANTS TO HAVE A/ANOTHER CHILD ☐ ↓ / WANTS NO MORE/ NONE ☐ ↓<br><br>(WANTS TO HAVE A/ANOTHER CHILD) You have said that you do not want (a/another) child soon.<br>Can you tell me why you are not using a method to prevent pregnancy?<br><br>Any other reasons?<br><br>(WANTS NO MORE/NONE) You have said that you do not want any (more) children.<br>Can you tell me why you are not using a method to prevent pregnancy?<br><br>Any other reasons?<br><br>RECORD ALL REASONS MENTIONED.	NOT MARRIED . . . . A<br><br>FERTILITY-RELATED REASONS<br>NOT HAVING SEX . . . . B<br>INFREQUENT SEX . . . . C<br>MENOPAUSAL/HYSTERECTOMY D<br>CAN'T GET PREGNANT . . . . E<br>NOT MENSTRUATED SINCE LAST BIRTH . . . . F<br>BREASTFEEDING . . . . G<br>UP TO GOD/FATALISTIC . . . . H<br><br>OPPOSITION TO USE<br>RESPONDENT OPPOSED . . . . I<br>HUSBAND/PARTNER OPPOSED . . . J<br>OTHERS OPPOSED . . . . K<br>RELIGIOUS PROHIBITION . . . . L<br><br>LACK OF KNOWLEDGE<br>KNOWS NO METHOD . . . . M<br>KNOWS NO SOURCE . . . . N<br><br>METHOD-RELATED REASONS<br>HEALTH CONCERNS . . . . O<br>FEAR OF SIDE EFFECTS . . . . P1<br>LACK OF ACCESS/TOO FAR . . . . . P2<br>TOO LONG OF A WAIT . . . . P3<br>TOO BUSY/NO TIME . . . . P4<br>COSTS TOO MUCH . . . . Q<br>PREFERRED METHOD NOT AVAILABLE . . . . R<br>NO METHOD AVAILABLE . . . . S<br>INCONVENIENT TO USE . . . . T<br>INTERFERES WITH BODY'S NORMAL PROCESSES . . . . U<br><br>OTHER ______________________ X<br>(SPECIFY)<br>DON'T KNOW . . . . Z	
710	CHECK 303: USING A CONTRACEPTIVE METHOD?<br><br>NOT ASKED ☐ ↓ / NO, NOT CURRENTLY USING ☐ ↓ / YES, CURRENTLY USING ☐ → 712		712
711	Do you think you will use a contraceptive method to delay or avoid pregnancy at any time in the future?	YES . . . . 1<br>NO . . . . 2<br>DON'T KNOW 8	
712	CHECK 216:<br><br>HAS LIVING CHILDREN ☐ ↓ / NO LIVING CHILDREN ☐ ↓<br><br>(HAS LIVING CHILDREN) If you could go back to the time you did not have any children and could choose exactly the number of children to have in your whole life, how many would that be?<br><br>(NO LIVING CHILDREN) If you could choose exactly the number of children to have in your whole life, how many would that be?<br><br>PROBE FOR A NUMERIC RESPONSE.	NONE . . . . 00<br><br>NUMBER . . . . ☐☐<br><br>OTHER ______________________ 96<br>(SPECIFY)	NONE → 716<br><br>OTHER → 716

NO.	QUESTIONS AND FILTERS	CODING CATEGORIES	SKIP
713	How many of these children would you like to be boys, how many would you like to be girls and for how many would it not matter if it's a boy or a girl?	BOYS / GIRLS / EITHER<br>NUMBER [ ][ ] / [ ][ ] / [ ][ ]<br>OTHER ____________ 96<br>(SPECIFY)	
714A	In the last few months have you heard about family planning:<br>On the radio?<br>On the television?<br>In a newspaper or magazine?<br>On a poster?<br>On clothing (i.e., cap, chitenji, t-shirt)?<br>In a drama?<br>Somewhere else?	YES NO<br>RADIO . . . . 1 2<br>TELEVISION . . . . 1 2<br>NEWSPAPER OR MAGAZINE . . . 1 2<br>POSTER . . . . 1 2<br>CLOTHING . . . . 1 2<br>DRAMA . . . . 1 2<br>OTHER 1 2	
714B	In the last few months, have you listened to any of the following program series about family planning or health on the radio?<br>Safe motherhood?<br>Phukusi la Moyo?<br>Radio Doctor/Doctor wapawairesi?<br>Umoyo M'Malawi?<br>Tikuferanji?<br>Chitukuku M'Malawi?<br>Uku ndiko kudya?<br>Other?	YES NO<br>SAFE MOTHERHOOD . . . . 1 2<br>PHUKUSI LA MOYO . . . . 1 2<br>RADIO DOCTOR . . . . 1 2<br>UMOYO M'MALAWI . . . . 1 2<br>TIKUFERANJI . . . . 1 2<br>CHITUKUKU M'MALAWI . . . . 1 2<br>UKU NDIKO KUDYA . . . . 1 2<br>OTHER 1 2	
716	CHECK 601:<br>YES, CURRENTLY MARRIED [ ]↓ YES, LIVING WITH A MAN [ ]↓ NO, NOT IN UNION [ ]		→ 801
717	CHECK 303: USING A CONTRACEPTIVE METHOD?<br>CURRENTLY USING [ ]↓ NOT CURRENTLY USING OR NOT ASKED [ ]		→ 720
718A	Does your husband/partner know that you are using a method of family planning?	YES . . . . 1<br>NO . . . . 2<br>DON'T KNOW 8	
718B	Would you say that using contraception is mainly your decision, mainly your (husband's/partner's) decision, or did you both decide together?	MAINLY RESPONDENT . . . . 1<br>MAINLY HUSBAND/PARTNER . . . . . 2<br>JOINT DECISION . . . . 3<br>OTHER ____________ 6<br>(SPECIFY)	
719	CHECK 304:<br>NEITHER STERILIZED [ ]↓ HE OR SHE STERILIZED [ ]		→ 801
720	Does your (husband/partner) want the same number of children that you want, or does he want more or fewer than you want?	SAME NUMBER . . . . 1<br>MORE CHILDREN . . . . 2<br>FEWER CHILDREN . . . . 3<br>DON'T KNOW 8	

NO.	QUESTIONS AND FILTERS	CODING CATEGORIES	SKIP
721	If you were to not use any family planning method, how likely do you think it is that you will become pregnant during the next year?	VERY UNLIKELY . . . . 1<br>SOMEWHAT UNLIKELY . . . . 2<br>NEUTRAL . . . . 3<br>SOMEWHAT LIKELY . . . . 4<br>VERY LIKELY 5	
722	CHECK 303: USING A CONTRACEPTIVE METHOD?<br>CURRENTLY USING ☐ ↓	NOT CURRENTLY USING OR NOT ASKED ☐ →		725
723	If you were to continue to use your family planning method, how likely do you think it is that you would become pregnant during the next year?	VERY UNLIKELY . . . . 1<br>SOMEWHAT UNLIKELY . . . . 2<br>NEUTRAL . . . . 3<br>SOMEWHAT LIKELY . . . . 4<br>VERY LIKELY 5	
724A	Different methods of family planning vary in how effective or ineffective they are in preventing pregnancy. How effective do you think that your family planning method is in preventing pregnancy?	VERY EFFECTIVE . . . . 1<br>PRETTY EFFECTIVE . . . . 2<br>UNSURE/NEUTRAL . . . . 3<br>PRETTY INEFFECTIVE . . . . 4<br>VERY INEFFECTIVE 5	
724B	Different methods of family planning vary in how convenient they are to use. How convenient is it to use your family planning method?	VERY CONVENIENT . . . . 1<br>PRETTY CONVENIENT . . . . 2<br>UNSURE/NEUTRAL . . . . 3<br>PRETTY INCONVENIENT . . . . 4<br>VERY INCONVENIENT 5	
725	If you were to get pregnant within the next year, would it be:<br><br>READ OUT OPTIONS AND CHOOSE ONE RESPONSE.	THE WORST THING THAT COULD HAPPEN TO YOU . . . . 1<br>VERY BAD . . . . 2<br>SORT OF BAD, BUT NOT TERRIBLE . . . . 3<br>OKAY . . . . 4<br>SORT OF GOOD, BUT NOT TERRIFIC . . . . 5<br>VERY GOOD . . . . 6<br>THE BEST THING THAT COULD HAPPEN TO YOU 7	

(1) These questions have been added by the researchers to identify sources of non-use and intention to use.

SECTION 8. HUSBAND'S BACKGROUND

NO.	QUESTIONS AND FILTERS	CODING CATEGORIES	SKIP
802	How old was your (husband/partner) on his last birthday?	AGE IN COMPLETED YEARS [ ][ ]	
803	Did your (last) (husband/partner) ever attend school?	YES . . . . 1<br>NO 2	 → 1008
804	What was the highest level of school he attended: primary, secondary, or higher? **(1)**	PRIMARY . . . . 1<br>SECONDARY . . . . 2<br>HIGHER . . . . 3<br>DON'T KNOW 8	 <br><br>→ 1008
805	What was the highest (class/form/year) he completed at that level? **(1)**<br>IF COMPLETED LESS THAN ONE YEAR AT THAT LEVEL, RECORD '00'.	CLASS . . . . [ ][ ]<br>DON'T KNOW 98	

(1) Revise according to the local educational system.

NO.	QUESTIONS AND FILTERS	CODING CATEGORIES			SKIP
1008	Many different factors can prevent women from getting family planning advice or treatment for themselves. When you want to get family planning advice or treatment, is each of the following a big problem or not?		BIG PROB-LEM	NOT A BIG PROB-LEM	
	Getting permission to go to the doctor?	PERMISSION TO GO . . .	1	2	
	Getting money needed for advice or treatment?	GETTING MONEY	1	2	
	The distance to the health facility?	DISTANCE	1	2	
	Not wanting to go alone?	GO ALONE	1	2	
	Not having time because of work?	NO TIME DUE TO WORK .	1	2	
1009	Are you covered by any health insurance? **(2)**	YES . . . . 1<br>NO 2			 → 1011
1010	What type of health insurance are you covered by? **(2)**<br><br>RECORD ALL MENTIONED.	MUTUAL HEALTH ORGANIZATION/ COMMUNITY-BASED HEALTH INSURANCE . . . . A<br>HEALTH INSURANCE THROUGH EMPLOYER . . . . B<br>SOCIAL SECURITY . . . . C<br>OTHER PRIVATELY PURCHASED COMMERCIAL HEALTH INSURANCE D<br>OTHER ______________________ X<br>(SPECIFY)			
1011	RECORD THE TIME.	HOUR . . . . [ ][ ]<br>MINUTES . . . . [ ][ ]			

(1) Add local terms.

(2) If a health service prepayment plan or other types of plans are available in the country, add those types of plans to the question.

INSTRUCTIONS:
ONLY ONE CODE SHOULD APPEAR IN ANY BOX.
COLUMN 1 REQUIRES A CODE IN EVERY MONTH.

INFORMATION TO BE CODED FOR EACH COLUMN

COLUMN 1: BIRTHS, PREGNANCIES, CONTRACEPTIVE USE**

B BIRTHS
P PREGNANCIES
T TERMINATIONS

0 NO METHOD
1 FEMALE STERILIZATION
2 MALE STERILIZATION
3 IUD
4 INJECTABLES
5 IMPLANTS
6 PILL
7 CONDOM
8 FEMALE CONDOM
9 DIAPHRAGM
J FOAM OR JELLY
K STANDARD DAYS METHOD/CYCLEBEADS
L LACTATIONAL AMENORRHEA METHOD
M RHYTHM METHOD
N WITHDRAWAL
X OTHER MODERN METHOD
Y OTHER TRADITIONAL METHOD

COLUMN 2: DISCONTINUATION OF CONTRACEPTIVE USE

0 INFREQUENT SEX/HUSBAND AWAY
1 BECAME PREGNANT WHILE USING
2 WANTED TO BECOME PREGNANT
3 HUSBAND/PARTNER DISAPPROVED
4 WANTED MORE EFFECTIVE METHOD
5 SIDE EFFECTS/HEALTH CONCERNS
6 LACK OF ACCESS/TOO FAR
7 COSTS TOO MUCH
8 INCONVENIENT TO USE
F UP TO GOD/FATALISTIC
A DIFFICULT TO GET PREGNANT/MENOPAUSAL
D MARITAL DISSOLUTION/SEPARATION
X OTHER ______________________
(SPECIFY)
Z DON'T KNOW

* Year of fieldwork is assumed to be 2010. For fieldwork beginning in 2011 or 2012, the years should be adjusted.

** Response categories may be added for other methods, including fertility awareness methods.

				1	2	
	12	DEC	01			
	11	NOV	02			
	10	OCT	03			
	09	SEP	04			
2	08	AUG	05			2
0	07	JUL	06			0
1	06	JUN	07			1
6	05	MAY	08			6
*	04	APR	09			*
	03	MAR	10			
	02	FEB	11			
	01	JAN	12			

SECTIONS 12 - 13. LABOR AND TIME USE

INTRODUCTION

As you know, some women take up jobs for which they are paid in cash or kind. Others sell things, have a small business or work on the family farm or in the family business.

In this section, I will ask you questions about work and time use. The questions in this section usually take about 30 to 45 minutes. All of the answers you give will be confidential and will not be shared with anyone other than members of our survey team. You don't have to be in the survey, but we hope you will agree to answer the questions since your views are important. If I ask you any question you don't want to answer, just let me know and I will go on to the next question or you can stop the interview at any time.

In case you need more information about the survey, you may contact the person listed on the card that has already been given to your household.
Do you have any questions? May I begin the interview now?

SECTION 12. LABOR FORCE STATUS (FOR THE WOMAN AND HER PARTNER/SPOUSE ONLY)

Ask the following questions to the woman about herself and her partner/spouse only

CHECK HH SCHEDULE FOR LINE NUMBERS.<br><br>LINE NO.	INTERVIEWER:<br><br>IS THE RESPONDENT ANSWERING FOR HERSELF?<br><br>1 = Yes<br>→ 1204<br><br>2 = No	WHAT IS THE ID CODE OF THE PERSON RESPONDING FOR [NAME]?	In the last week, did [NAME] work for a wage, salary, commission or any payment in kind; including doing paid domestic work, even if it was for only for one hour?<br><br>1 = Yes<br><br>2 = No<br>→ 1206	Did [NAME] do this type of work in the last 12 months?<br><br>1 = Yes<br><br>2 = No	In the last week, did [NAME] run a business of any size, for themselves or another household member, even if it was for only one hour?<br><br>1 = Yes<br><br>2 = No<br>→ 1208	Did [NAME] run a business in the last 12 months?<br><br>1 = Yes<br><br>2 = No	In the last week, did [NAME] help without being paid in any kind of business run by this household, even if it was only for one hour?<br><br>1 = Yes<br><br>2 = No<br>→ 1210	Did [NAME] do this in the last 12 months?<br><br>1 = Yes<br><br>2 = No	In the last week, was [NAME] an apprentice?<br><br>INCLUDE APPRENTICESHIPS THAT ARE PAID CASH, PAID IN KIND, UNPAID, OR FOR WHICH THE APPRENTICE PAYS TO PARTICIPATE<br><br>1 = Yes<br><br>2 = No<br>→ 1212	Was [NAME] an apprentice in the last 12 months?<br><br>1 = Yes<br><br>2 = No	CHECK HH QUESTION 119. IF 'NO', SKIP TO 1214<br><br>In the last week, did [NAME] work on this household's farm?<br><br>EXAMPLE: TENDING CROPS, FEEDING [illegible]<br><br>1 = Yes<br><br>2 = No<br>→ 1214	Did [NAME] work on the household's farm in the past 12 months?<br><br>1 = Yes<br><br>2 = No	AMONG THE ANSWERS TO 1204, 1206, 1208, 1210 AND 1212, IS THERE A "YES" (CODE 1)?<br><br>1 = Yes<br>→ 1219<br><br>2 = No	Even if [NAME] did not do any work for pay or profit, did not help without pay in household business and did not participate in an apprenticeship in the last 7days, did [NAME] have a job or business they will definitely return to?<br><br>1 = Yes<br>→ 1219<br><br>2 = No
**1201**	**1202**	**1203**	**1204**	**1205**	**1206**	**1207**	**1208**	**1209**	**1210**	**1211**	**1212**	**1213**	**1214**	**1215**

SECTION 12. LABOR FORCE STATUS CONT'D (FOR THE WOMAN AND HER PARTNER/SPOUSE ONLY)

Ask the following questions to the woman about herself and her partner/spouse only

				MAIN JOB				
LINE NO.	In the last four weeks, was [NAME] looking for any kind of job?<br><br>1 = Yes<br>2 = No	In the last four weeks, was [NAME] trying to start any kind of business?<br><br>1 = Yes<br>→ 1259<br>2 = No	What best describes [NAME]'s situation at this time? For example, [NAME] is ill, disabled, in school, taking care of household family, or something else?<br><br>2 = Disabled<br>3 = In school<br>4 = Taking care of house or family<br>5 = Retired<br>6 = Waiting for reply from employer<br>7 = Waiting for busy season<br>8 = Other (specify)<br><br>[→ 1259]	What kind of work does [NAME] usually do in the (main) job/business that [NAME] had during the last week? FOR THE CODE FOR QUESTION 1219, USE ANNEX 1.<br><br>CODE	What are the main goods/services produced at [NAME]'s place of work or its main function?<br><br>FOR THE CODE FOR QUESTION 1220, USE ANNEX 2.<br><br>CODE	When did [NAME] start to work for this employer or start running the business?<br><br>YEAR	 <br>MONTH	In this (main) job/business that [NAME] had during the last week, was [NAME]<br><br>1 = Working for someone else for pay?<br>2 = An employer? → 1232<br>3 = An own-account worker? → 1232<br>4 = Helping without pay in a household business? → 1232<br>5=An apprentice? → 1234<br>6 = Working on the household farm or with household livestock?
**1201**	**1216**	**1217**	**1218**	**1219**	**1220**	**1221A**	**1221B**	**1222**

SECTION 12. LABOR FORCE STATUS CONT'D (FOR THE WOMAN AND HER PARTNER/SPOUSE ONLY)

Ask the following questions to the woman about herself and her partner/spouse only

	MAIN JOB														
	FOR EMPLOYEES											FOR EMPLOYERS, OWN ACCOUNT WORKERS, AND UNPAID FAMILY WORKERS		FOR APPRENTICES	
	Does this employer contribute to any pension/ retirement fund for [NAME]?	Is [NAME] entitled to any paid leave from this employer?	Is [NAME] entitled to medical benefits from this employer?	Does this employer deduct or pay income tax (PAYE) from [NAME]'s salary/ wage?	Is [NAME]'s employment agreement	Is [NAME]'s position…	What is the duration of [NAME]'s employment agreement?	During the last 12 months, for how many months did [NAME] work in this job**?**	How much was [NAME]'s last cash payment and the estimated value of what [NAME] last received in kind for the main job during the last week?<br>What period of time did this payment cover?<br>CASH PAYMENTS SHOULD INCLUDE SET RATE, COMMISSIONS, TIPS AND CASH ALLOWANCES. IF NOT CASH OR IN-KIND PAYMENT WAS RECEIVED, RECORD '0' IN COL 1231A & 1231B.			Is [NAME]'s business (or household business where [NAME] works) registered for VAT?	Is [NAME]'s business (or household business where [NAME] works) registered for income tax?	In this apprenticeship was [NAME]?<br>READ TO RESPONDENT AND MARK UP TO 2.	
LINE NO.	1 = Yes<br>2 = No	1 = Yes<br>2 = No	1 = Yes<br>2 = No	1 = Yes<br>2 = No	1 = Written<br>2 = Verbal	1 = Permanent and pensionable → 1230<br>2 = An open-ended appointment → 1230<br>3 = A fixed term	1 = A week or less<br>2 = More than a week but less than a month<br>3 = One to six months<br>4 = Seven to eleven months<br>5 = One to five years<br>6 = More than 5 years	MONTHS	Cash	Estimated cash value of in-kind payments	Time<br>1 = Hour<br>2 = Day<br>3 = Week<br>4 = Month<br>5 = Other (specify)	1 = Yes<br>2 = No<br>8 = Don't know<br>9 = Refused	1 = Yes<br>2 = No<br>8 = Don't know<br>9 = Refused	A = Unpaid<br>B = Paid cash<br>C = Paid in kind<br>D = Required to pay to participate	
**1201**	**1223**	**1224**	**1225**	**1226**	**1227**	**1228**	**1229**	**1230**	**1231A**	**1231B**	**1231C**	**1232**	**1233**	**1234A**	**1234B**

SECTION 12. LABOR FORCE STATUS CONT'D (FOR THE WOMAN AND HER PARTNER/SPOUSE ONLY)

Ask the following questions to the woman about herself and her partner/spouse only

	MAIN JOB								In the last week, did [NAME] have more than one economic activity, such as a job, business, household enterprise or farm?	SECOND JOB				
	Is [NAME]'s employer /business (at [NAME]'s main job)<br><br>1 = National Government<br>2 = Local government<br>3 = Government controlled business<br>4 = A commercial bank<br>5 = A private enterprise (other than a commercial bank)<br>6 = Non-profit organization (NGO/CBO)<br>7 = A private household	During the last 7 days, how many hours did [NAME] work on each day?<br><br>ACTUAL NUMBER OF HOURS WORKED STARTING FROM THE PREVIOUS DAY AND GOING BACKWARDS ON MAIN JOB.							1 = Yes<br>2 = No<br>→ 1246	What kind of work do [NAME] usually do in the secondary job/business that [NAME] had during the last week?<br><br>FOR THE CODE FOR QUESTION 1238, USE ANNEX 1.	What are the main goods/services produced at [NAME]'s second place of work or its main function?<br><br>FOR THE CODE FOR QUESTION 1239, USE ANNEX 2.	When did [NAME] start to work for this employer or start running the business?		In this (second) job/business that [NAME] had during the last week, was [NAME]<br><br>1 = Working for someone else for pay?<br>2 = An employer?<br>3 = An own-account worker?<br>4 = Helping without pay in a household business?<br>5 = An apprentice?<br>6 = Working on the household farm or with household livestock?<br>→ 1243
LINE NO.		SUN	MON	TUES	WED	THU	FRI	SAT		CODE	CODE	YEAR	MONTH	
**1201**	**1235**	**1236A**	**1236B**	**1236C**	**1236D**	**1236E**	**1236F**	**1236G**	**1237**	**1238**	**1239**	**1240A**	**1240B**	**1241**

SECTION 12. LABOR FORCE STATUS CONT'D (FOR THE WOMAN AND HER PARTNER/SPOUSE ONLY)

Ask the following questions to the woman about herself and her partner/spouse only

	SECOND JOB CONT'D						Last week, would [NAME] have liked to work more hours than [NAME] actually worked, provided the extra hours had been paid?
	Is [NAME]'s employer /business (at second job)	Last week, how many hours did [NAME] actually work at the second job?	During the last 12 months, for how many months did [NAME] work in this job?	How much was [NAME]'s last cash payment and the estimated value of what [NAME] last received in kind for the main job during the last week?<br><br>What period of time did this payment cover?<br><br>CASH PAYMENTS SHOULD INCLUDE SET RATE, COMMISSIONS, TIPS AND CASH ALLOWANCES. IF NOT CASH OR IN-KIND PAYMENT WAS RECEIVED, RECORD '0' IN COL 1245A & 1245B.			
LINE NO.	1 = National Government<br>2 = Local government<br>3 = Government controlled business<br>4 = A commercial bank<br>5 = A private enterprise (other than a commercial bank)<br>6 = Non-profit organization (NGO/CBO)<br>7 = A private household	HOURS	MONTHS	Cash	Estimated cash value of in-kind payments	Time<br>1 = Hour<br>2 = Day<br>3 = Week<br>4 = Month<br>5 = Other (specify)	1 = Yes, in the current job<br>2 = Yes, in taking an additional job<br>3 = Yes, in a different job with more hours<br>4 = No<br>9 = Don't know
**1201**	**1242**	**1243**	**1244**	**1245A**	**1245B**	**1245C**	**1246**

SECTION 12. LABOR FORCE STATUS CONT'D (FOR THE WOMAN AND HER PARTNER/SPOUSE ONLY)

Ask the following questions to the woman about herself and her partner/spouse only

	NON-MARKET LABOR ACTIVITIES								
	In the last 7 days, how much time in hours did [NAME] spend collecting firewood for the household, including travel time?	In the last 7 days, how much time in hours did [NAME] spend fetching water for the household, including travel time?	In the last 7 days, how much time in hours did [NAME] spend constructing your dwelling, farm buildings, private roads, or wells?	In the last 7 days, how much time in hours did [NAME] spend making major repairs to their dwelling, farm buildings, private roads, or wells?	In the last 7 days, how much time in hours did [NAME] spend on milling and other food processing for the household?<br><br>(This includes threshing and milling grain, making butter and cheese, slaughtering livestock, curing hides and skins, preserving food for later consumption, making beer and alcohol, and other similar activities. It does not include preparing food for immediate consumption)	In the last 7 days, how much time in hours did [NAME] spend making handicrafts for household use?<br><br>(This includes making furniture, clothing, clay pots, baskets, mats, and other similar activities.)	In the last 7 days, how much time in hours did [NAME] spend on agriculture?<br><br>(This includes growing or gathering field crops, fruits, and vegetables; producing eggs and milk; burning charcoal; and other similar activities)	In the last 7 days, how much time in hours did [NAME] spend on hunting and fishing?<br><br>(This includes hunting animals and birds; catching fish, crabs, and shellfish; and other similar activities.)	Does [NAME] get income or support from any of the following sources?<br><br>LIST ALL THAT APPLY<br><br>A = Remittances<br>B = Charity/church<br>C = Retirement pension<br>D = NSSF<br>E = Welfare grants<br>F = Bursary/study loan<br>G = Other (specify)<br>H = None
LINE NO.	HOURS	HOURS	HOURS	HOURS	HOURS	HOURS	HOURS	HOURS	
**1201**	**1259**	**1260**	**1261**	**1262**	**1263**	**1264**	**1265**	**1266**	**1267**

SECTION 13. TIME USE IN THE LAST 24 HOURS

ASK THE FOLLOWING QUESTIONS TO THE WOMAN ABOUT HERSELF AND HER DAY. FOR EACH PERIOD, RECORD UP TO TWO ACTIVITIES.
Now, I would like to ask you about your day yesterday and how you spent your time.

Time Period	Activity 1 Code		
04h00 - 05h00			
05h00 - 06h00			
06h00 - 07h00			
07h00 - 08h00			
08h00 - 09h00			
09h00 - 10h00			
10h00 - 11h00			
11h00 - 12h00			
12h00 - 13h00			
13h00 - 14h00			
14h00 - 15h00			
15h00 - 16h00			
16h00 - 17h00			
17h00 - 18h00			
18h00 - 19h00			
19h00 - 20h00			
20h00 - 21h00			
21h00 - 22h00			
22h00 - 23h00			
23h00 - 0h00			
0h00 - 1h00			
1h00 - 2h00			
2h00 - 3h00			
3h00 - 4h00			

ACTIVITY CODES
Refer to Time Use Coding Book in the Appendix.

QUESTION NO.		CODES AND FILTERS	RESPONSE
**1301**	RECORD THE DAY OF THE WEEK FOR THE ACTIVITIES RECORDED ABOVE (THIS IS THE DAY BEFORE TODAY).	1 = Monday<br>2 = Tuesday<br>3 = Wednesday<br>4 = Thursday<br>5 = Friday<br>6 = Saturday<br>7 = Sunday	
**1302**	Was yesterday a typical day for you?	1 = Yes<br>2 = No, because I was ill.<br>3 = No, because it was a school/college/university holiday.<br>4 = No, because I was on leave from work.<br>5 = No, because there was a funeral, wedding, bereavement, etc.<br>6 = No, because I was looking after another family/household member.<br>7 = No, because there was some other family problem.<br>8 = No, because it was a weekend day (Saturday or Sunday).<br>9 = No, other reason.	
**1303**	Overall, how do you feel about the day that you just described?	1 = I was too busy/I had too much to do.<br>2 = I had a comfortable amount of things to do in the day.<br>3 = I was not busy enough/I did not have enough to do.	
**1304**	CHECK ACTIVITY CODES IN TIME USE SURVEY.<br><br>DOES THE WOMAN REPORT ACTIVITY CODES '1' OR '2' IN ANY OF THE TIME SLOTS ABOVE?	1 = Yes<br><br>2 = No → END	
**1305**	CHECK QUESTIONS 216 - 218.<br><br>DOES THE WOMAN REPORT HAVING AT LEAST ONE CHILD WHO IS 1) ALIVE (Q216 = '1'); 2) TWO YEARS OLD OR YOUNGER; AND 3) LIVING WITH THE WOMAN (Q218 = '1')?	1 = Yes<br><br>2 = No → END	
**1306**	Who took care of your youngest child(ren) during the day while you worked?	1 = Respondent → END<br>2 = Child's father → END<br>3 = Non-relative (daycare center, school, etc.) → END<br><br>4 = Older sibling<br>5 = Other relative (uncle/aunt, grandparent, etc.)<br>6 = Domestic worker/nanny<br>7 = Other	
**1307**	About how old is the person who cared for this child during this time?	1 = Under 12 years old<br>2 = 12 to 16 years old<br>3 = 17 to 64 years old<br>4 = 65 years or older	

WEIGHT, HEIGHT, AND HEMOGLOBIN MEASUREMENT FOR ELIGIBLE WOMEN AGE 18-35

214	CHECK COLUMN 9 IN HOUSEHOLD SCHEDULE. RECORD THE LINE NUMBER AND NAME FOR ELIGIBLE WOMAN RESPONDENT IN 215.		
		ELIGIBLE WOMAN	
215	LINE NUMBER FROM COLUMN 9<br>NAME FROM COLUMN 2	LINE NUMBER . . . . ☐☐<br>NAME ____________________	
216	WEIGHT IN KILOGRAMS **(10)**	KG. ☐☐☐.☐☐<br>NOT PRESENT . . . . 99994<br>REFUSED . . . . 99995<br>OTHER 99996	
217	HEIGHT IN CENTIMETERS	CM. ☐☐☐.☐<br>NOT PRESENT . . . . 9994<br>REFUSED . . . . 9995<br>OTHER 9996	
223	ASK CONSENT FOR ANEMIA TEST FROM RESPONDENT.	As part of this survey, we are asking people all over the country to take an anemia test. Anemia is a serious health problem that usually results from poor nutrition, infection, or chronic disease. This survey will assist the government to develop programs to prevent and treat anemia.<br><br>For the anemia testing, we will need a few drops of blood from a finger. The equipment used to take the blood is clean and completely safe. It has never been used before and will be thrown away after each test. The blood will be tested for anemia immediately, and the result will be told to you right away. The result will be kept strictly confidential and will not be shared with anyone other than members of our survey team.<br><br>Do you have any questions?<br>You can say yes to the test, or you can say no. It is up to you to decide.<br>Will you take the anemia test?	
224	CIRCLE THE APPROPRIATE CODE AND SIGN YOUR NAME.	GRANTED . . . . 1<br>RESPONDENT REFUSED . . . . . 2<br>____________________<br>(SIGN)<br><br>(IF REFUSED, GO TO END)	
225	PREGNANCY STATUS: CHECK 226 IN WOMAN'S QUESTIONNAIRE OR ASK:<br>Are you pregnant?	YES . . . . 1<br>NO . . . . 2<br>DK 8	
239	PREPARE EQUIPMENT AND SUPPLIES ONLY FOR THE ANEMIA TEST FOR WHICH CONSENT HAS BEEN OBTAINED AND PROCEED WITH THE TEST.		
240	RECORD HEMO-GLOBIN LEVEL HERE AND IN ANEMIA PAMPHLET **(11).**	G/DL . . . . ☐☐.☐<br>NOT PRESENT . . . . 994<br>REFUSED . . . . 995<br>OTHER 996	

WEIGHT, HEIGHT AND HEMOGLOBIN MEASUREMENT FOR CHILDREN AGE 0-5

201	CHECK COLUMN 11 IN HOUSEHOLD SCHEDULE. RECORD THE LINE NUMBER AND NAME FOR ALL ELIGIBLE CHILDREN 0-5 YEA[illegible] IN QUESTION 202. IF MORE THAN SIX CHILDREN, USE ADDITIONAL QUESTIONNAIRE(S).			
		CHILD 1	CHILD 2	CHILD 3
202	LINE NUMBER FROM COLUMN 11<br><br>NAME FROM COLUMN 2	LINE NUMBER . . . . . ☐☐<br><br>NAME ________	LINE NUMBER . . . . . ☐☐<br><br>NAME ________	LINE NUMBER . . . . . ☐☐<br><br>NAME ________
203	IF MOTHER INTERVIEWED, COPY MONTH AND YEAR OF BIRTH FROM BIRTH HISTORY AND ASK DAY; IF MOTHER NOT INTERVIEWED, ASK:<br>What is (NAME)'s birth date?	DAY . . . . ☐☐<br>MONTH . . . . . ☐☐<br>YEAR ☐☐☐☐	DAY . . . . ☐☐<br>MONTH . . . . . ☐☐<br>YEAR ☐☐☐☐	DAY . . . . ☐☐<br>MONTH . . . . . ☐☐<br>YEAR ☐☐☐☐
204	CHECK 203:<br>CHILD BORN IN JANUARY 2016 **(9)** OR LATER?	YES . . . . 1<br>NO . . . . 2<br>(GO TO 203 FOR NEXT CHILD OR, IF NO MORE CHILDREN, GO TO 214)	YES . . . . 1<br>NO . . . . 2<br>(GO TO 203 FOR NEXT CHILD OR, IF NO MORE CHILDREN, GO TO 214)	YES . . . . 1<br>NO . . . . 2<br>(GO TO 203 FOR NEXT CHILD OR, IF NO MORE CHILDREN, GO TO 214)
205	WEIGHT IN KILOGRAMS<br>**(10)**	KG. ☐☐.☐☐<br><br>NOT PRESENT . . . 9994<br>REFUSED . . . . 9995<br>OTHER 9996	KG. ☐☐.☐☐<br><br>NOT PRESENT . . . 9994<br>REFUSED . . . . 9995<br>OTHER 9996	KG. ☐☐.☐☐<br><br>NOT PRESENT . . . 9994<br>REFUSED . . . . 9995<br>OTHER 9996
206	HEIGHT IN CENTIMETERS	CM. ☐☐☐.☐<br><br>NOT PRESENT . . . 9994<br>REFUSED . . . . 9995<br>OTHER 9996	CM. ☐☐☐.☐<br><br>NOT PRESENT . . . 9994<br>REFUSED . . . . 9995<br>OTHER 9996	CM. ☐☐☐.☐<br><br>NOT PRESENT . . . 9994<br>REFUSED . . . . 9995<br>OTHER 9996
207	MEASURED LYING DOWN OR STANDING UP?	LYING DOWN . . . . 1<br>STANDING UF . . . . 2<br>NOT MEASUREL 3	LYING DOWN . . . . 1<br>STANDING UF . . . . 2<br>NOT MEASUREL 3	LYING DOWN . . . . 1<br>STANDING UF . . . . 2<br>NOT MEASUREL 3
209	LINE NUMBER OF PARENT/ OTHER ADULT RESPONSIBLE FOR THE CHILD (FROM COLUMN 1 OF HOUSEHOLD SCHEDULE). RECORD '00' IF NOT LISTED.	LINE NUMBER . . . . . ☐☐	LINE NUMBER . . . . . ☐☐	LINE NUMBER . . . . . ☐☐

		CHILD 1	CHILD 2	CHILD 3
210	ASK CONSENT FOR ANEMIA TEST FROM PARENT/OTHER ADULT IDENTIFIED IN 209 AS RESPONSIBLE FOR CHILD.	As part of this survey, we are asking people all over the country to take an anemia test. Anemia is a serious health problem that usually results from poor nutrition, infection, or chronic disease. This survey will assist the government to develop programs to prevent and treat anemia.<br><br>We ask that all children born in 2016 **(9)** or later take part in anemia testing in this survey and give a few drops of blood from a finger or heel. The equipment used to take the blood is clean and completely safe. It has never been used before and will be thrown away after each test.<br><br>The blood will be tested for anemia immediately, and the result will be told to you right away. The result will be kept strictly confidential and will not be shared with anyone other than members of our survey team.<br>Do you have any questions?<br>You can say yes to the test, or you can say no. It is up to you to decide.<br>Will you allow (NAME OF CHILD) to participate in the anemia test?		
211	CIRCLE THE APPROPRIATE CODE AND SIGN YOUR NAME.	GRANTED . . . . 1<br>(SIGN)<br>REFUSED 2	GRANTED . . . . 1<br>(SIGN)<br>REFUSED 2	GRANTED . . . . 1<br>(SIGN)<br>REFUSED 2
212	RECORD HEMOGLOBIN LEVEL HERE AND IN THE ANEMIA PAMPHLET **(11)**.	G/DL ☐☐.☐<br><br>NOT PRESENT . . . . .994<br>REFUSED . . . . .995<br>OTHER 996	G/DL ☐☐.☐<br><br>NOT PRESENT . . . . .994<br>REFUSED . . . . .995<br>OTHER 996	G/DL ☐☐.☐<br><br>NOT PRESENT . . . . .994<br>REFUSED . . . . .995<br>OTHER 996
213	GO BACK TO 203 IN NEXT COLUMN OF THIS QUESTIONNAIRE OR IN THE FIRST COLUMN OF THE NEXT PAGE; IF NO MORE CHILDREN, GO TO 214.			

		CHILD 4	CHILD 5	CHILD 6
202	LINE NUMBER FROM COLUMN 11<br><br>NAME FROM COLUMN 2	LINE NUMBER . . . . . ☐☐<br><br>NAME ________	LINE NUMBER . . . . . ☐☐<br><br>NAME ________	LINE NUMBER . . . . . ☐☐<br><br>NAME ________
203	IF MOTHER INTERVIEWED, COPY MONTH AND YEAR OF BIRTH FROM BIRTH HISTORY AND ASK DAY; IF MOTHER NOT INTERVIEWED, ASK:<br>What is (NAME)'s birth date?	DAY . . . . ☐☐<br>MONTH . . . . . ☐☐<br>YEAR ☐☐☐☐	DAY . . . . ☐☐<br>MONTH . . . . . ☐☐<br>YEAR ☐☐☐☐	DAY . . . . ☐☐<br>MONTH . . . . . ☐☐<br>YEAR ☐☐☐☐
204	CHECK 203:<br>CHILD BORN IN JANUARY 2016 **(9)** OR LATER?	YES . . . . 1<br>NO . . . . 2<br>(GO TO 203 FOR NEXT CHILD OR, IF NO MORE CHILDREN, GO TO 214)	YES . . . . 1<br>NO . . . . 2<br>(GO TO 203 FOR NEXT CHILD OR, IF NO MORE CHILDREN, GO TO 214)	YES . . . . 1<br>NO . . . . 2<br>(GO TO 203 IN FIRST COLUMN OF NEW QUESTIONNAIRE; OR, IF NO MORE CHILDREN, GO TO 214)
205	WEIGHT IN KILOGRAMS<br>**(10)**	KG. ☐☐ . ☐☐<br><br>NOT PRESENT . . . . . 9994<br>REFUSED . . . . 9995<br>OTHER 9996	KG. ☐☐ . ☐☐<br><br>NOT PRESENT . . . . . 9994<br>REFUSED . . . . 9995<br>OTHER 9996	KG. ☐☐ . ☐☐<br><br>NOT PRESENT . . . . . 9994<br>REFUSED . . . . 9995<br>OTHER 9996
206	HEIGHT IN CENTIMETERS	CM. ☐☐☐ . ☐<br><br>NOT PRESENT . . . 9994<br>REFUSED . . . . 9995<br>OTHER 9996	CM. ☐☐☐ . ☐<br><br>NOT PRESENT . . . 9994<br>REFUSED . . . . 9995<br>OTHER 9996	CM. ☐☐☐ . ☐<br><br>NOT PRESENT . . . 9994<br>REFUSED . . . . 9995<br>OTHER 9996
207	MEASURED LYING DOWN OR STANDING UP?	LYING DOWN . . . . 1<br>STANDING UP . . . . 2<br>NOT MEASURED 3	LYING DOWN . . . . 1<br>STANDING UP . . . . 2<br>NOT MEASURED 3	LYING DOWN . . . . 1<br>STANDING UP . . . . 2<br>NOT MEASURED 3
209	LINE NUMBER OF PARENT/ OTHER ADULT RESPONSIBLE FOR THE CHILD (FROM COLUMN 1 OF HOUSEHOLD SCHEDULE). RECORD '00' IF NOT LISTED.	LINE NUMBER . . . . . ☐☐	LINE NUMBER . . . . . ☐☐	LINE NUMBER . . . . . ☐☐

		CHILD 4	CHILD 5	CHILD 6
210	ASK CONSENT FOR ANEMIA TEST FROM PARENT/OTHER ADULT IDENTIFIED IN 209 AS RESPONSIBLE FOR CHILD.	As part of this survey, we are asking people all over the country to take an anemia test. Anemia is a serious health problem that usually results from poor nutrition, infection, or chronic disease. This survey will assist the government to develop programs to prevent and treat anemia.<br><br>We ask that all children born in 2016 **(9)** or later take part in anemia testing in this survey and give a few drops of blood from a finger or heel. The equipment used to take the blood is clean and completely safe. It has never been used before and will be thrown away after each test.<br><br>The blood will be tested for anemia immediately, and the result told to you right away. The result will be kept strictly confidential and will not be shared with anyone other than members of our survey team.<br>Do you have any questions?<br>You can say yes to the test, or you can say no. It is up to you to decide.<br>Will you allow (NAME OF CHILD) to participate in the anemia test?		
211	CIRCLE THE APPROPRIATE CODE AND SIGN YOUR NAME.	GRANTED . . . . 1<br>(SIGN)<br>REFUSED 2	GRANTED . . . . 1<br>(SIGN)<br>REFUSED 2	GRANTED . . . . 1<br>(SIGN)<br>REFUSED 2
212	RECORD HEMOGLOBIN LEVEL HERE AND IN THE ANEMIA PAMPHLET **(11)**.	G/DL ☐☐.☐<br><br>NOT PRESENT . . . . . . .994<br>REFUSED . . . . .995<br>OTHER 996	G/DL ☐☐.☐<br><br>NOT PRESENT . . . . . . .994<br>REFUSED . . . . .995<br>OTHER 996	G/DL ☐☐.☐<br><br>NOT PRESENT . . . . . . .994<br>REFUSED . . . . .995<br>OTHER 996
213	GO BACK TO 203 IN NEXT COLUMN OF THIS QUESTIONNAIRE OR IN THE FIRST COLUMN OF AN ADDITIONAL QUESTIONNAIRE; IF NO MORE CHILDREN, GO TO 214.			

FOLLOW-UP INFORMATION AND END OF SURVEY

FOLLOW-UP INFORMATION

IS THE WOMAN WHO IS ANSWERING THIS SECTION THE SAME PERSON WHO ANSWERED THE HOUSEHOLD SECTIOI Y N

IF YES, SKIP BELOW TO END OF SURVEY.

Thank you for participating in this survey. We may contact your household again in the future to learn more about how life changes for Malawian families. Could you please give us information about two people who **DO NOT LIVE IN THE HOUSEHOLD** and who would know where you or other household members are, or how to reach you, in the future?

CONTACT 1

FULL NAME: ____________________

RELATIONSHIP TO YOU: ____________________

FULL ADDRESS: ____________________

____

____

PHONE NUMBER: ____________________

E-MAIL ADDRESS: ____________________

CONTACT 2

FULL NAME: ____________________

RELATIONSHIP TO YOU: ____________________

FULL ADDRESS: ____________________

____

____

PHONE NUMBER: ____________________

E-MAIL ADDRESS: ____________________

OTHER HOUSEHOLD CONTACT:

What is the name and phone number of someone else within your household?

NAME: ____________________

PHONE: ____________________

RELATIONSHIP TO YOU: ____________________

PLANS TO MOVE:

Does your family/household have any plans to move in the next two years? YES NO

IF YES: Where do you plan to move to? ADDRESS: ____________________

____

____

PHOTO OF RESPONDENT:

With your permission, I would now like to take a photo of you. Taking your photo will help us to find you again in the future.

May I take your photo now?

YES NO

IF YES, PLEASE TAKE A PHOTO OF THE RESPONDENT. BE SURE TO CLEARLY CAPTURE THE RESPONDENT'S FACE FROM THE NECK UP.

END OF SURVEY:

You have now reached the end of the survey. Thank you for your time and ideas. This has been extremely helpful. As I said in the beginning, the purpose of this discussion was to help me learn about women's health in Malawi. I also want to remind you that all of your responses will remain confidential. Finally, I ask that you not share the details of what was said here. If you are asked about this study, please use only general descriptions, such as "I was gathering information about women and men and health issues."

How does that sound to you? Do you have any further questions for me at this time? If you would like to speak with me in private, I will stay here after we end.

Thank you again for your help.

SIGNATURE OF INTERVIEWER:______________________ DATE: ______________

END. GO TO INTERVIEWER OBSERVATIONS.

INTERVIEWER'S OBSERVATIONS

TO BE FILLED IN AFTER COMPLETING INTERVIEW

COMMENTS ABOUT RESPONDENT:

COMMENTS ON SPECIFIC QUESTIONS:

ANY OTHER COMMENTS:

SUPERVISOR'S OBSERVATIONS

NAME OF SUPERVISOR: ______________________ DATE: ______________________

EDITOR'S OBSERVATIONS

NAME OF EDITOR: ______________________ DATE: ______________________
