# Supplementary material for: The Effect of Improved Access to Family Planning on Postpartum Women: Protocol for a Randomized Controlled Trial
Source: JMIR Res Protoc. 2020 Aug 14;9(8):e16697. doi: 10.2196/16697 (PMC7455875; doi:10.2196/16697)
Supplement: Multimedia Appendix 2 [file resprot_v9i8e16697_app2.pdf]

Contraceptive Side Effects: Some Myths and Facts

Myth: Using contraceptive methods (pill, injectable, implant) causes cancer.

Fact: Using contraceptive methods may in fact decrease the risk of some cancers.

Myth: Using contraceptive methods causes infertility.

Fact: Using contraceptive methods does not cause infertility, even if they have been used for a long time. A couple can become pregnant soon after they stop.

Myth: Using contraceptive methods causes birth defects.

Fact: Using contraceptive methods does not cause birth defects.

Myth: After insertion, the IUD / implant travels to other parts of the body.

Fact: The IUD / implant does not travel to other parts of the body.

Myth: Using contraceptive methods reduces sexual desire / sexual pleasure.

Fact: Using contraceptive methods does not reduce sexual desire or interfere with sexual pleasure.

Myth: Contraceptive methods cannot be used while a baby is breastfeeding.

Fact: Certain contraceptives can be used while a baby is breastfeeding.

Talk to your health provider and visit your local family planning clinic today!

Effectively planning your family today can improve your health, your children's health, and your family's future tomorrow.

Visit your local family planning clinic today to learn more

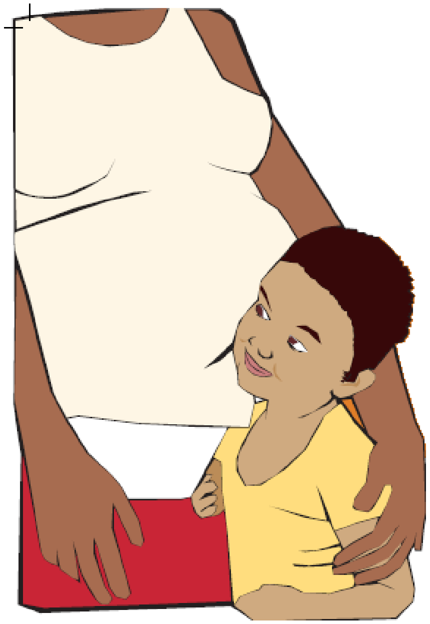

This guide was developed as part of the Malawi Family Planning Study (MFPS), located at: Innovations for Poverty Action (IPA), Area 47/2/200, Lilongwe, Malawi
Phone: +265 1762424, E-mail: cfarver@poverty-action.org

Planning Your Life After Birth

A Family Planning Guide for New and Expecting Parents

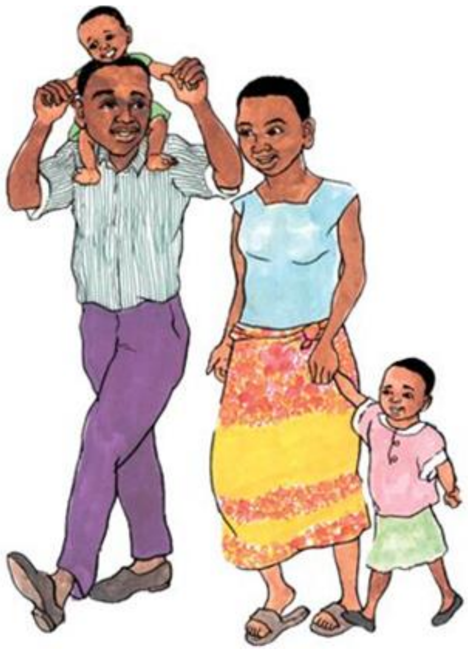

Your Return to Fertility

- A new mother can become pregnant again as soon as 3 weeks after her delivery!
- Family planning through breastfeeding can help prevent pregnancy for up to 6 months
- **Three** requirements for using breastfeeding as family planning correctly:
    1. Continuous, exclusive breastfeeding
    2. No return of menses (period)
    3. Up to 6 months postpartum only
- Mothers who are not breastfeeding are recommended to start a family planning method after 3 weeks from delivery.

If you just had a baby, when is a good time to get pregnant again?

- You should wait at least 2 years before trying to get pregnant again

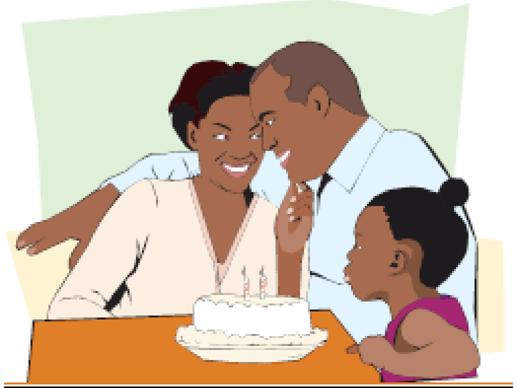

Images for this guide were obtained from: USAID ESD Project, USAID K4Health, University Research Co (URC), Google Images, and other sources.

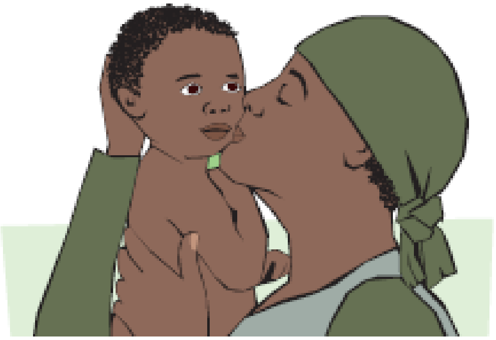

Why should you wait for 2 years?

- Mothers who wait for two years are less likely to die from childbirth.
- Children who are healthily spaced are:
    - Less likely to die during childbirth
    - Less likely to be born prematurely
    - Less likely to be underweight
    - More likely to grow up bigger and healthier

How can you healthily space and time your next birth?

Correctly and consistently use a family planning method of your choice

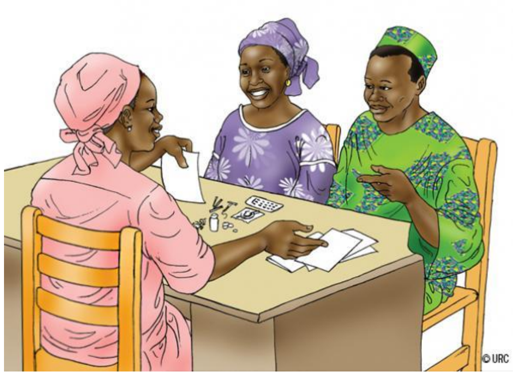

Methods for New Parents

- Different types of contraceptive methods are safe and available for new parents:

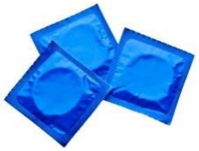

Condom

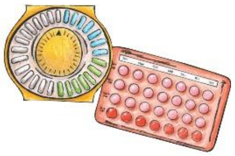

Oral Contraceptive Pill

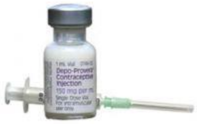

Injectable

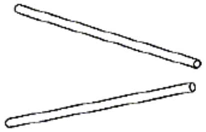

Implant

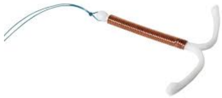

IUD

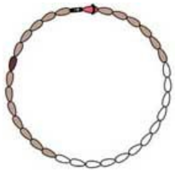

Standard Days Method

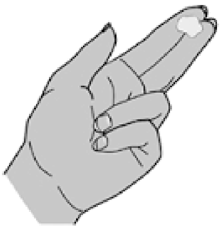

Two Day Method

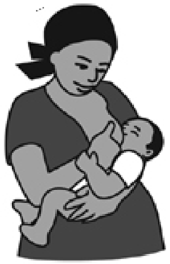

LAM

Visit your local family planning clinic today to learn more
