# Supplementary material for: The Effect of Improved Access to Family Planning on Postpartum Women: Protocol for a Randomized Controlled Trial
Source: JMIR Res Protoc. 2020 Aug 14;9(8):e16697. doi: 10.2196/16697 (PMC7455875; doi:10.2196/16697)
Supplement: Multimedia Appendix 3 [file resprot_v9i8e16697_app3.pdf]

Malawi Family Planning Study – Emergency Transport Voucher

[INSERT CLIENT PHOTO HERE]

This emergency transport voucher covers the cost of taxi fare for **one ride** from the client's pickup location to a clinic, hospital, or health facility. This voucher **will not** cover transport to any other venue other than a health facility. The date of expiration of this voucher is:

______ / ______ / ______ (DD / MM / YY)

To redeem this voucher, or if you have any questions or concerns, please call Viola Nyirongo or Violet Chitsulo at:

Viola Nyirongo: 0888680476 / 0999762265 **Violet Chitsulo: 0888688500 / 0994321890**

Client Name: [INSERT CLIENT NAME]

Client Program ID: [INSERT CLIENT PROGRAM ID]

Client Phone Number: [INSERT CLIENT PHONE NUMBER]

Date: ______ / ______ / ______ (DD / MM / YY)

Time: ______ : ______ (HH : MM) AM / PM

Total Fare: ______ |______ |______ |______ |______ MWK

MANAGER USE ONLY

DATE RECEIVED: ____________

REIMBURSED? Y N

MANAGER SIGNATURE

____

Driver Name: ______________________________

Driver Phone Number: 0 |____ |____ |____ |____ |____ |____ |____ |____ |____

Malawi Family Planning Study – Emergency Transport Voucher

[INSERT CLIENT PHOTO HERE]

This emergency transport voucher covers the cost of taxi fare for **one ride** from the client's pickup location to a clinic, hospital, or health facility. This voucher **will not** cover transport to any other venue other than a health facility. The date of expiration of this voucher is:

______ / ______ / ______ (DD / MM / YY)

To redeem this voucher, or if you have any questions or concerns, please call Viola Nyirongo or Violet Chitsulo at:

Viola Nyirongo: 0888680476 / 0999762265 **Violet Chitsulo: 0888688500 / 0994321890**

Client Name: [INSERT CLIENT NAME]

Client Program ID: [INSERT CLIENT PROGRAM ID]

Client Phone Number: [INSERT CLIENT PHONE NUMBER]

Date: ______ / ______ / ______ (DD / MM / YY)

Time: ______ : ______ (HH : MM) AM / PM

Total Fare: ______ |______ |______ |______ |______ MWK

MANAGER USE ONLY

DATE RECEIVED: ____________

REIMBURSED? Y N

MANAGER SIGNATURE

____

Driver Name: ______________________________

Driver Phone Number: 0 |____ |____ |____ |____ |____ |____ |____ |____ |____
